# Spatiotemporal survival analysis for movement trajectory tracking in virtual reality

**DOI:** 10.1038/s41598-025-91471-5

**Published:** 2025-03-01

**Authors:** Omar Fahmi Jubran, Maximilian Philipp Wolkersdorfer, Vera Eymann, Nicole Burkard, Daniela Czernochowski, Marc Herrlich, Cees van Leeuwen, Thomas Lachmann

**Affiliations:** 1 Center for Cognitive Science, University of Kaiserslautern-Landau, 67663 Kaiserslautern, Germany; 2 Experimental Psychology, University of Kaiserslautern-Landau, 67663 Kaiserslautern, Germany; 3 Deutsches Forschungszentrum für Künstliche Intelligenz (DFKI), Oldenburg, Germany; 4 Brain and Cognition Research Unit, Faculty of Psychology and Educational Sciences, KU Leuven, Leuven, Belgium; 5 Centro de Investigación Nebrija en Cognición, Universidad Nebrija, 28015 Madrid, Spain

**Keywords:** Psychology, Human behaviour

## Abstract

We present a novel method for analyzing response trajectory tracking data. Limiting behavioral experiments to discrete, key-press response measures, such as reaction times and accuracy, is unsatisfactory for observing the ongoing dynamics of cognition. We assessed the utility of continuous response tracking in Virtual Reality (VR) by comparing it to key-press responses in a classical N-back matching task. For elucidatory purposes, in both classical and VR versions of the task we first worked through analyses of discrete measures, before drawing information from the continuous trajectory tracking data in VR. Classical ANOVAs reproduced effects of visual working memory load in an N-back task. Violations of ANOVA assumptions suggested effects were buried in the noise; some of these were revealed in subsequent survival analyses, namely frequency neglect (a strong preference for match responses despite the infrequency of this response category) in the fast responses and category frequency-tuned response in the slow responses. Spatiotemporal survival analysis (StSA), our newly proposed method of analyzing response trajectories, revealed that all these effects also occur in the VR conditions. In addition, initial divergences towards the wrong responses were corrected later in the course of trajectories in the non-Match trials. While the StSA method is theory-free and can be used for exploratory purposes, we present examples of hypothesis testing in StSA.

## Introduction

Human perception and cognition are dynamic processes and need to be analyzed as such. Spivey and Dale (2006) argue that Reaction Times (RT) and accuracy measures are insufficient for understanding cognition, because they provide only single snapshots of its time-course^1^. Instead, trajectory tracking allows us to study continuous behavior in space and time (for a review see,^2^ ). In experimental settings, trajectory tracking provides a wealth of information beyond single response measures, promising insight into the time course of decision-making, response preparation, and execution.

To take benefit of this approach, we will propose a new analysis method for trajectory tracking data. Trajectory tracking dates back to before 1900^3^. The earliest studies used pen and paper, while since the 1970s, digitizing tablets, stylus and mouse tracking have been used (for a review, see^4^). These studies were mostly limited to pointing movements on two-dimensional surfaces^5,6^. Observing motor responses during mouse cursor tracking has been effective in tracing the dynamics of a mental process such as phonological awareness^7^, face perception^8^, and working memory^9^.

Despite the advantages of tracking pencil or mouse movements, the relationship between the movements and their effects on the screen, such as grasping an object, has remained conventional and artificial, limiting their usefulness^10^. Mouse tracking studies are limited by design factors, namely response dictation and mouse sensitivity, and face differences in opinion about the optimal starting procedure (for a detailed review, see^11^ ). In contrast, the advent of virtual reality (VR) technology not only allows for the presentation of realistically rendered three-dimensional objects but also allows participants to navigate freely within the VR space^12^.

The added value of tracking responses in VR has been demonstrated in traditional experiments. These experiments enabled the researchers to examine the continuity between the results of classical laboratory experiments, typically conducted on a personal computer (PC), and a VR version. We chose a well-known working memory (WM;^13–16^) paradigm, viz. the N-back task^15^. Participants are presented with a series of items and, at random intervals, are prompted to indicate by keystroke whether the currently displayed item matches the one presented N steps back. The value of N to which a participant still responds correctly represents an estimate of WM capacity. This task was chosen as a typical example of a continuous cognition task that involves perceptual processing, decision-making (two-alternative forced-choice), motor preparation, and execution, with a well-established effect of task difficulty (as a function of N), while the analyses classically still involve discrete measures such as accuracy or RT.

We will first compare accuracy and RT from a classical version of the N-back task with analogous measures obtained from the movement trajectories in a VR-based version. We illustrate their shortcomings (in particular violations of assumptions and lack of power in Analyses of Variance -ANOVA). We discuss efforts to remedy these, i.e. survival analysis (SA) and the new problems it brings regarding power, i.e., limited data. We then introduce the method of Spatiotemporal Survival Analysis (StSA). We claim that this method allows us to go beyond classical results in understanding decision processes.

## Results

### Means analyses using ANOVA

A total of 9,120 trials were initially available for analysis. Trials at the beginning of the N-back blocks are task-irrelevant and are thus removed from the analysis, leaving 8664 trials, 4332 for classical, and 4332 for VR.

We first compared RT(C) to RT(VR) and next accuracy(C) to accuracy(VR), using three-way ANOVAs, with the factors Device (PC vs VR), N-back (1-3) and Trial Type (Match vs non-Match). Data were trimmed for outliers using quartile outlier rejection, (1.5 Interquartile range), separately for each experimental condition, leaving 4091 (5.56% removed) trials for classical and 4034 (6.87% removed) trials for VR. Further, trials outside the range of the mean plus two times standard deviation were excluded from the analysis, leaving 3884 (5.05% removed) trials for Classical and 3841 (4.78% removed) trials for VR. Finally, error trials were excluded from RT(C) (6.2% removed) and RT(VR) (7.75% removed). Results for untrimmed versions of the data are available in the supplementary materials.

#### Reaction time (RT)

**Fig. 1:**
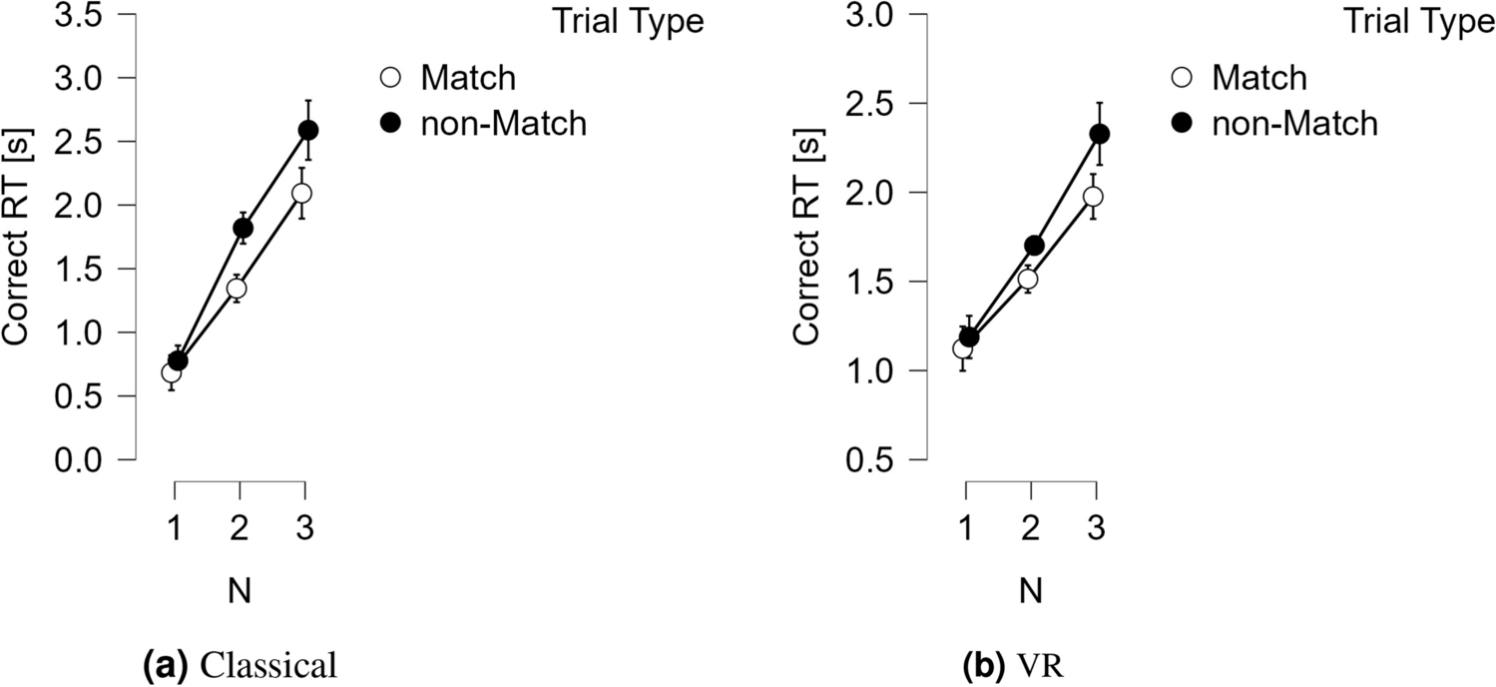
Averages for the classical reaction times, RT(C) (**a**) and their VR counterpart, RT(VR) (**b**). Vertical bars represent standard errors. For the N1 N2 and N3 N-back conditions, for both Match and non-Match trials.

**Fig. 2:**
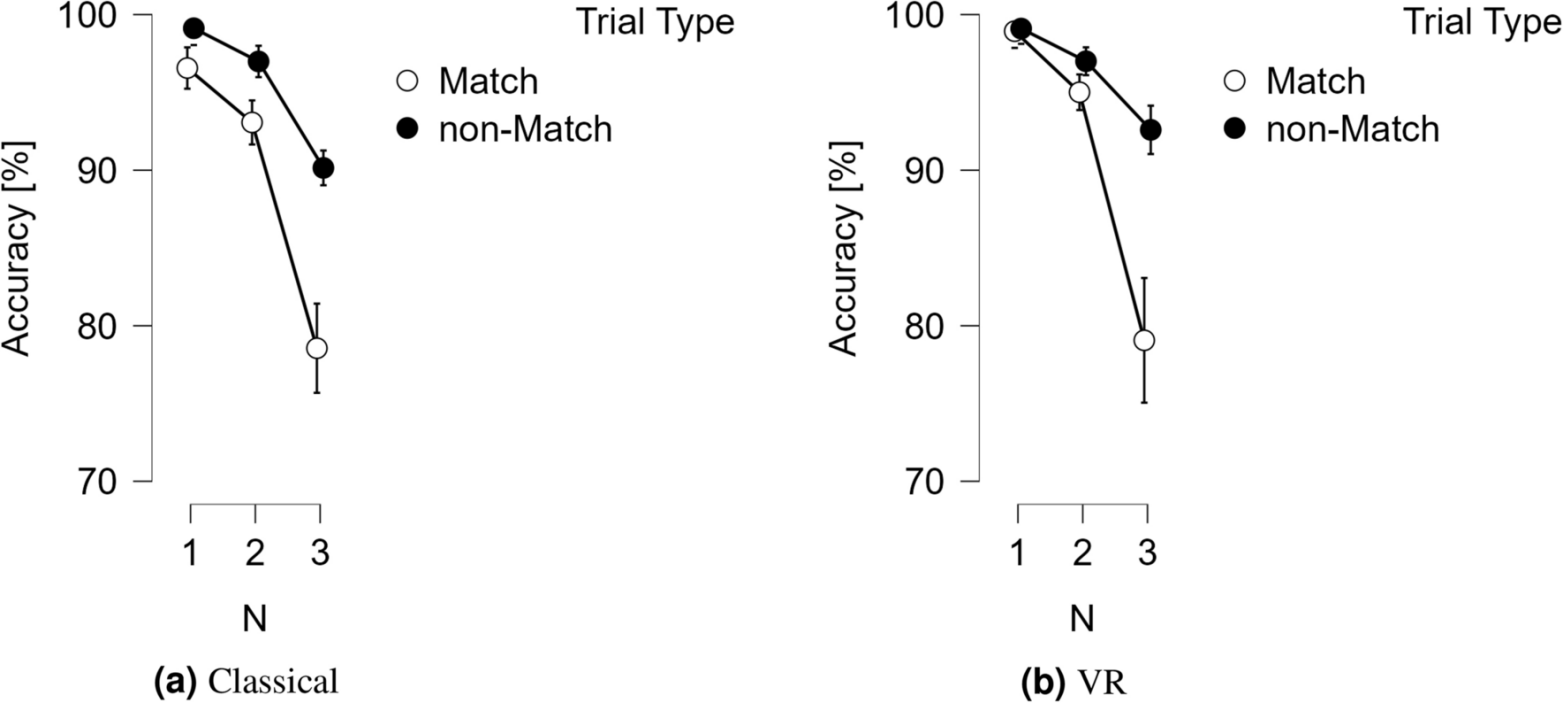
Average for the classical accuracy, accuracy(C) (**a**) and its’ VR counterpart, accuracy(VR) (**b**). Vertical bars represent standard errors. For the N1 N2 and N3 N-back conditions, for both Match and non-Match trials.

As shown in Figs. 1 and 2, there was no main effect of Device [*F*(1, 18) = 0.996, *p* = .332], while main effects of N-back [*F*(2, 36) = 26.446, *p* < .001, $\eta ^2 = .434$], and Trial Type [*F*(1, 18) = 12.692, *p* = .002, $\eta ^2 = .030$] were obtained. There were interactions between Device and N-back [*F*(2, 36) = 8.267, *p* = .005, $\eta ^2 = .027$], as well as between Trial type and N-back [*F*(2, 36) = 6.066, *p* = .013, $\eta ^2 = .009$]. However, there was no interaction between Device and Trial Type [*F*(1, 18) = 2.478, *p* = .133] and no three-way interaction between Device, Trial Type, and N-back [*F*(2, 36) = 1.196, *p* = .307].

To investigate the interaction between Device and N-back, we conducted post hoc tests. The tests revealed a significant difference in N1 between RT(C) and RT(VR) ($M_{\text {diff}} = 0.425$, $SE = 0.121$, $t = -3.515$, $p = 0.006$). No significant effects were found in N2 or N3 between RT(C) and RT(VR).

#### Accuracy

There was no main effect of Device [*F*(1, 18) = 1.471, *p* = .241]. However, we obtained main effects of N-back [*F*(2, 36) = 35.973, *p* < .001, $\eta ^2 = .328$] and Trial Type [*F*(1, 18) = 17.096, *p* < .001, $\eta ^2 = .079$]. There was no interaction between Device and Trial Type [*F*(1, 18) = 0.410, *p* = .530] and no interaction between Device and N-back [*F*(2, 36) = 0.035, *p* = .909]. However, we obtained an interaction between Trial type and N-back [*F*(2, 36) = 10.574, *p* = .003, $\eta ^2 = .061$]. There was no three-way interaction between Device, Trial type, and N-back [*F*(2, 36) = 0.751, *p* = .424].

#### Summary and discussion

Both in the classical and VR version of the N-back task, ANOVAs confirmed that as N increased, responses overall became slower and less accurate. The effect of N-back was more pronounced in the classical version than in VR. Responses were faster but less accurate in Match than in non-Match trials. RT(VR) and accuracy(VR) thus, consistently replicate all classical effects of the N-back task^17^. This, despite the large differences between RT(C) and RT(VR) due to the additional response requirements of the latter. These differences are revealed for N = 1 in the posthoc tests; for larger Ns, they are likely absorbed in the motor execution component of RT(VR). We might expect this to be observed as a tendency of the variance of RT(VR) to increase with larger N. Increases are usually tolerated, but technically constitute a violation of ANOVA assumptions. Variance differences may conceal effects of interest in RT(VR) especially with overlapping motor execution and decision-making.

#### Variance of the means

**Fig. 3:**
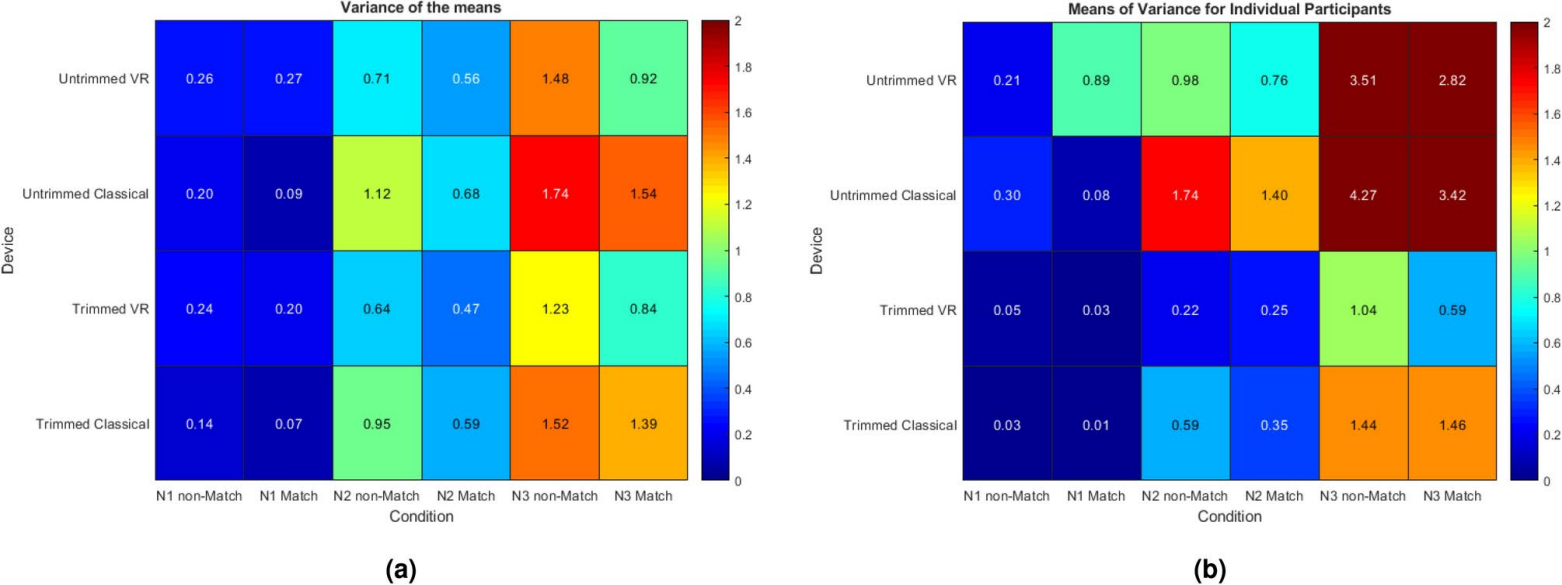
Trimmed and untrimmed data variance of the means (**a**) and the means of individual subject variances (**b**) across N1 N2 and N3 N-back conditions, for both Match and non-Match trials.

In the ANOVA, we observed violations of both the equal variance and sphericity assumptions. We present the variances of the means (Fig. 3a), and the means of individual subject variances across conditions (Fig. 3b), for both the trimmed and untrimmed data.

Figure 3 shows that across all conditions, variances tend to increase with task difficulty: lowest for N1, larger for N2, and still larger for N3. Non-matches show a larger variance than Matches, except notably for N1. Despite our expectation, the increases are similar for RT(C) and RT(VR).

Trimming reduces the variance in all conditions. However, we still observe differences of variances across conditions. In addition, this procedure has little to no effect on sphericity (comparing assumption checks tables in the supplementary materials). Violations of sphericity could be corrected by Greenhouse-Geisser but call the results of the ANOVA into question (see Armstrong, 2017). Note that the concerns about increasing variance apply equally to the RT (VR) and RT(C).

ANOVA assumes that error variance is additive to the task effect. In fact, the noise is more likely to be multiplicative. The more difficult the task, the more room for diversions, hesitations, and self-corrections that would increase the noise. Not all individuals are subject to the same degree of hesitation, self-correction, etc., as some will find the N3 task still relatively easy while others struggle. The unequal variances hide such processes from view.

### Survival analysis (SA)

Survival Analyses proceeded from the original, untrimmed data (Refer to the methods section for more information). We calculated sample-based estimates of *h*(t), *S*(t), and *ca*(t) for (N-back (1-3) and Trial Type (Match vs non-Match)), both for RT(C) and RT(VR). To obtain a high temporal resolution, we divided the first 7000 milliseconds (ms) after item onset into 70 discrete temporal bins of 100 ms (indexed t = 1-70; e.g., bin 37 = bin 3,700 = (3600,3700]). Trials after 7000 ms were right-censored in the graphs but included in the analyses. Thus, unlike ANOVAs ran with trimmed data, SA does not run the risk of underestimating the means and variances (for more detail, see^18^).

#### RT(C)

**Fig. 4:**
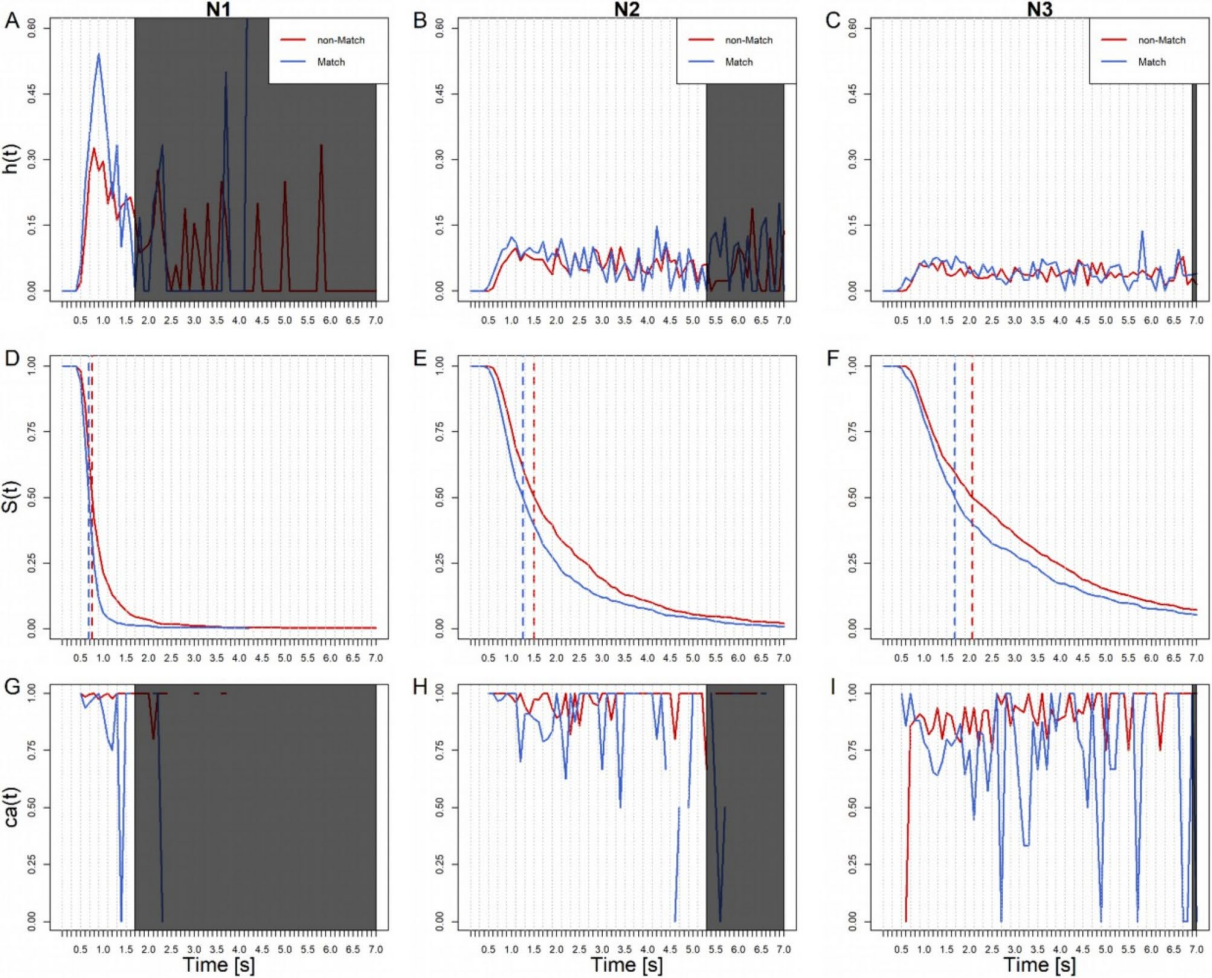
Sample-based estimates of *h*(t), *S*(t), and *ca*(t) aggregated across all participants in the classical measure, for the first 70 bins (or 7,000 ms) after item onset. Bin width equals 100 ms. Each column represents a different N-back condition (in ascending order). Blue lines represent Match trials, red lines non-Match trials. Dotted vertical lines represent median RT estimates for Match and non-Match trials. Note that the greyed areas in *h*(t) and *ca*(t) plots obscure remaining time bins that contain less than 5% of trials (*S*(t>T) = 0.05). This is to highlight the more informative left side of the distributions.

In N1, hazard rates start rising after approx. 400 ms. Between 400-1200 ms responses occur at a higher rate in the Match compared to the non-Match condition as demonstrated by hazard rates (Fig. 4a). Note that 95% of responses occur within the first 1700 ms (Fig. 4d). Remarkably, the earliest responses are 100% correct in both Match and non-Match conditions (Fig. 4g). Late responses become less accurate in the Match condition (0% accuracy at (1300,1400]) but stay at around 100% accuracy in the non-Match condition. This might suggest that, as time progresses, non-Match emerges as preferred among the remaining responses. This would be in accordance with the frequency of non-Match trials (66%) as opposed to Match trials (33%). However, this suggestion is inconclusive, as too many responses have already occurred at this point (Fig. 4d).

As N increases, first responses still occur after about 400 ms (Fig. 4d and c). However, hazard rates flatten out compared to N1. This is evidence that the decision-making process takes more time as task difficulty increases. Overall accuracy decreases with N, as more responses occur at lower conditional accuracy (as seen in *S*(t) and *ca*(t) in Fig. 4). Accordingly, in N2, 95% of response completion takes 5300 ms and in N3, 6900 ms (Fig. 4e and f). Note that as in N1, hazards are higher in the Match compared to the non-Match condition for almost the entire distribution. While conditional accuracy develops similarly to N1 (early correct responses, followed by the preference of non-Match responses in the remaining trials, Fig. 4h), N3 shows a remarkable difference (Fig. 4i). Earliest responses here are 0% correct in the non-Match condition, and 100% correct in the Match condition. Participants responding fast in N3 are exclusively producing Match responses. This, despite these trials being less frequent (33%) than non-Match ones (66%). For this reasons we will call this behavior frequency neglect^19^. After 800 ms non-Match responses are preferred. These late responses are more accurate in the non-Match trials than Match trials, in line with our suggestion for N1. Since this is in accordance with frequency of trial types, we call these responses probability-adjusted. This effect is still quite noisy, especially in the later responses due to the rapidly increasing sparsity of trials. Thus, it is yet impossible to determine the time window of category frequency-tuned responses.

#### RT(VR)

**Fig. 5:**
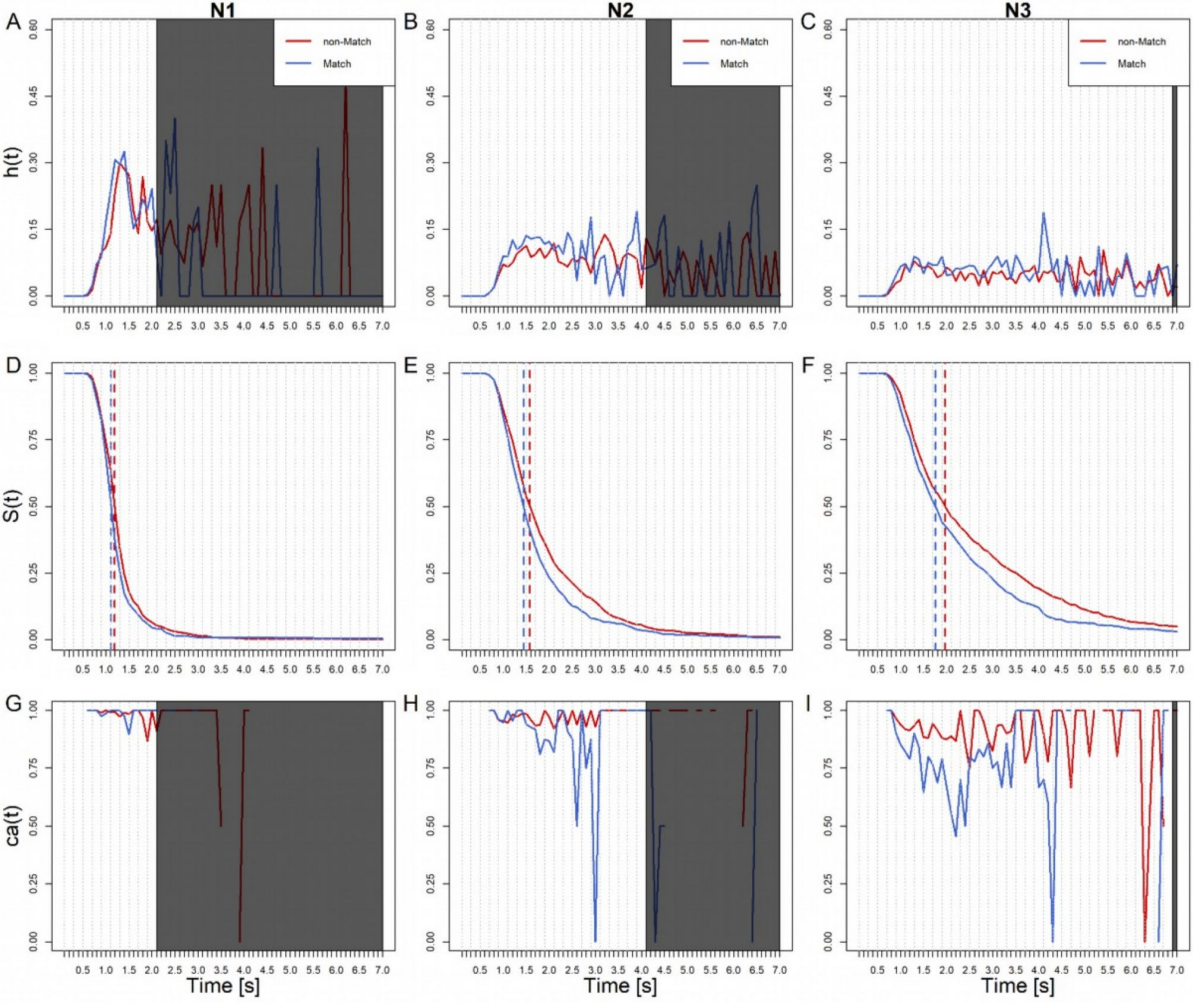
Sample-based estimates of *h*(t), *S*(t), and *ca*(t) aggregated across all participants in the VR, for the first 70 bins (or 7000 ms) after item onset. Bin width equals 100 ms. Each column represents a different N-back condition (in ascending order). Blue lines represent Match trials, red lines non-Match trials. Dotted vertical lines represent median RT estimates for Match and non-Match trials. Note that the greyed areas in *h*(t) and *ca*(t) plots obscure remaining time bins that contain less than 5% of trials (*S*(t>T) = .05). This is to highlight the more informative left side of the distributions.

In N1, the first responses occur only after 600 ms (compared to after 400 ms in RT(C)). Hazards rise at an equal rate between 600 to 900 ms in both conditions. Early responses are generally slower in RT(VR) than in RT(C), despite the ANOVA showing no effect of Device (compare Figs. 4 and 5). Responses completed before approx. 800 ms are about 100% accurate (Fig. 5g). Hazard rates are higher in the Match condition after 900 ms from item onset (Fig. 5a). This is also apparent in N2 and N3 (Fig. 5b and c). As in N1, early responses in N2 and N3 are always accurate. In contrast to RT(C), there is no indication of early frequency neglect (Fig. 5g–i). This might suggest that in VR, participants correct for frequency neglect in the course of their movement trajectory. We will test this in our subsequent analyses of the trajectories.

Accuracy drops in the Match condition around 1000 ms, indicating category frequency-tuned responses in N2 and N3, as previously seen in N1. Similar to RT(C) though, these effects are quite noisy and the trials are sparse, limiting conclusions beyond this point.

#### Summary and discussion

SA confirmed the observations in the ANOVAs that responses in Match trials were faster than non-Match trials across all devices and measures. Responses in Match trials are generally less accurate, and as N increases, response latencies increase and accuracy decreases. In addition, SA revealed evidence for early frequency neglect until approx. 800 ms in the RT(C) of N3. Match responses were preferred, despite occurring with a 33% frequency. For RT(VR) no such effect was observed, possibly because it was corrected in the course of the trajectory. Trajectory analysis should be able to test this hypothesis. From 800 ms onwards, category frequency-tuned responses occur across all RT(C) and RT(VR) conditions. The more frequent non-Match trials are now preferred. This dynamic of response times was previously obscured in the ANOVA. Note that the trial frequency effects (initial frequency neglect followed by category frequency-tuned responses) are prominent in the classical conditions. This, even though response execution requires only minimal movement.

### Spatiotemporal survival analysis (StSA) pipeline, stage 0: average trajectory data

**Fig. 6:**
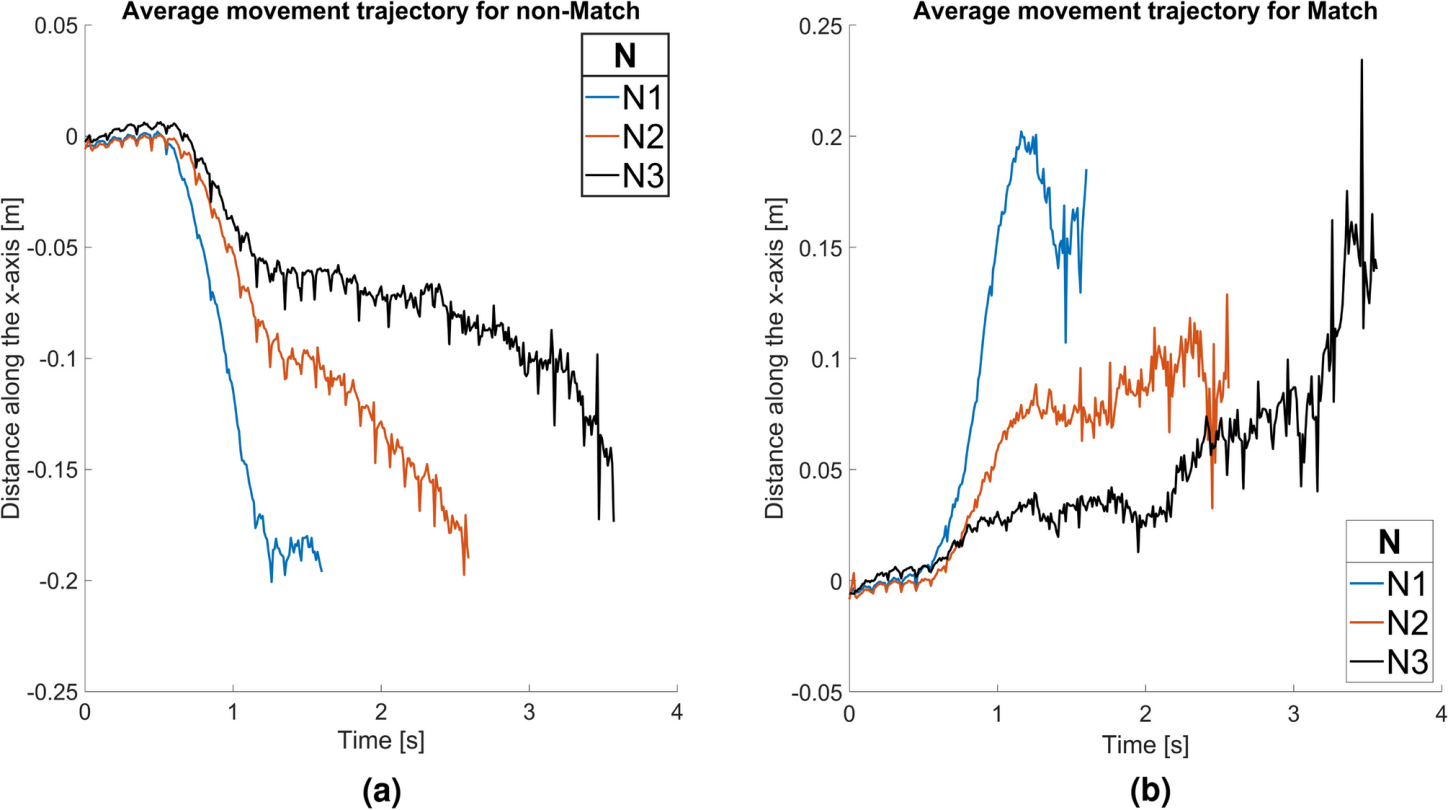
Untrimmed averaged trajectory data for the N1 N2 and N3 N-back conditions, for both Match (**a**) and non-Match (**b**). Note that with increasing time, fewer responses contribute to the averaged movement trajectory.

Like SA, Spatiotemporal Survival Analysis uses original, untrimmed data. Unlike SA, StSA investigates the spatiotemporal distribution of response trajectory data. We argued that averaged data can be misleading. Still, a first impression could be gained from averaged trajectory data. We favor an optional Stage 0 analysis of average trajectory data for each condition (Fig. 6). The x axis represents continuous time (s), while the y axis represents distance in meters (m). In all three N-back conditions, in the first 500 ms average trajectories keep close to the center of the screen. This happens, despite SA having shown a strong frequency neglect effect during that time window. Averaging trajectories runs the risk of losing such relevant information, and so an investigation of trajectory distributions is warranted. Around 500 ms, a marked deviation from the center begins to differentiate Match and non-Match trajectories. Note that in the SA, this time point marks the transition to category frequency-tuned responses. As there is a 66% majority of non-Match trials, this effect manifests as the difference in slope between Match and non-Match trajectories. Non-Match trials on average move faster to response completion in this stage. Beyond 700 ms, the trials still seem to move to completion at different rates, more slowly for the Match condition than for the non-Match condition. But this averages-based result is in fact misleading. It applies only to the few individual trials that have not yet come to completion. There are fewer of these in the Match than in Non-match condition, as the latter tends to be slower. The decrease in trials can be inferred from the jagged character of the graphs towards their end.

In both Match and non-Match conditions, the slope of deviation becomes less steep with increasing N. This difference in slope is maintained during most of the response process, showing that participants move at lower speeds throughout the more difficult conditions. The N1 average has little noise in both Match and non-Match conditions, suggesting that most trials share similar characteristics. In N2, the early part of the trajectory has little noise in the Match condition, while later parts are noisier, suggesting a higher variance in RT(VR). There is less noise in the non-Match condition resulting in a more compact distribution of trial RT(VR). For N3, this variance between trials becomes more apparent, causing the average to be close to zero for a longer time. The findings suggest that as the difficulty level increases, participants exhibit slower and more variable responses. This shows the importance of considering both speed and variance to comprehensively understand participants’ reactions across varying difficulty levels. In the next stage, we plot individual trials, exploring the differences between conditions.

#### Stage 1: single-trial data

**Fig. 7:**
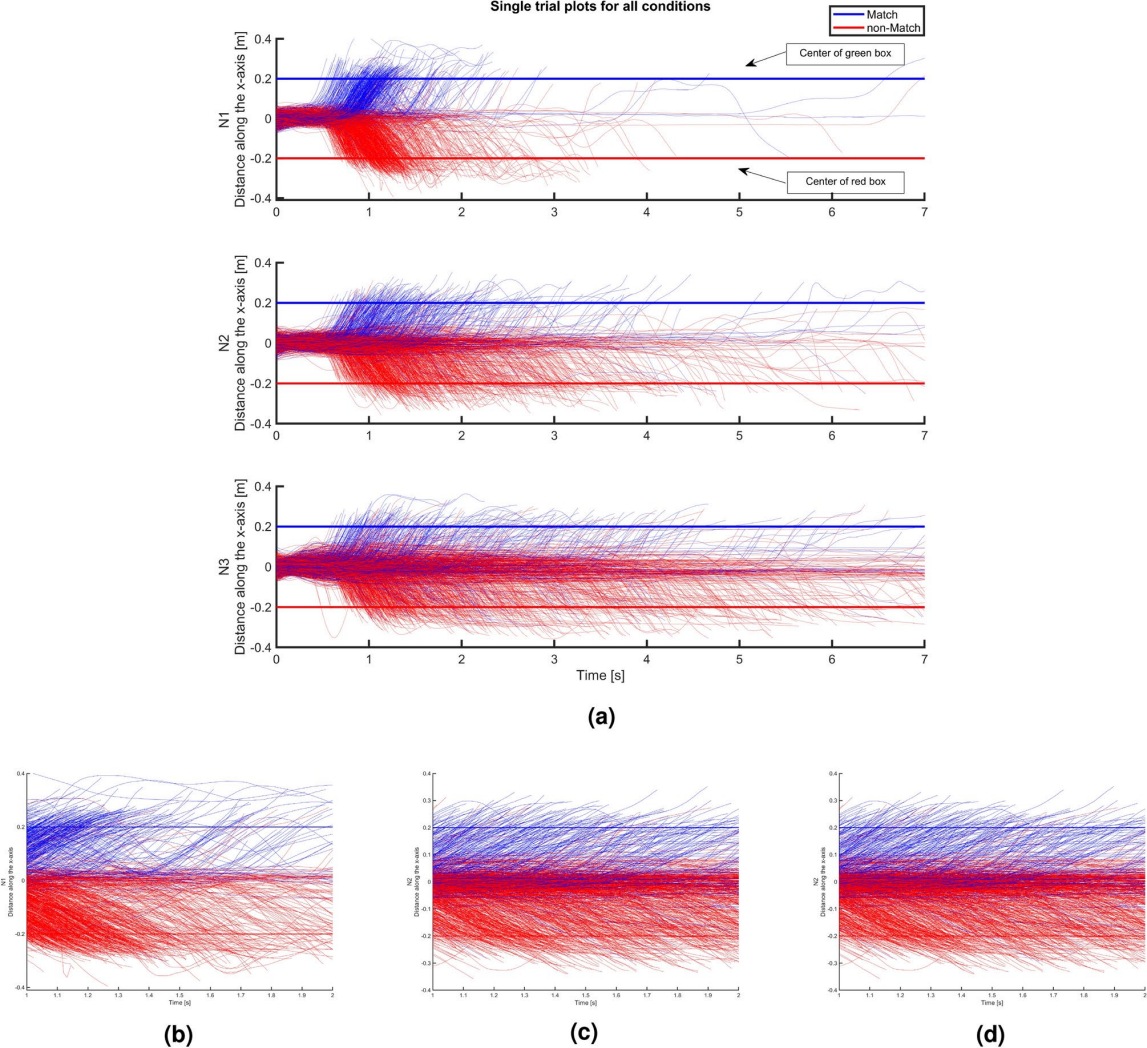
Single trial data for the N1 N2 and N3 N-back conditions, for both Match and non-Match trials. Non-Match trials are shown in red, and Match are shown in blue. The red and blue straight lines represent the centers of each response box. b-d are zoom-ins on the 1-2 seconds interval for N1-N3 respectively.

Single trial trajectories data are useful for visual inspection^20^, as this can reveal certain patterns in the data. Figure 7 shows movement trajectories for all trials in all conditions with all data included, also the error trials and those rejected as outliers in the ANOVA. The x axis represents continuous time (s), while the y axis represents distance (m). Match trials are shown in blue while non-Match ones are shown in red. The blue and red horizontal lines (between 2.2 and -2.2) indicate the location of the centers of the green and red boxes, respectively. Note that RT(VR) were registered as soon as the trajectory collided with the box, not when it reaches the center. As a result, trajectories have slightly different endpoints in space. We intend to make no adjustments for this.

All trials start at a neutral position between the boxes. If participants do not move or move on the vertical plane while not getting closer to either box, their position on the spatial axis remains at zero. At varying times, participants initiate movement to either the left or the right box. In some trials, we may notice hesitation and self-correction in movement; hesitation is indicated by slowing down or prolonged hovering at the same spatial location before moving closer to either box. Self-correction amounts to switching direction of the movement mid-trial.

In N1 most trials end before 2 seconds, with little hesitation or self-correction across the trials. In N2, while most of the trials end before 3 seconds, movement initiation is more spread out. Participants hover around the neutral position, suggesting hesitation before starting to respond. Self-correction is also more prominent in these trials. For the N3 condition, movement initiations are even more spread out. Whereas most trials end before 4 seconds, many trials are still ongoing past 7 seconds. Hovering occurs for long periods and many trials involve self-corrections.

Non-Match trials -shown in red- have a clear separation from the Match trials -shown in blue- in N1. As the task gets more difficult, two effects could be observed. First, a wider concentration of trials in the middle, indicating that the more difficult the task, the longer participants tend to stay undecided. Second, more crossings between red and blue, meaning movements in the wrong direction.

#### Stage 2: probability distribution heat maps

**Fig. 8:**
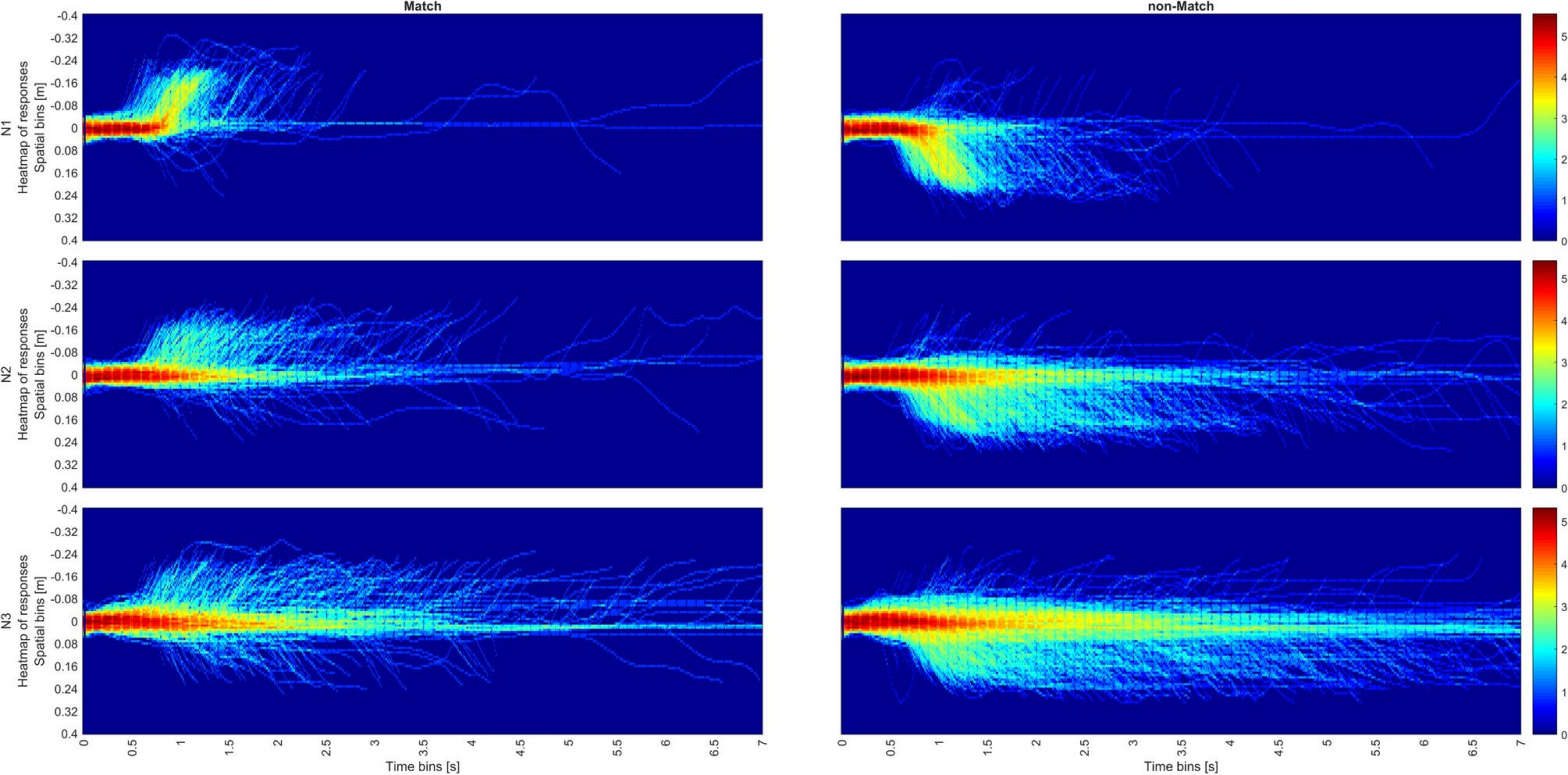
Log transformed probability distribution heat maps for discrete time and space. Left column is Match; Right column is non-Match. Each row is for each N (1:3). Log transformations are used for better visualization of differences.

Graphs of probability distributions are more informative than the averages and easier to read than single trial plots. Figure 8 shows the log-transformed 2D probability distribution heat maps of the raw data in a high-resolution heat map, which represents the frequency distribution of trajectories visiting each spatiotemporal bin. The x axis represents time bins, while the y axis represents spatial bins, totaling 101 spatial bins, ranging from -0.4-0.4 m and 701 temporal bins, ranging from 0-7 s, resulting in 70,801 discrete spatiotemporal bins. By incorporating data from single trials, these graphs show a finer level of detail compared to the averaged trajectory data.

In the N1 condition, for both Match and non-Match conditions, the probability remains notably high within the central region (between -0.08 and 0.08 m) until approximately 1 s after stimulus onset, indicating that for many trials, the initiation of movement starts around that time. These heat distributions typically appear narrow, showing little variance in trajectories.

With increasing N, trajectories tend to linger in a neutral position for a longer time. Specifically, for N2 and N3, this lasts for approximately 1.5 and 2 s, respectively. Increases in N are also associated with a more expansive distribution of responses across both space and time, most prominently observed in N3, which explains the noisy averages observed in Stage 0. This expansive distribution can indicate some overlap between decision-making and movement, where participants initiate movement in some direction early in the decision-making process, and either correct or confirm that movement later.

In non-Match conditions, the distributions are wider than in Match conditions, indicating hesitation. Additionally, there are more deviations in the wrong direction in non-Match conditions, suggesting increased self-correction. Generally, Match trials tend to show a higher probability of deviating from the center in earlier time bins compared to non-Match trials. This effect is easier to establish here than in the average trajectories.

#### Stage 3: spatiotemporal survival analysis (StSA)

The heat maps of probability distribution are the basis of StSA. Prior to the analyses, the heat maps’ resolution is lowered to ensure a sufficient number of trials per bin. Similar to SA, time is divided into discrete time bins of 100 ms. Additionally, the trajectory is divided into discrete segments, based on spatial bins of 0.01 m. There are 1,400 spatiotemporal bins (20 spatial by 70 temporal). The hazard function *H*(t), and the conditional accuracy function *CA*(t) are then calculated against movement in space, and passage of discrete time. Trajectories after 7000 ms were right-censored from the displays but included in the hazard calculations.

**Fig. 9:**
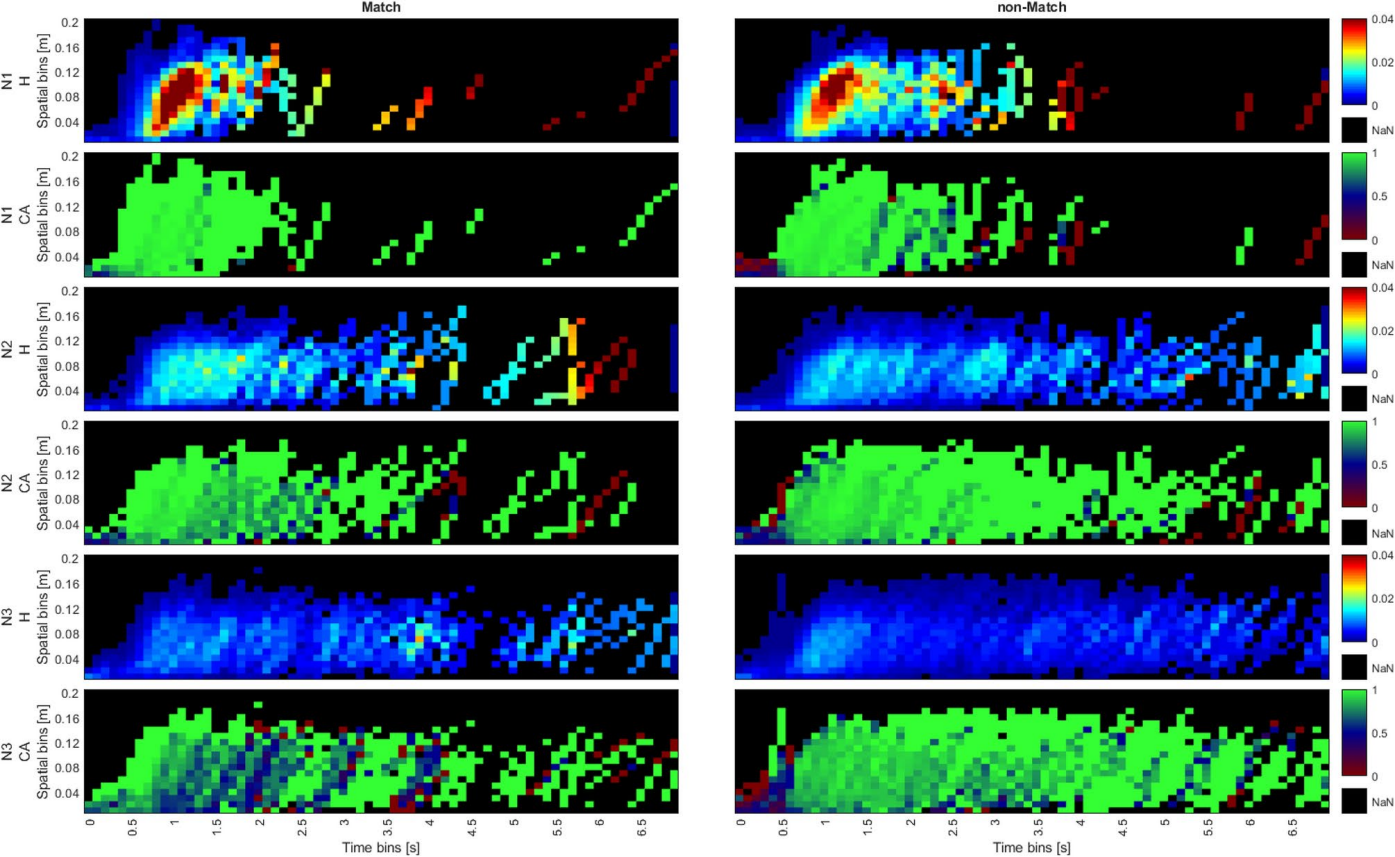
StSA plot for the N1 N2 and N3 N-back conditions, for Match (left) and non-Match conditions (right). Each row is for two dimensional Hazards (denoted as H) and conditional accuracy (denoted as CA).

Figure 9 shows the StSA of the trajectories in all the N-back (N1- N3) by Trial Type (Match, non-Match) conditions. The x axis shows time bins while the y axis shows spatial bins. There are twelve graphs in total, 6 for hazard (denoted as *H*) and 6 for conditional accuracy (denoted as *CA*). Note that, unlike SA, in StSA, *H* and *CA* do not only refer to completed trials but to segments of ongoing movement trajectories. Thus, for instance, *H* in each spatiotemporal bin is calculated in proportion to all the subsequent bins. *CA* on the other hand, is calculated for each spatiotemporal bin separately, comparing only segments within that bin regardless of their ultimate outcomes. Moving in the incorrect direction will be scored as incorrect in the relevant segments, whereas moving in the correct direction is scored as correct regardless of the trial’s ultimate accuracy(VR). Thus, Both *H* and *CA* can include information of completed and ongoing responses. Thus, this analysis can reveal ongoing processes such as self-correction that are concealed in both ANOVA and SA.

For both Match and non-Match trials, areas of interest can be identified in the hazard of the N1 conditions of Fig. 9 (red regions). Highest rates of Hazard are located between 900-1200 ms and 0.04-0.12 m. They show a high across-trial consistency of movement passing the halfway point through the movement, suggesting a sense of “warmth”^21^ before the conclusion of the movement.

**Fig. 10:**
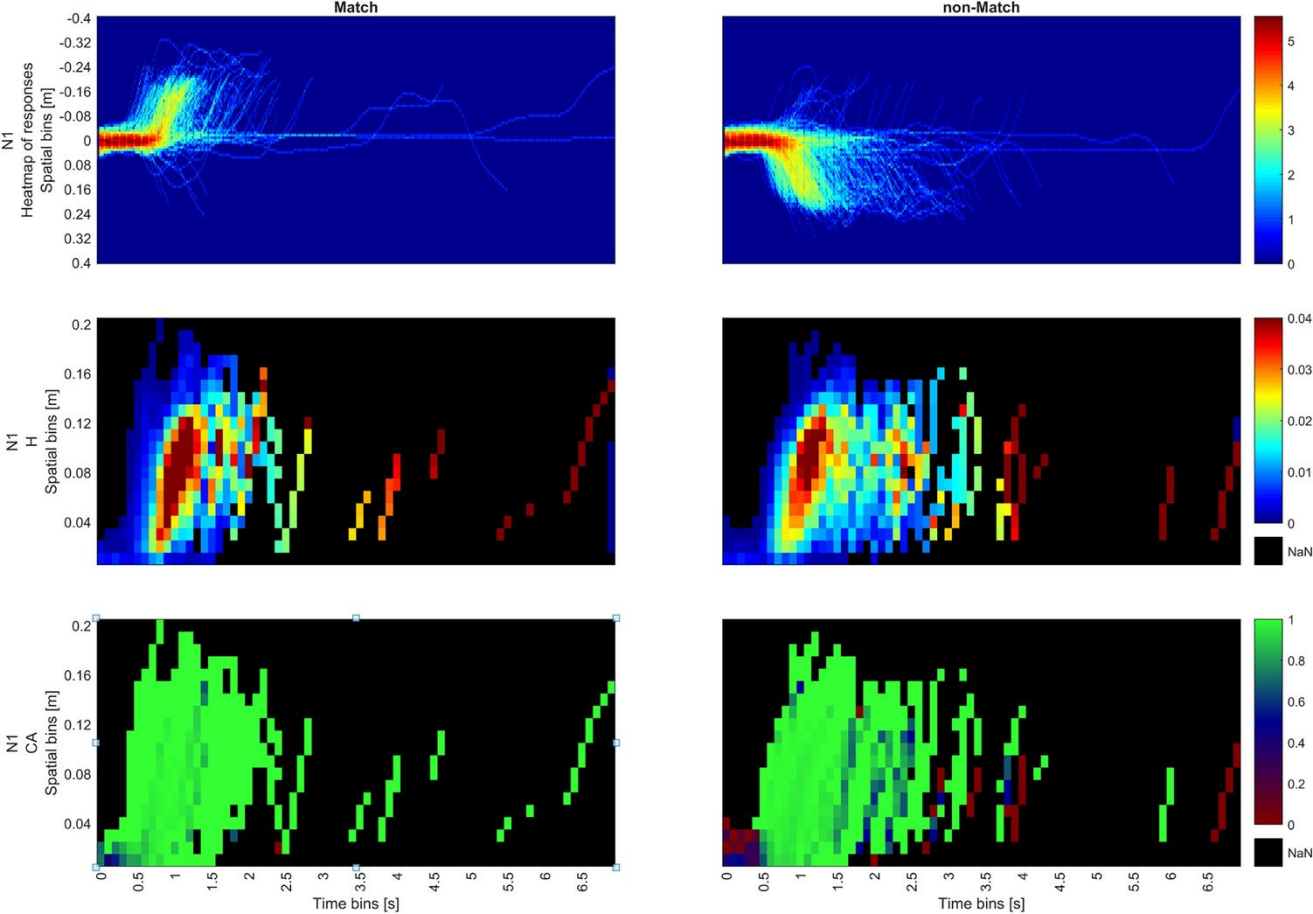
Combined plots for N1 for Match (left) and non-Match conditions (right); Top row shows the heat map of responses; middle row shows hazard rates while the bottom row shows conditional accuracy.

**Fig. 11:**
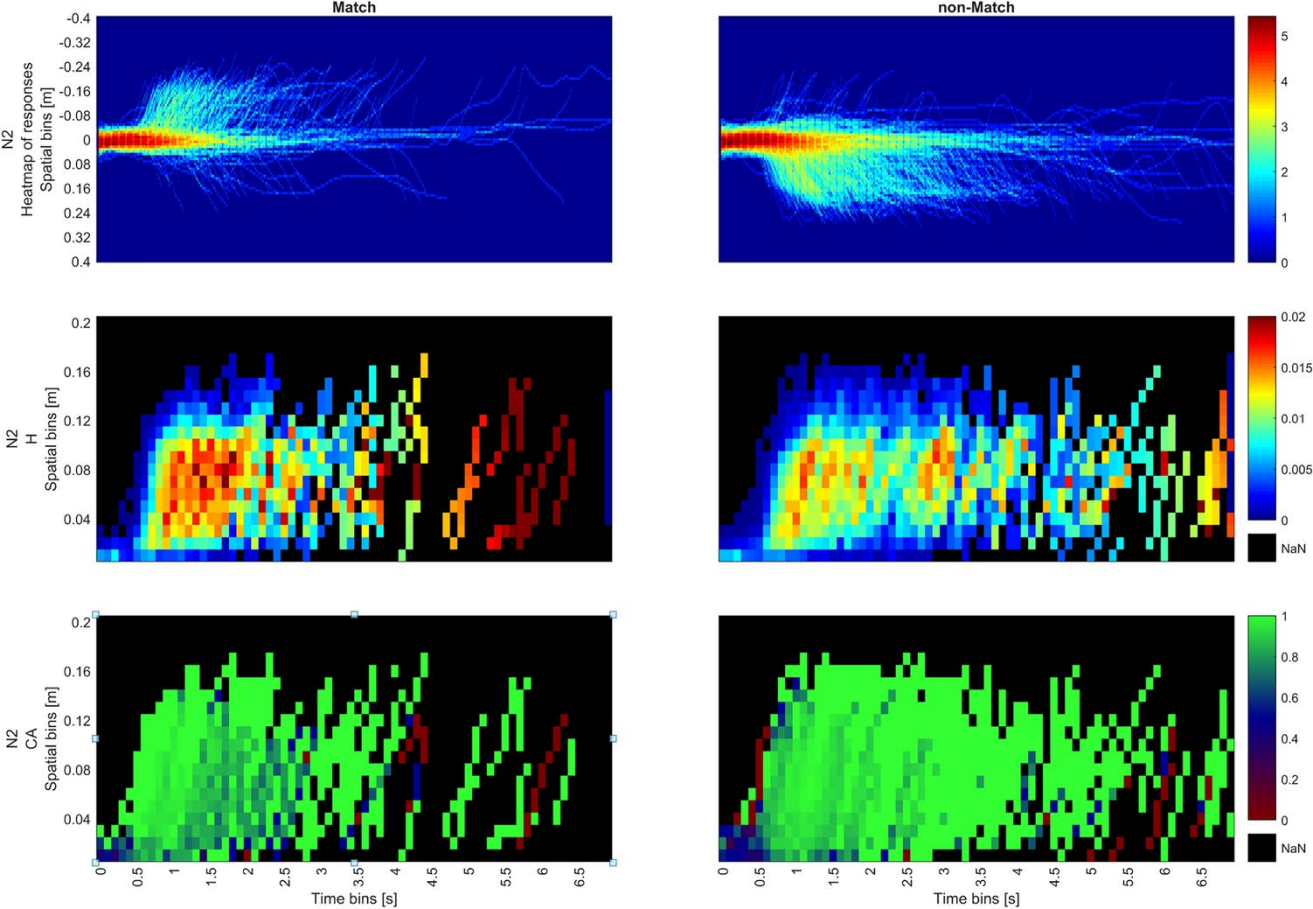
Combined plots for N2 for Match (left) and non-Match (right) conditions; Top row shows the heat map of responses; middle row shows hazard rates while the bottom row shows conditional accuracy.

**Fig. 12:**
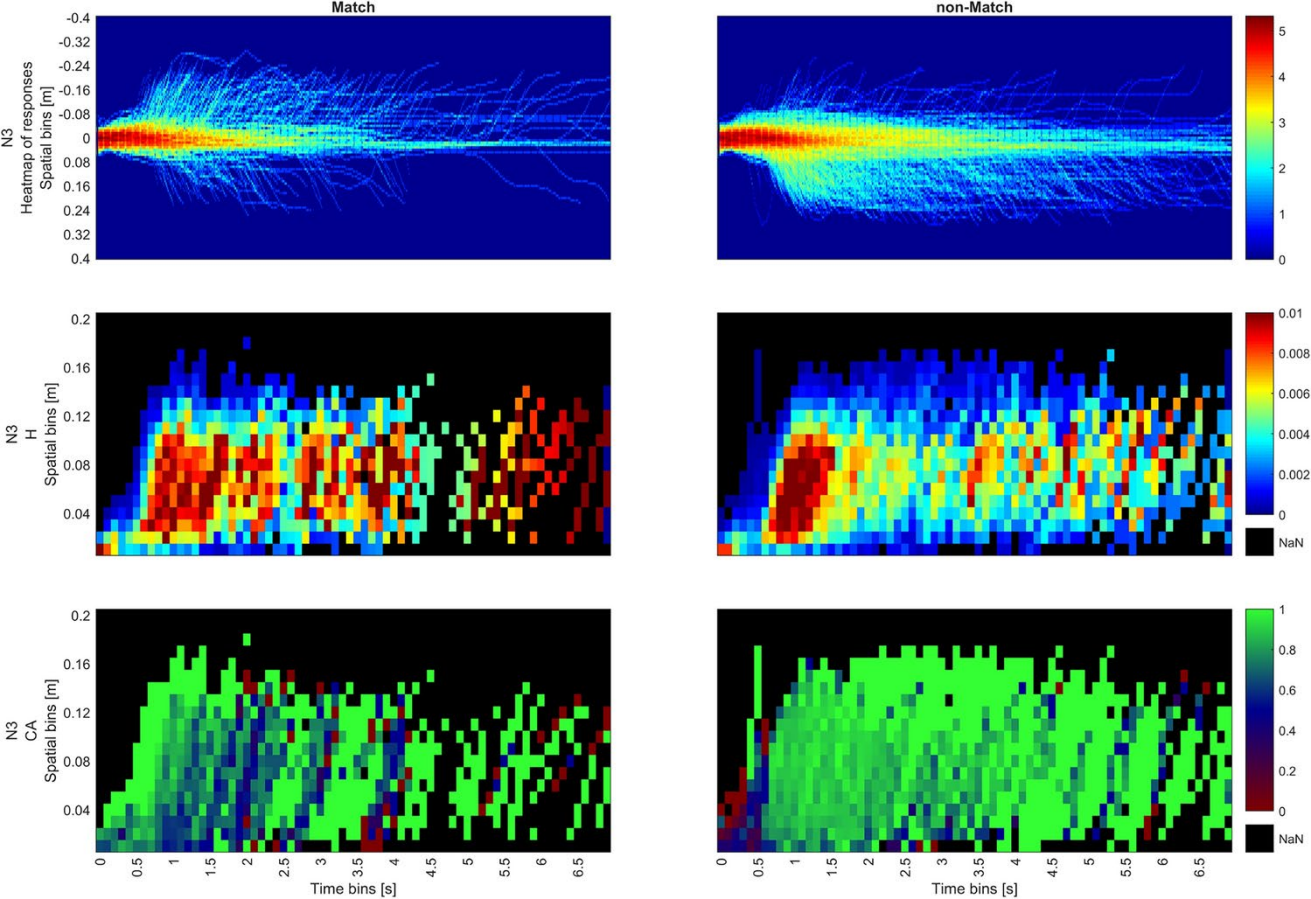
Combined plots for N3 for Match (left) and non-Match conditions (right); Top row show the heat map of responses; middle row shows hazard rates while the bottom row shows conditional accuracy.

Hazard rates drop rapidly after 1500 ms for Match but persist longer for non-Match within the same spatial bins, corresponding to higher RT(VR). Hazard rate exhibits a slight resurgence around 1800 ms in the Match condition, whereas for the non-Match condition, the hazard rate slowly declines with time. This is consistent with the graphs from StSA Stage 2, Fig. 10, where response distributions in the non-Match condition were more widely spread in time. Also, this observation is consistent with the results of the SA which shows the sparsity of responses at the tail end of the distribution in Fig. 5i. Both the persistence at halfway and the late resurgence in the Match condition become more pronounced as N increases. For both N2 and N3, the hazard is highest around the halfway point in the 500-800 ms interval of the trial, declining sharply for the Match condition and gradually waning for the non-Match condition. For the Match condition, the resurgence of the hazard rate for N2 occurs at 2000 ms and for N3 at 2500 ms. This results in two hazard peaks across all Match trials, which can be observed in Figs. 10, 11, 12.

In the Match condition, high hazard is associated with high conditional accuracy. On the other hand, conditional accuracy in the non-Match condition in spatiotemporal bins with high hazard is slightly lower. Conditional accuracy in the bins at the origin (0-400 ms, 0-0.04 m) tends to be highly accurate in the Match condition, but highly inaccurate in the non-Match condition. This shows a strong Match bias is starting from movement initiation. This bias can be observed past 400 ms with increasing N and is overcome only if movements are initiated later in time.

These phenomena are more conspicuous in N2 and N3, where there the initial frequency neglect effect that is evident in conditional accuracy for both conditions (0–400ms). This effect is surprising considering that Match trials occurred only with a probability of 33%, but was previously observed in as frequency neglect in SA. Additionally, accuracy is noticeably high in the later spatial bins (bins close to the end of the trajectory). This implies that as the decision process advances toward its conclusion, movements tend to be more precise.

#### Inferential analysis

We provide an example of hypothesis testing in StSA. Our example concentrates on effects occurring within consecutive time windows. This method does not consider the possible dependencies that may exist across time series. Alternatively, we could select spatial windows from the trajectory data to perform a time series analysis^22^.

**Fig. 13:**
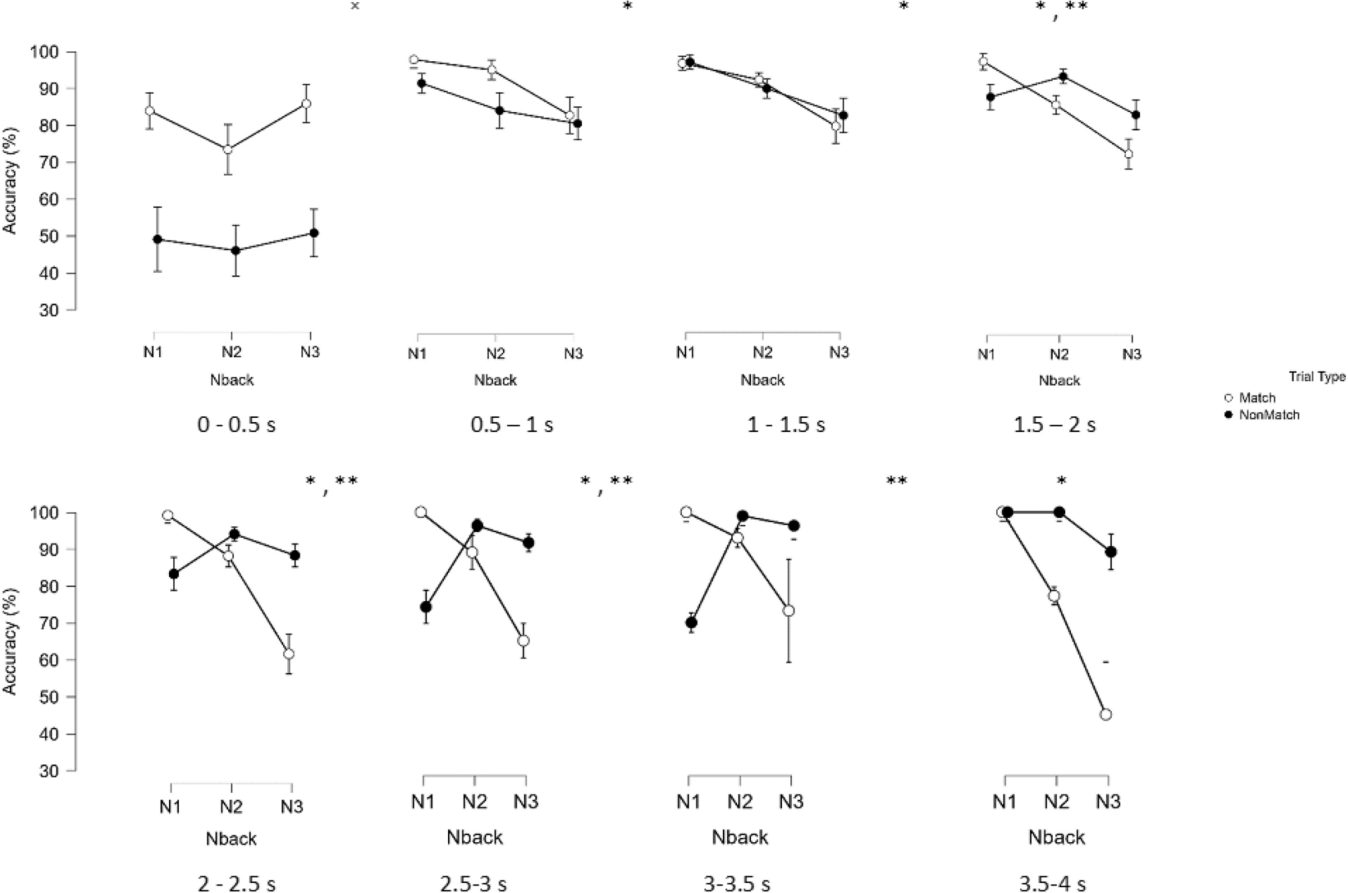
Conditional Accuracy data, for the time windows from 0-4 s, analyzed separately. $\times$ indicates a significant main effect of Trial Type, * indicates a significant main effect of N-back, ** indicates a significant interaction between N-back and Trial Type.

We compared conditional accuracy (*CA*) values for all Match conditions (Match vs. non Match) and N-back conditions (1-3) as an example of an inferential test. Repeated-measures ANOVAs (2x3) were conducted 8 times, once for each in a 0.5 s time window from stimulus onset (between 0-4 s). Each ANOVA used the spatial bins as repeated measures. We used time windows of 0.5 s to ensure enough trials per participant. The values for conditional accuracy are then averaged across spatial bins, for each time window and for each participant and are shown in Fig. 13.

The inferential test distinguishes three intervals according to their effects: Between 0 and 0.5 s we observe no effect of N-back but a large effect of Trial Type. Match trials are much more accurate than non-Match trials across all conditions. This confirms the presence of frequency neglect, as first identified in the SA. Between 0.5-1.5 s, no effects for Trial Type but effects for N-back are observed. Accuracy decreases with Increasing N. Finally, between 1.5-3 s, there is an interaction effect between Trial Type and N-back. Whereas accuracy is higher for Match trials than for non-Match trials in N1, in N2 and N3 this effect is reversed. This is because with increasing task difficulty, participants in the late responses tend to avoid Match responses. This observation is accordance with category frequency-tuned responses for Non-match trials (66%) as opposed to Match trials (33%) in our experiment.

**Fig. 14:**
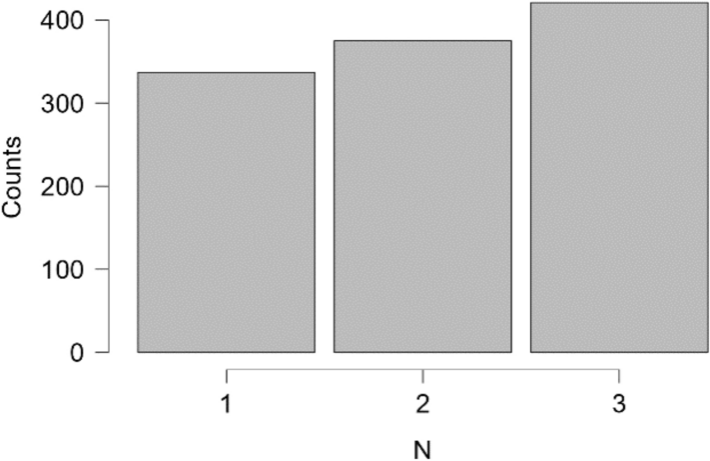
Distribution Plots for trials below 500 ms for each N.

**Fig. 15:**
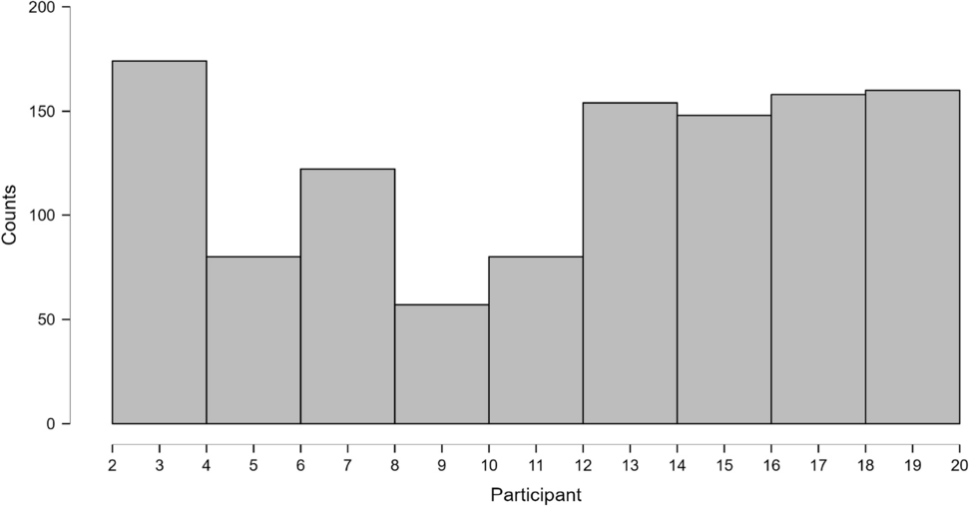
Distribution Plots for trials below 500 ms for each participant.

To evaluate the robustness of our findings, we conducted an analysis to showcase the number of trials in the first 500ms for each participant and condition. We summed the number of unique trials that occur within this time window. Table 1 shows that 1133 trials were included in this analysis (almost one third of the total number of trials). Figures 14 and 15, show that we observe early movements in every participant and condition. The effect is quite strong and systematic, given that the location of the boxes was counterbalanced (it is not merely a left-right bias). The analysis underscores that there is strong evidence for movement towards the Match box in the first 500ms. This effect is not visible in SA, as the earliest completed responses in the VR condition occur after 600ms (as shown in Fig. 5).

**Table 1:** Descriptive Statistics for trials in the first 500ms.

Statistic	N	VP
Valid	1133	1133
Missing	0	0
Mean	2.074	12.062
Std. Deviation	0.815	5.749
Minimum	1.000	2.000
Maximum	3.000	20.000

#### Summary and discussion

The SA had suggested early frequency neglect as well as late effects of hesitation and frequency tuning. However, lack of measurement points prevented us from drawing any conclusions. StSA overcomes this issue by analyzing multiple responses per trial. The frequency neglect effects are observable, first, in the averaged trajectories (<500 ms). They show a small net deviation from the origin towards the Match response box. This deviation occurs in both Match and non-Match trials indiscriminately. Later parts of the response trajectory (500-700 ms) on average reveal the opposite effect, this time differentiated according to the rate with which they move towards the alternative response boxes. These effects are confirmed by the inferential tests.

Single trial plots show end point variability across different trials, which explains the variability in the averaged trajectory data and the high variance demonstrated by the ANOVA. In addition, spatial separation between Match and non-Match trials becomes less conspicuous with higher N values. These effects are characterized by hesitation (hovering in an undecided regions) and self-correction (changing the direction of approach). Single trial plots exhibit these effects, while the numerical values expressed in heat maps quantify them.

## Discussion

Classical experiments have relied primarily on discrete measures such as response times (RT) and accuracy. Often, ANOVAs performed on these data suffer from assumption violations and power problems. We have illustrated this for a classical N-back task and a virtual reality (VR) version. While significant results were consistently obtained, they are difficult to interpret, mainly because the variance increases systematically across conditions. Most importantly, RT and accuracy are discrete snapshots and therefore inadequate windows into the continuous dynamic processes of cognition. To some extent, this is remedied by survival analysis (SA). However, new performance issues arise, especially in the later stages of responses. Using SA on both classical and VR versions of the N-back task, we have been able to make some tentative observations about the course of the response process, which for the moment must remain speculative in the absence of data on response trajectories. In general, trajectories help to increase experimental power by providing more precise and continuous measurements.

We are not the first to attempt to analyze response trajectories. Pointing movement trajectories have provided information about the time required for movement planning^23–25^. These studies conclude that movement planning occurs within 100-150 ms of stimulus onset and that reconsideration takes time. This is consistent with our current observations of delays caused by hesitation and self-correction.

In addition to delays, bottom-up distractors can lead to spatial deviations in trajectories^6,26^. In both studies, the authors report early trajectory deviations toward the distractor that were later redirected toward the target. The initial deviations in trajectory effects are similar to the early errors observed in classical response conditions^27^. Conversely, different effects could also be obtained from top-down processing. Here, Rheem and colleagues showed that in high load conditions, participants responded more slowly but, with less deviation from the optimal path to the target^9^. This result is similar to response inhibition as observed, for instance, in eye move movement trajectories in Go/No-go tasks^28^. These contrasting effects illustrate that trajectory tracking can be diagnostic of the nature of the response mechanism.

For our VR response trajectory tracking data, we offered Spatiotemporal Survival Analysis (StSA) as a novel method that exploits the high spatiotemporal resolution of these data. StSA quantifies their dynamics and variability, and its analyses are theory-free - no curve fitting. Trials are grouped into temporal and spatial segments, revealing otherwise hidden trends. In the N-back task, we observed a strong preference for the rare response category, i.e., frequency neglect in fast responses, leading to nearly 100% accuracy in Match conditions but 0% in Non-match conditions. In addition, we confirmed some observations about hesitation and self-correction in slow responses, such as tuning to response category frequencies. These observations may reinforce our doubts about standard data trimming procedures, which systematically eliminate both very early and late response effects.

In contrast, SA and StSA use all available data for the analysis, right censoring only parts of responses that are too long. Thus, these methods can identify deviations and delays at different times during the trajectory as indicative of a decision process^5,9,26^. An example on how these methods can inform theory comes from Dynamic Field Theory (DFT;^29^). In the N-back task, Match trials precisely correspond to a memory trace established N trials prior. DFT predicts that this trace would generate strong activation patterns in neural systems, facilitating more accuracy in faster responses^30^. Non-match trials, in contrast, lack this direct trace and require inhibitory processes to suppress competing neural activation. Given the noisy fluctuations in neural representations, the absence of a match peak does not necessarily equate to certainty about non-match status, introducing delays and variability. In movement trajectories, this causes early motor responses to be disproportionately biased toward Match classifications, even when they are incorrect. StSA shows both these patterns of response, the early “Match bias” and the delays and variability of non-match responses.

The implications of these findings extend beyond the N-back task/DFT to broader theories of cognition and decision-making. They suggest that decision processes continue to occur during response execution, whereas classically the overlap between decision and response execution is typically assumed to be minimal. This challenges previous models of decision-making, which assume that participants make decisions only after accumulating sufficient information. For example, drift-diffusion models (DDM) represent accumulation as a random walk with drift that continues until one of the alternative decision boundaries is reached, triggering response initiation^31,32^. Our VR results show that instead, decision-making and response execution overlap to a certain degree. They also confirm that early motor responses are shaped not by completed decisions but by the ongoing dynamics of neural competition and coupling between perceptual and motor systems. This type of data can also encourage computational modelling of the perceptual and motor systems.

The parallels observed between the VR and classical versions of the N-back task suggest that the same processes are involved in both. Two of the limitations of the N-back task, and WM tasks in general, are the complexity of the sub-processes and the large individual differences within populations^33–35^. These complexities make many analyses of averaged data inappropriate, as they rely on extensive data normalization and averaging across conditions and participants^17^. Not only ANOVAs, but also previous analyses of movement trajectory data have relied heavily on these data processing techniques^20,36^ . Instead, distributional analyses, such as SA and StSA may be preferred.

**Fig. 16:**
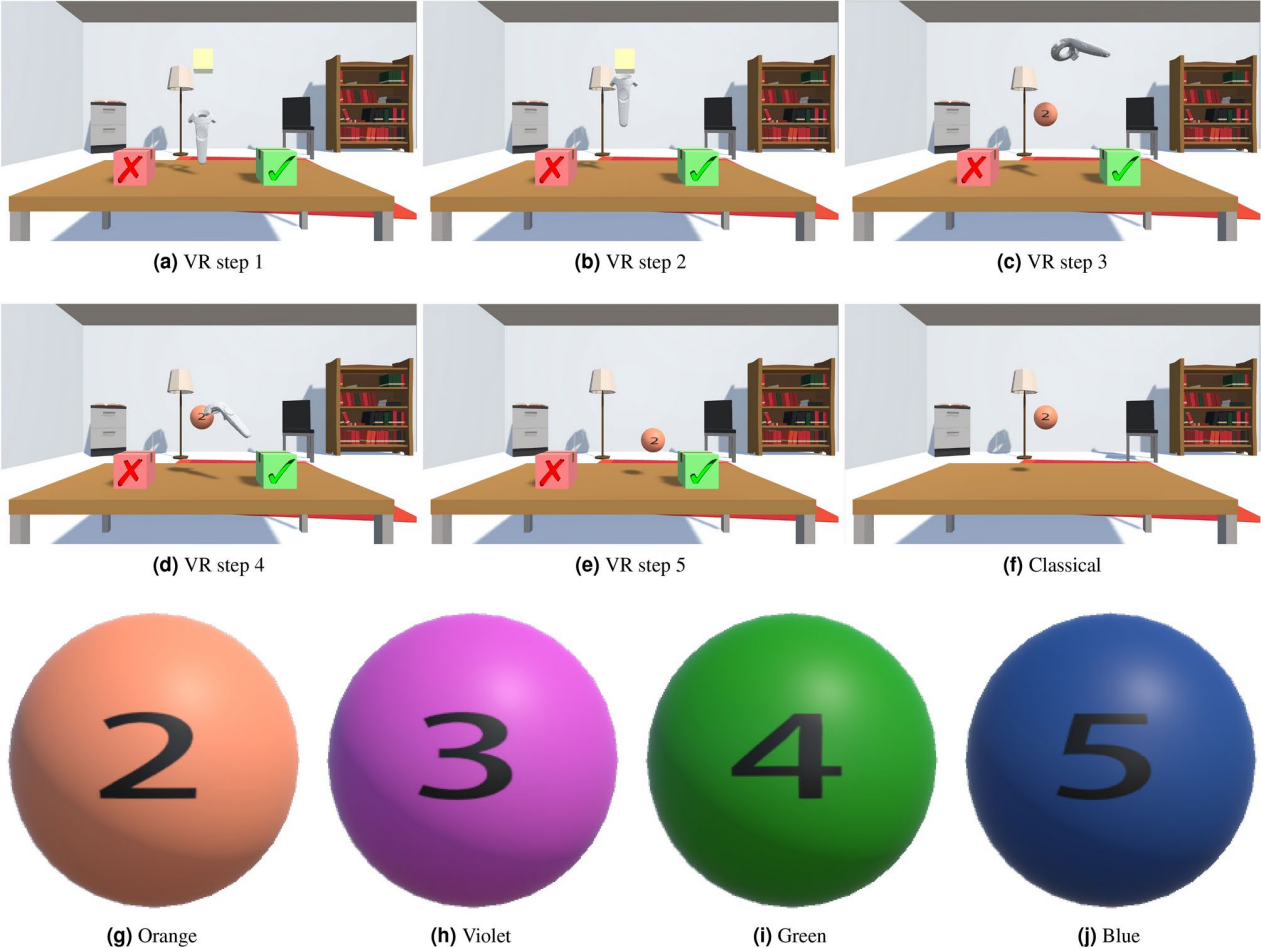
Upper half: Visual displays of both classical and VR versions of the N-back experiment. The classical version shows the stimulus in a static environment. In the VR version, a fixation box and two response boxes are additionally shown, as well as a controller in five steps in the dynamical VR display. Step 1 is pre-stimulus presentation where participants only see the yellow box and the response boxes. In steps 2 and 3 participants move into the fixation box, prompting the stimulus to appear. In Step 4, the participant moves towards the stimulus, capturing and controlling it in step 5. In the lower half, the four different stimuli are displayed.

Rather than a comprehensive exploration of visual working memory, our goal was to use a VWM task to introduce StSA. We limited our investigations to contrasting response strategies in different time windows, ignoring for the moment the possibility of performing time series analyses on these data^22^. We did not consider individual differences or the effects of learning on task performance. In this respect, an interesting question is when the observed response category frequency effects occur during the course of the experiment. Another possible limitation relates to our experimental design. We used a “static” starting procedure (see Fig. 16, VR Step 1). In mouse tracking studies, static start procedures generally slow down movement initiation. However, our current experimental setup may have prevented this, as holding the hand with the controller in a raised position would cause some discomfort. Indeed, this may be one of the reasons for the observed frequency neglect.

In summary, response trajectories hold great potential for understanding dynamic decision-making and cognitive processing in general. StSA analyses offer a versatile, model-free tool for analyzing these data, suitable for both exploratory data analysis and hypothesis testing in a variety of research domains.

## Methods

### Participants

Twenty right-handed university students (6 males, ages 20-30 years, mean age of 26.3 years, SD = 2.5 years) from the German city of Kaiserslautern took part in the study. They all had normal or corrected-to-normal vision. According to self-reports, participants had no impairment of speech and reading as well as no diagnosis of psychological or neurological disorders. They all gave their informed written consent before participation. The experiment was approved by the ethics committee of the Faculty of Social Sciences at the University of Kaiserslautern, as according to the ethical standards of the institution and with the 1964 Helsinki Declaration.

#### Stimuli

We used the display of Fig. 16 (upper half). In the classical version, the display was a static image, lacking the yellow fixation box or the two response boxes. In the VR version, the display constituted a dynamic, 3-dimensional environment. Displayed objects cast shadows, considered important to enhance the sense of immersion in a virtual environment^37^.

Numbers ranging from 2-5 were the target stimuli in both the classical and the VR versions of the experiment. They appeared as labels of 4 different colored balls, Orange, Violet, Green, and Blue, as shown in the lower half of Fig. 16. Colors and numbers were always matched, e.g., the blue ball always had the number 5. In both classical and VR versions, targets were placed on top of a virtual desk.

#### Apparatus

The study was conducted in a dimly lit room. Tasks were executed on a high-end gaming laptop computer running a Windows 10 Enterprise 2016 LTSB 64-bit operating system with an Intel Core i7-8750H processor. Stimuli in the classical version of the experiment were presented on a monitor (40cm x 30cm) of a PC with a screen resolution of 1920 x 1200 and a refresh rate of 90 Hz, running g-sync technology to ensure a stable frame rate. Participants were seated 70 cm away from the monitor. The VR version used an HTC Vive pro-VR headset with 1080x1200 per eye resolution and a refresh rate of 90Hz. The experiment was programmed using Unity Software, version 2020.1.17f1. In VR, hand movement trajectories were recorded throughout trials using VIVE Pro controllers.

#### Design and procedure

The classical and VR versions consisted of equivalent sequences of events as follows: In each trial, a randomly selected stimulus was presented and participants responded as quickly and accurately as possible whether the current stimulus was an identical Match to the stimulus presented N trials earlier. Each trial had an apriori probability of 33% being a Match. The value of N increased from 1 to 4 after every second block. Participants completed two sessions in counterbalanced order, one on a PC (the classical version) and one in VR. Each session lasted approximately one hour and consisted of seven experimental blocks of 40 trials each. A session began with a practice block of 20 trials, also with a 33% chance of being a Match, in which participants indicated whether the target stimulus was a green ball. Sessions were administered on different days, on average about 1 week apart.

In the classical version, a trial began with the appearance of the display with one of the target stimuli presented in the center of the screen until the response, in which participants pressed one of two response keys (letters F and J on a keyboard) with the left or right index finger to indicate Match or Non-match, respectively. Key assignment was fixed for each participant and counterbalanced across participants. The stimulus disappeared immediately after a key was pressed, and the next trial began after a one second (s) interval.

**Fig. 17:**
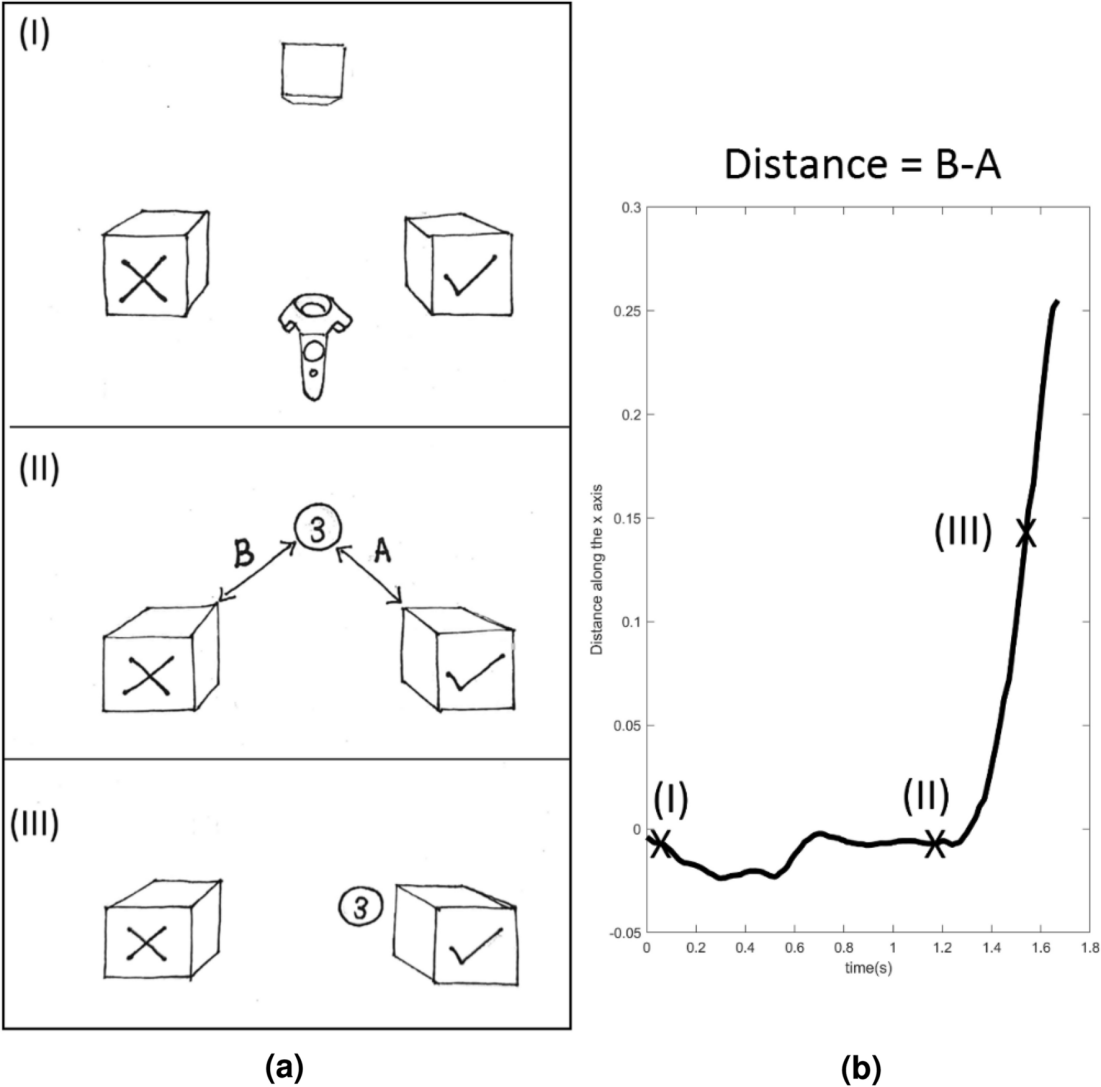
Response procedure in the VR version of the N-back experiment (**a**). In stage (I), a transparent box appears, and the controller starts in a neutral position between the two response boxes, the checkmark indicates “Match”, and the cross indicates “non-Match”. In stage (II) the stimulus is captured; A and B represent the distances between the stimulus and the two boxes respectively. In (III) the stimulus is moved closer to one of the boxes. Stages (I, II, and III) of the response procedure depicted as a function of the stimulus position with respect to the response boxes (**b**). The movement trajectory is calculated by subtracting the distance between the two response boxes (B-A).

For the VR version, the procedure is shown in Fig. 17. Participants began each trial with a transparent box that was presented in the center of the scene until the participant moved the controller inside the box. The box then immediately disappeared and the target stimulus appeared halfway between the transparent box and the table (see top half of Fig. 16), and movement trajectory tracking began. Participants moved the controller toward the stimulus and then pressed and held the trigger button to capture it. Once the stimulus was captured, the controller became invisible and participants had to hold down the trigger button to maintain control of the stimulus. Using the now invisible controller, they moved the stimulus in three-dimensional virtual space to the green box if the target matched, and to the red box otherwise. The target stimulus disappeared when it collided with one of the boxes, and the controller reappeared instead. If the trigger button was released before the collision, the stimulus remained in the environment and the controller reappeared. Thus, participants could move their hands to a resting position at any time during or after a trial. The positions of the boxes were counterbalanced across participants to avoid any bias due to direction or the presence of shadows (left-right bias). No restrictions were imposed on the movements participants could make. The trial ended when the ball reached the borders of one of the two boxes. After a one second interval, the transparent box reappeared. The next trial began immediately after participants moved the controller back into the transparent box.

#### Analyses

While all participants completed the task up to the three-back task (N3), 5 participants did not complete the four-back task (N4), neither in the classical nor the VR version due to task difficulty or fatigue. Therefore, this condition was excluded from further analyses. Of the 20 participants, one was also excluded due to data corruption. For the remaining 19 participants, RT were collected for the classical version and movement trajectories for the VR version. We began our analysis with response time and accuracy measures for the classical and VR versions of the task, i.e., RT(C), RT(VR), accuracy(C), and accuracy(VR), respectively. Accuracy(C) and accuracy(VR) are determined by the correctness of the responses. The analog of RT(C) in VR, RT(VR), represents the time taken to complete the response. This was specified as the time from the moment the controller entered the fixation box until the stimulus collided with one of the boxes. Note that this interval includes the time participants needed to capture the stimulus, as well as periods when participants released the stimulus before arrival, causing it to remain stationary. In a recent study, accuracy(VR) and RT(VR) reliably reproduced the classical effects^38^. For the N-back task, we expect that, as task difficulty increases (i.e., higher N), accuracy(VR) decreases while RT(VR) increases, as in their classical counterparts^17^.

For explanatory purposes, we first compared results between classical and VR versions using traditional analyses of variance (ANOVA). The use of ANOVA raises issues of adequacy of assumptions, such as homogeneity of variance^39^ and sphericity^40^, and statistical power^41^. Violations of the assumptions threaten the robustness of the data^42^. Statistical power refers to the ability of an experiment to detect an effect in a sample distribution^43^. Known determinants are the number of participants and the number of trials per participant and condition. Interactions in ANOVA tend to be modest in size, making it difficult to achieve adequate power. In addition, conducting an appropriate power analysis depends on the shape of the interaction, which is a determinant of the effect size^44^. Many of the factors that affect power in multifactor repeated measures designs are unknown. This further complicates statistical power calculations. Measurement precision at the individual participant level should be considered. This could be achieved by increasing the number of trials per condition and the number of measurements per trial^45^.

Another way to increase power is to raise measurement precision (as defined by Eisenhart, 1963). Movement trajectories achieve this by having more internal validity than simple reaction times. This internal validity is a result of multiple recordings per trial, as well as the dependence of each recording on the previous one. For example, instead of recording a single reaction time, one could record the initiation of the movement and the subsequent alternations to the end of the trajectory. As is common in ANOVA, the data were trimmed for outliers using interquartile outlier rejection (1.5 interquartile range;^46^) and further trimmed to exclude values outside the range of the mean plus two standard deviations. Trimming eliminates data considered less representative of the underlying population. It reduces the variance in the sample to meet the criteria for planned analyses. However, it runs the risk of underestimating population means and variances. In addition, trimming may eliminate important aspects of response dynamics and thus be tantamount to throwing the baby out with the bathwater. Variances typically increase with RT, and plausibly with RT(VR), as a result of hesitation or self-correction, which may be of interest in understanding responses.

Despite the trimming procedure, we still encountered unequal variances and violations of the sphericity assumption. To address these violations in the usual way, we simply ignored the former and applied the Greenhouse-Geisser correction^40^ for the latter, when necessary. The Supplementary Material provides an ANOVA on the untrimmed data that shows the full extent of the disparity in variances. Subsequently, we conducted distributional analyses without data trimming. Distributional analyses of response latency measures across trials have become more relevant in recent years^18,27,47–49^. Such analyses enhance our comprehension of the temporal characteristics of data beyond the insights provided by mean RT analysis. Survival Analysis (SA) is one of the most diagnostic approaches^18^ as well as a standard tool in many scientific disciplines when analyzing time-to-event data^50,51^. It provides insights into the temporal dynamics of response activation, hesitation, and indecision.

SA investigates the time course of events, delineated by RTs and ERs for classical, alongside their respective counterparts in the VR version. In the first stage of the analysis, time is divided into discrete time bins t. The hazard function $( \textit{h} \mathrm {(t) = P(T = t \mid T \ge t)}$) plots hazard, which is the conditional probability of a response occurring in a time bin given that the response had not previously occurred, against the passage of discrete time. Where T represents the time to a response. The survivor function $( \textit{S} \mathrm {(t) = P(T > t) = 1 - P(T \le t)}$) provides the probability that a response does not occur before the end of bin t. In other words, the percentage of the total trials without a response by the end of that bin. Using linear interpolation, median RT can be estimated, which is equivalent to *S*(t)=.50. The conditional accuracy function $( \textit{ca} \mathrm {(t) = P(\text {correct} \mid T = t) }$) plots the conditional probability of a correct response in bin t against the passage of discrete time^52,53^. SA remedies the trimming problems of ANOVA but increases the problem of power. Near the tail end of the distribution where estimates are based on a vanishing small amount of data. This is not necessarily a limitation of SA, but rather the data generated with the N-back task. This task generally has large inter-individual differences, and large differences between conditions. Thus, any inference made using SA must be interpreted cautiously; since there is little data to make definitive claims, only indications of potential patterns.

The problems in ANOVA designs arise from relying on impoverished data^1^. Accounting for the number of observations per participant in the statistical power calculations^43,54^ allows for small-n designs, which are designs with a small number of participants^55^. Small-n designs allow for the replication of data at the individual participant level, rather than through averaging across participants^55^. Movement trajectories provide a large number of observations per trial, allowing for small-n designs. Another factor with the same effect is within-trial measurement precision^56^. Movement trajectories offer potentially high precision because the spatiotemporally resolved nature of the trajectory data allows for the extraction of multiple data points from each trial. Trajectory data can be analyzed as a series, transitions can be identified, and segments can be assigned to distinct stages such as movement initiation, subsequent redirection, and trajectory completion^3–5^.

There are several approaches to observing trajectories. For example, Scherbaum and Dshemuchadse (2020) present time-continuous multiple regression analysis (TCMR; Scherbaum et al., 2013). TCMR extracts individual markers from trajectories using either beta-weights tested across participants or Gaussian curve fitting. This approach requires outlier rejection and extensive data trimming, as errors strongly bias the extracted measures. In addition, it is difficult to do this for individual participants. Furthermore, according to the authors, a different approach would be needed for complex stimuli or experimental designs with variable delays in movement initiation. March and Gaertner (2021) use trajectories to determine when categorization occurs, using the Time of Initiating Correct Categorization (TICC;^36^). Although TICC can be calculated from individual trials, the authors recommend using averaged trajectories instead due to the noisy nature of mouse trial data. Both approaches artificially encourage mouse movement, either by forcing participants to move before stimulus presentation or by enforcing a response deadline. This limits what can be inferred from the task (see Karşılar et al., 2014).

These approaches are also selective for certain elements in the data that are considered theoretically significant (e.g., TICC). The theory-laden nature of these measures reduces their usefulness for cross-theory comparisons. For this reason, we developed an approach to trajectories that exploits the full richness of the data and is theory-free - no curve fitting. We call this approach Spatiotemporal Survival Analysis, or StSA. Using StSA allows us to study the time course of responses as they occur naturally, with no constraints on movement initiation or execution other than physical ones (e.g., gravity). StSA segments trajectories into many small steps, allowing the identification of multiple events throughout a trial. The StSA pipeline produces descriptive plots, trajectory distributions, and time-to-event data that contain details that are easy to interpret. Inferential methods such as ANOVA can then be used at each time point of the trajectory, analogous to evoked potentials, although methods such as bootstrapping or jackknifing^57^ are also possible.

StSA consists of several stages. In Stage 0, an optional preliminary stage, average movement trajectories are computed to obtain a course overview of each condition. In the first stage, single trials are visualized for all participants in each condition. Note that we do not perform any trimming of the data for the StSA. In the second step, the raw data are visualized by dividing time and space into discrete spatiotemporal bins. Each bin counts the number of trajectories that visit a given location in a given time. These counts are presented in a high-resolution heat map of the responses, showing the frequency distribution of trajectories visiting each spatiotemporal bin.

In the third and final stage of the analysis, StSA is performed using predefined spatiotemporal bins. In this stage, we identify events as the first instance where the absolute value of a trajectory exceeds a spatial threshold, and then use its direction of travel (toward the left or right box) to determine accuracy. Each trial is divided into 20 measurements; each measurement represents the first time a trajectory crosses a spatial threshold in either direction (each with 5% of the total distance, incrementally increasing from 0). The direction of crossing (accuracy) and time are collected at each measurement and stored in a spatiotemporal bin labeled by the combination of the discrete time recorded and the spatial threshold crossed. These measures are then used to calculate hazard rates (*H*) and conditional accuracy (*CA*) values in each spatiotemporal bin. *CA*in this case considers the average accuracy of all trials in a given spatiotemporal bin.*H*is defined as the conditional probability of an event occurring within a spatiotemporal bin, given that it has not previously occurred in time. The results for both are presented in heat maps, where higher hazard rates indicate a higher probability that a trajectory will visit a given spatiotemporal bin. These heat maps are then scaled according to the maximum hazard across all conditions. Similarly, conditional accuracy is also represented in heat maps. StSA, including the visualization of *H *and *CA *in plots, could allow inferences about the fine-grained dynamics of individual differences in cognitive tasks.

#### Analysis software

ANOVAs were carried out using JASP (0.17.1); discrete SA was done using Rstudio (2023.03.1, running R version 4.0.2), StSA trajectory analyses and visualizations were carried out using MATLAB (Version: 9.4.0.813654 (R2018a)).

## Supplementary Information

Supplementary Information.

## Data Availability

The datasets generated and analyzed for the current study, as well as the code are available in the OSF repository, osf.io/anekb. DOI 10.17605/OSF.IO/ANEKB Prior versions: A version of this manuscript was uploaded to PsyArXiv as a Preprint: https://doi.org/10.31234/osf.io/6spkw

## References

[1] Spivey, M. J. & Dale, R. Continuous dynamics in real-time cognition. *Curr. Dir. Psychol. Sci.* **15**, 207–211. 10.1111/j.1467-8721.2006.00437.x (2006).

[2] Kieslich, P. J., Schoemann, M., Grage, T., Hepp, J. & Scherbaum, S. Design factors in mouse-tracking: What makes a difference?. *Behav. Res. Methods* **52**, 317–341. 10.3758/s13428-019-01228-y (2020). 30963463 10.3758/s13428-019-01228-y

[3] Woodworth, R. S. The accuracy of voluntary movement. *J. Nerv. Ment. Dis.* **26**, 743–752. 10.1097/00005053-189912000-00005 (1899).

[4] Elliott, D., Helsen, W. & Chua, R. A century later: Woodworth’s (1899) two-component model of goal-directed aiming. *Psychol. Bull.* **127**, 342–357. 10.1037/0033-2909.127.3.342 (2001). 11393300 10.1037/0033-2909.127.3.342

[5] Schmidt, F. & Schmidt, T. Feature-based attention to unconscious shapes and colors. *Attent. Percept. Psychophys.* **72**, 1480–1494. 10.3758/app.72.6.1480 (2010). 10.3758/APP.72.6.1480 20675795

[6] Schmidt, T. The finger in flight: Real-Time motor control by visually masked color stimuli. *Psychol. Sci.* **13**, 112–118. 10.1111/1467-9280.00421 (2002). 11933993 10.1111/1467-9280.00421

[7] Spivey, M. J., Grosjean, M. & Knoblich, G. Continuous attraction toward phonological competitors. *Proc. Natl. Acad. Sci.* **102**, 10393–10398. 10.1073/pnas.0503903102 (2005). 15985550 10.1073/pnas.0503903102 PMC1177386

[8] Freeman, J. B. & Dale, R. Assessing bimodality to detect the presence of a dual cognitive process. *Behav. Res. Methods* **45**, 83–97. 10.3758/s13428-012-0225-x (2013). 22806703 10.3758/s13428-012-0225-x

[9] Rheem, H., Verma, V. & Becker, D. V. Use of mouse-tracking method to measure cognitive load. *Proc. Hum. Factors Ergonom. Soc. Annu. Meet.* **62**, 1982–1986. 10.1177/1541931218621449 (2018).

[10] Niu, H., van Leeuwen, C., Hao, J., Wang, G. & Lachmann, T. Multimodal natural human-computer interfaces for computer-aided design: A review paper. *Appl. Sci.* **12**, 6510. 10.3390/app12136510 (2022).

[11] Schoemann, M., Lüken, M., Grage, T., Kieslich, P. J. & Scherbaum, S. Validating mouse-tracking: How design factors influence action dynamics in intertemporal decision making. *Behav. Res. Methods* **51**, 2356–2377. 10.3758/s13428-018-1179-4 (2019). 30684228 10.3758/s13428-018-1179-4

[12] Banks, M. S., Hoffman, D. M., Kim, J. & Wetzstein, G. 3D displays. *Annu. Rev. Vis. Sci.* **2**, 397–435. 10.1146/annurev-vision-082114-035800 (2016). 28532351 10.1146/annurev-vision-082114-035800

[13] Baddeley, A. Working memory. *Science* **255**, 556–559. 10.1126/science.1736359 (1992). 1736359 10.1126/science.1736359

[14] Conway, A. R. A. et al. Working memory span tasks: A methodological review and user’s guide. *Psychonom. Bull. Rev.* **12**, 769–786. 10.3758/bf03196772 (2005). 10.3758/bf03196772 16523997

[15] Kirchner, W. K. Age differences in short-term retention of rapidly changing information. *J. Exp. Psychol.* **55**, 352–358. 10.1037/h0043688 (1958). 13539317 10.1037/h0043688

[16] Wheeler, M. E. & Treisman, A. Binding in short-term visual memory. *J. Exp. Psychol. Gen.* **131**, 48–64. 10.1037/0096-3445.131.1.48 (2002). 11900102 10.1037//0096-3445.131.1.48

[17] Meule, A. Reporting and interpreting working memory performance in n-back tasks. *Front. Psychol.*10.3389/fpsyg.2017.00352 (2017). 28326058 10.3389/fpsyg.2017.00352 PMC5339218

[18] Panis, S., Schmidt, F., Wolkersdorfer, M. P. & Schmidt, T. Analyzing response times and other types of Time-to-Event data using event history analysis: A tool for mental chronometry and cognitive psychophysiology. *i-Perception* **11**, 204166952097867. 10.1177/2041669520978673 (2020). 10.1177/2041669520978673 PMC8822313 35145613

[19] Sunstein, C. R. Probability neglect: Emotions, worst cases, and law. *Yale Law J.* **112**, 61. 10.2307/1562234 (2002).

[20] Scherbaum, S. & Dshemuchadse, M. Psychometrics of the continuous mind: Measuring cognitive sub-processes via mouse tracking. *Memory Cognit.* **48**, 436–454. 10.3758/s13421-019-00981-x (2020). 10.3758/s13421-019-00981-x 31721062

[21] Hedne, M. R., Norman, E. & Metcalfe, J. Intuitive feelings of warmth and confidence in insight and noninsight problem solving of magic tricks. *Front. Psychol.*10.3389/fpsyg.2016.01314 (2016). 27630598 10.3389/fpsyg.2016.01314 PMC5005374

[22] Solfo, A. & van Leeuwen, C. A Bayesian classifier for fractal characterization of short behavioral series. *Psychol. Methods*10.1037/met0000562 (2023). 37126040 10.1037/met0000562

[23] Brenner, E. & Smeets, J. B. J. Intercepting moving targets: Why the hand’s path depends on the target’s velocity. *Proc. SPIE*10.1117/12.610849 (2005).

[24] Day, B. L. & Lyon, I. N. Voluntary modification of automatic arm movements evoked by motion of a visual target. *Exp. Brain Res.* **130**, 159–168. 10.1007/s002219900218 (2000). 10672469 10.1007/s002219900218

[25] Paulignan, Y., Jeannerod, M., MacKenzie, C. L. & Marteniuk, R. G. Selective perturbation of visual input during prehension movements. *Exp. Brain Res.*10.1007/bf00229827 (1991). 1769391 10.1007/BF00231858

[26] Cressman, E. K., Franks, I. M., Enns, J. T. & Chua, R. On-line control of pointing is modified by unseen visual shapes. *Conscious. Cogn.* **16**, 265–275. 10.1016/j.concog.2006.06.003 (2007). 16854595 10.1016/j.concog.2006.06.003

[27] Wolkersdorfer, M. P., Panis, S. & Schmidt, T. Temporal dynamics of sequential motor activation in a dual-prime paradigm: Insights from conditional accuracy and hazard functions. *Attent. Percept. Psychophys.* **82**, 2581–2602. 10.3758/s13414-020-02010-5 (2020). 10.3758/s13414-020-02010-5 PMC7343743 32166642

[28] Camalier, C. R. et al. Dynamics of saccade target selection: Race model analysis of double step and search step saccade production in human and macaque. *Vision. Res.* **47**, 2187–2211. 10.1016/j.visres.2007.04.021 (2007). 17604806 10.1016/j.visres.2007.04.021 PMC2041801

[29] Schöner, G., Spencer, J. & Group, D. R. *Dynamic thinking* (2015).

[30] Spencer, J. P., Perone, S. & Johnson, J. S. *Dynamic field theory and embodied cognitive dynamics* (2009).

[31] Ratcliff, R. A theory of memory retrieval. *Psychol. Rev.* **85**, 59–108. 10.1037/0033-295x.85.2.59 (1978).

[32] Ratcliff, R., Smith, P. L., Brown, S. & McKoon, G. Diffusion Decision Model: Current issues and history. *Trends Cogn. Sci.* **20**, 260–281. 10.1016/j.tics.2016.01.007 (2016). 26952739 10.1016/j.tics.2016.01.007 PMC4928591

[33] Chen, Y.-N., Mitra, S. & Schlaghecken, F. Sub-processes of working memory in the N-back task: An investigation using ERPs. *Clin. Neurophysiol.* **119**, 1546–1559. 10.1016/j.clinph.2008.03.003 (2008). 18448388 10.1016/j.clinph.2008.03.003

[34] Kane, M. J., Conway, A. R. A., Miura, T. K. & Colflesh, G. J. H. Working memory, attention control, and the n-back task: A question of construct validity. *J. Exp. Psychol. Learn. Mem. Cogn.* **33**, 615–622. 10.1037/0278-7393.33.3.615 (2007). 17470009 10.1037/0278-7393.33.3.615

[35] Lamichhane, B., Westbrook, A., Cole, M. W. & Braver, T. S. Exploring brain-behavior relationships in the N-back task. *Neuroimage* **212**, 116683. 10.1016/j.neuroimage.2020.116683 (2020). 32114149 10.1016/j.neuroimage.2020.116683 PMC7781187

[36] March, D. S. & Gaertner, L. A method for estimating the time of initiating correct categorization in mouse-tracking. *Behav. Res. Methods* **53**, 2439–2449. 10.3758/s13428-021-01575-9 (2021). 33846966 10.3758/s13428-021-01575-9

[37] Steinicke, F., Hinrichs, K. & Ropinski, T. Virtual reflections and virtual shadows in mixed reality environments. In *Human-Computer Interaction - INTERACT 2005* (eds Costabile, M. F. & Paternò, F.) 1018–1021 (Springer, 2005).

[38] Jubran, O. F., Rocabado, F., Muntini, L., DuñAbeitia, J. A. & Lachmann, T. Reproducing Classical Priming, Flanker, and Lexical Decision Tasks in VR: Between Ecological Validity and Experimental Control. In *Proc. 33rd European Conference on Cognitive Ergonomics*, 1–5 (2022).

[39] Brown, M. B. & Forsythe, A. B. Robust Tests for the Equality of Variances. *J. Am. Stat. Assoc.* **69**, 364–367. 10.1080/01621459.1974.10482955 (1974).

[40] Greenhouse, S. W. & Geisser, S. On methods in the analysis of profile data. *Psychometrika* **24**, 95–112. 10.1007/bf02289823 (1959).

[41] Bishop, D. Rein in the four horsemen of irreproducibility. *Nature* **568**, 435. 10.1038/d41586-019-01307-2 (2019). 31019328 10.1038/d41586-019-01307-2

[42] Field, A. P. & Wilcox, R. R. Robust statistical methods: A primer for clinical psychology and experimental psychopathology researchers. *Behav. Res. Ther.* **98**, 19–38. 10.1016/j.brat.2017.05.013 (2017). 28577757 10.1016/j.brat.2017.05.013

[43] Baker, D. H. et al. Power contours: Optimising sample size and precision in experimental psychology and human neuroscience. *Psychol. Methods* **26**, 295–314. 10.1037/met0000337 (2021). 32673043 10.1037/met0000337 PMC8329985

[44] Sommet, N., Weissman, D. L., Cheutin, N. & Elliot, A. J. How many participants do I need to test an interaction? conducting an appropriate power analysis and achieving sufficient power to detect an interaction. *Adv. Methods Pract. Psychol. Sci.* **6**, 25152459231178730. 10.1177/25152459231178728 (2023).

[45] Biafora, M. & Schmidt, T. Induced dissociations: Opposite time courses of priming and masking induced by custom-made mask-contrast functions. *Attent. Percept. Psychophys.* **82**, 1333–1354. 10.3758/s13414-019-01822-4 (2020). 10.3758/s13414-019-01822-4 31338826

[46] David, F. N. & Tukey, J. W. Exploratory data analysis. *Biometrics* **33**, 768. 10.2307/2529486 (1977).

[47] Panis, S. & Hermens, F. Time course of spatial contextual interference: event history analyses of simultaneous masking by nonoverlapping patterns. *J. Exp. Psychol. Hum. Percept. Perform.* **40**, 129–144 (2014). 23713795 10.1037/a0032949

[48] Proctor, R. W., Miles, J. & Baroni, G. Reaction time distribution analysis of spatial correspondence effects. *Psychon. Bull. Rev.* **18**, 242–266. 10.3758/s13423-011-0053-5 (2011). 21327376 10.3758/s13423-011-0053-5

[49] Ridderinkhof, K. R. Micro- and macro-adjustments of task set: Activation and suppression in conflict tasks. *Psychol. Res.* **66**, 312–323. 10.1007/s00426-002-0104-7 (2002). 12466928 10.1007/s00426-002-0104-7

[50] Allison, P. D. Discrete-time methods for the analysis of event histories. *Sociol. Methodol.* **13**, 61. 10.2307/270718 (1982).

[51] Singer, J. D. & Willett, J. B. Survival analysis. *Handbook of Psychology* 555–580, 10.1002/0471264385.wei0222 (2003).

[52] Pachella, R. G. *The Interpretation of Reaction Time in Information-Processing Research 1* (2021).

[53] Wickelgren, W. A. Speed-accuracy tradeoff and information processing dynamics. *Acta Physiol. (Oxf)* **41**, 67–85. 10.1016/0001-6918(77)90012-9 (1977).

[54] Murphy, K. R., Myors, B., Murphy, K. & Wolach, A. *Statistical Power Analysis* 2nd edn. (Routledge, 2004).

[55] Smith, P. L. & Little, D. R. Small is beautiful: In defense of the small-N design. *Psychon. Bull. Rev.* **25**, 2083–2101. 10.3758/s13423-018-1451-8 (2018). 29557067 10.3758/s13423-018-1451-8 PMC6267527

[56] Eisenhart, C. Realistic evaluation of the precision and accuracy of instrument calibration systems. *J. Res. Natl. Bureau Stand. Sect. C Eng. Instrum.* **67C**, 161. 10.6028/jres.067c.015 (1963).

[57] Miller, J., Patterson, T. & Ulrich, R. Jackknife-based method for measuring LRP onset latency differences. *Psychophysiology* **35**, 99–115. 10.1111/1469-8986.3510099 (1998). 9499711

