# Supplementary material for: Spatiotemporal survival analysis for movement trajectory tracking in virtual reality
Source: Sci Rep. 2025 Mar 1;15:7313. doi: 10.1038/s41598-025-91471-5 (PMC11873313; doi:10.1038/s41598-025-91471-5)
Supplement: Supplementary file 1 — Supplementary Information. [file 41598_2025_91471_MOESM1_ESM.pdf]

Part 1: ANOVA results without trimming

Classical correct RT vs VR Arrival correct times

Within Subjects Effects

	Sphericity correction	Sum of Squares	df	Mean Square	F	p	η²	η²ₚ
N	None	91.506 [a]	2.000 [a]	45.753 [a]	29.611 [a]	< .001 [a]	0.457	0.622
	Greenhouse-Geisser	91.506	1.136	80.557	29.611	< .001	0.457	0.622
Residuals	None	55.626	36.000	1.545				
	Greenhouse-Geisser	55.626	20.446	2.721				
Device	None	0.331	1.000	0.331	0.791	0.385	0.002	0.042
Residuals	None	7.520	18.000	0.418				
TrialType	None	6.662	1.000	6.662	16.164	< .001	0.033	0.473
Residuals	None	7.418	18.000	0.412				
N ✻ Device	None	4.309 [a]	2.000 [a]	2.155 [a]	6.789 [a]	0.003 [a]	0.022	0.274
	Greenhouse-Geisser	4.309	1.475	2.921	6.789	0.008	0.022	0.274
Residuals	None	11.426	36.000	0.317				
	Greenhouse-Geisser	11.426	26.558	0.430				
N ✻ TrialType	None	1.902	2.000	0.951	6.033	0.005	0.009	0.251
	Greenhouse-Geisser	1.902	1.546	1.231	6.033	0.011	0.009	0.251
Residuals	None	5.675	36.000	0.158				
	Greenhouse-Geisser	5.675	27.824	0.204				
Device ✻ TrialType	None	0.451	1.000	0.451	3.348	0.084	0.002	0.157
Residuals	None	2.425	18.000	0.135				
N ✻ Device ✻ TrialType	None	0.031	2.000	0.015	0.110	0.896	1.532×10^{-4}	0.006
	Greenhouse-Geisser	0.031	1.661	0.018	0.110	0.861	1.532×10^{-4}	0.006
Residuals	None	5.025	36.000	0.140				
	Greenhouse-Geisser	5.025	29.889	0.168				

Note. Sphericity corrections not available for factors with 2 levels.

Note. Type III Sum of Squares

[a] Mauchly's test of sphericity indicates that the assumption of sphericity is violated (p < .05).

Between Subjects Effects

	Sum of Squares	df	Mean Square	F	p
Residuals	106.279	18	5.904		

Note. Type III Sum of Squares

Descriptives

Descriptives

N	Device	TrialType	N	Mean	SD	SE	Coefficient of variation
1	Classical	non-Match	19	0.903	0.203	0.047	0.225
		Match	19	0.716	0.086	0.020	0.121
	VR	non-Match	19	1.283	0.262	0.060	0.204
		Match	19	1.245	0.274	0.063	0.220
2	Classical	non-Match	19	2.089	1.124	0.258	0.538
		Match	19	1.614	0.684	0.157	0.424
	VR	non-Match	19	1.930	0.715	0.164	0.370
		Match	19	1.698	0.561	0.129	0.331
3	Classical	non-Match	19	2.998	1.742	0.400	0.581
		Match	19	2.368	1.536	0.352	0.649
	VR	non-Match	19	2.738	1.484	0.340	0.542
		Match	19	2.250	0.918	0.211	0.408

Descriptives plots

Device: Classical

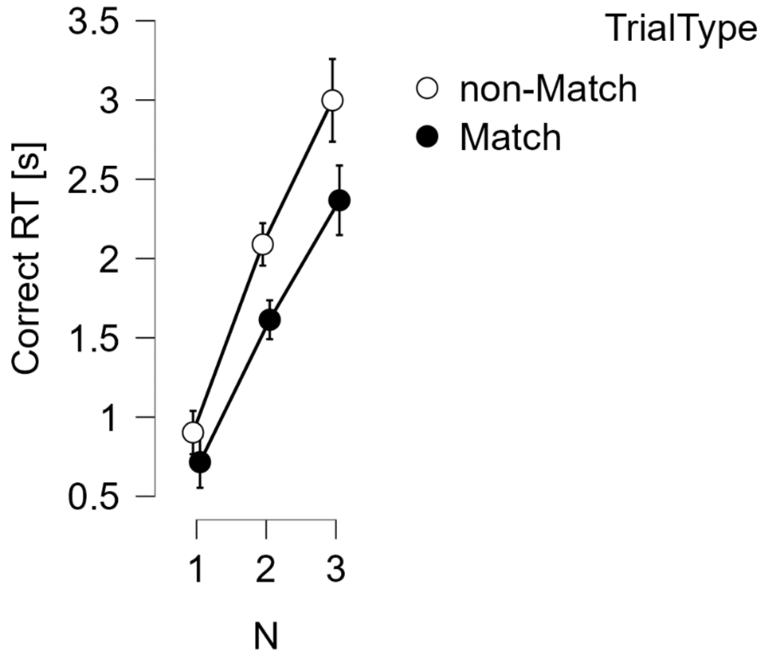

Device: VR

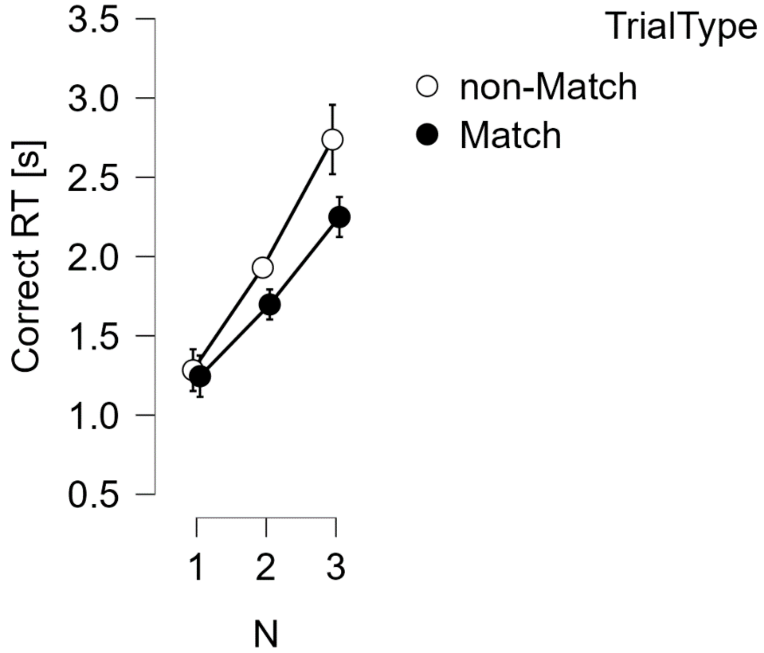

Assumption Checks

Test of Sphericity

	Mauchly's W	Approx. X^2	df	p-value	Greenhouse-Geisser ε	Huynh-Feldt ε	Lower Bound ε
N	0.239	24.310	2	< .001	0.568	0.581	0.500
N ✻ Device	0.644	7.468	2	0.024	0.738	0.788	0.500
N ✻ TrialType	0.706	5.915	2	0.052	0.773	0.832	0.500
N ✻ Device ✻ TrialType	0.796	3.888	2	0.143	0.830	0.904	0.500

Classical correct RT vs VR Halfway correct times

Within Subjects Effects

	Sphericity correction	Sum of Squares	df	Mean Square	F	p	η²	η²p
N	None	79.900 [a]	2.000 [a]	39.950 [a]	31.724 [a]	< .001 [a]	0.421	0.638
	Greenhouse-Geisser	79.900	1.145	69.757	31.724	< .001	0.421	0.638
Residuals	None	45.334	36.000	1.259				
	Greenhouse-Geisser	45.334	20.617	2.199				
Device	None	4.310	1.000	4.310	6.019	0.025	0.023	0.251
Residuals	None	12.890	18.000	0.716				
TrialType	None	5.475	1.000	5.475	15.588	< .001	0.029	0.464
Residuals	None	6.322	18.000	0.351				
N ✻ Device	None	7.047 [a]	2.000 [a]	3.523 [a]	8.936 [a]	< .001 [a]	0.037	0.332
	Greenhouse-Geisser	7.047	1.353	5.208	8.936	0.003	0.037	0.332
Residuals	None	14.194	36.000	0.394				
	Greenhouse-Geisser	14.194	24.355	0.583				
N ✻ TrialType	None	1.487	2.000	0.743	5.031	0.012	0.008	0.218
	Greenhouse-Geisser	1.487	1.645	0.904	5.031	0.018	0.008	0.218
Residuals	None	5.319	36.000	0.148				
	Greenhouse-Geisser	5.319	29.615	0.180				
Device ✻ TrialType	None	0.833	1.000	0.833	6.305	0.022	0.004	0.259
Residuals	None	2.379	18.000	0.132				
N ✻ Device ✻ TrialType	None	0.056	2.000	0.028	0.233	0.794	2.940×10^{-4}	0.013
	Greenhouse-Geisser	0.056	1.548	0.036	0.233	0.737	2.940×10^{-4}	0.013
Residuals	None	4.320	36.000	0.120				
	Greenhouse-Geisser	4.320	27.868	0.155				

Note. Sphericity corrections not available for factors with 2 levels.

Note. Type III Sum of Squares

[a] Mauchly's test of sphericity indicates that the assumption of sphericity is violated (p < .05).

Between Subjects Effects

	Sum of Squares	df	Mean Square	F	p
Residuals	86.460	18	4.803		

Note. Type III Sum of Squares

Descriptives

Descriptives

N	Device	TrialType	N	Mean	SD	SE	Coefficient of variation
1	Classical	non-Match	19	0.903	0.203	0.047	0.225
		Match	19	0.716	0.086	0.020	0.121
	VR	non-Match	19	1.015	0.176	0.040	0.173
		Match	19	0.987	0.244	0.056	0.248
2	Classical	non-Match	19	2.089	1.124	0.258	0.538
		Match	19	1.614	0.684	0.157	0.424
	VR	non-Match	19	1.574	0.609	0.140	0.387
		Match	19	1.409	0.457	0.105	0.324
3	Classical	non-Match	19	2.998	1.742	0.400	0.581
		Match	19	2.368	1.536	0.352	0.649
	VR	non-Match	19	2.213	1.140	0.261	0.515
		Match	19	1.838	0.837	0.192	0.455

Descriptives plots

Device: Classical

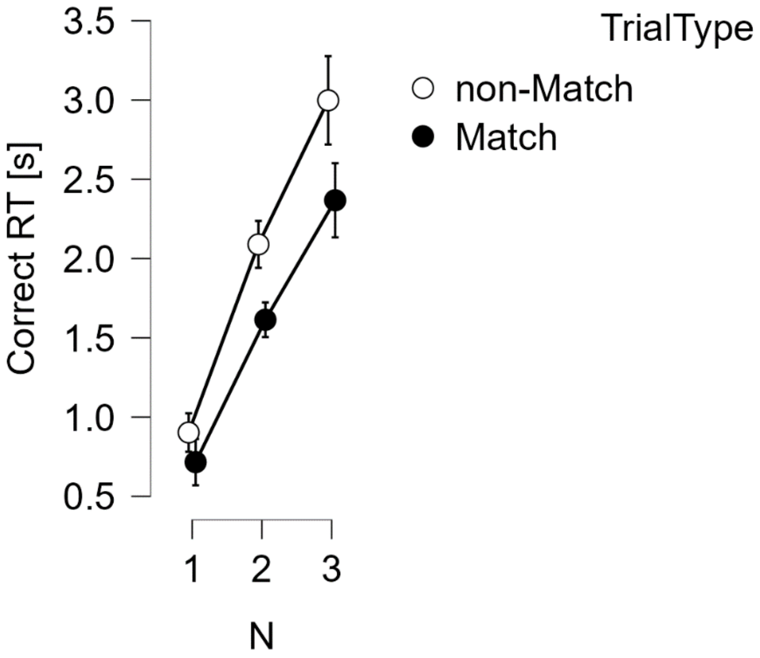

Device: VR

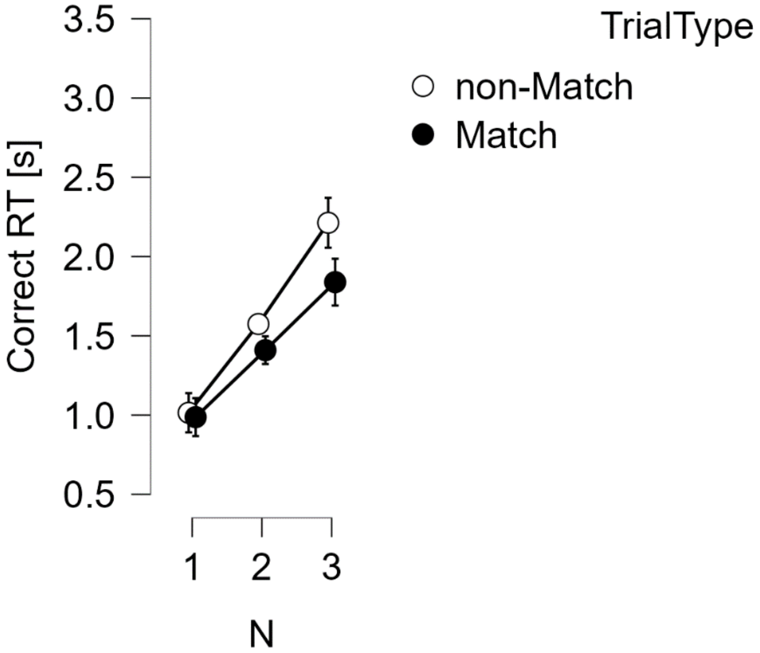

Assumption Checks

Test of Sphericity

	Mauchly's W	Approx. X^2	df	p-value	Greenhouse-Geisser ε	Huynh-Feldt ε	Lower Bound ε
N	0.254	23.305	2	< .001	0.573	0.586	0.500
N ✻ Device	0.522	11.056	2	0.004	0.677	0.712	0.500
N ✻ TrialType	0.784	4.128	2	0.127	0.823	0.895	0.500
N ✻ Device ✻ TrialType	0.708	5.866	2	0.053	0.774	0.833	0.500

Classical Accuracy vs VR Arrival Accuracy

Within Subjects Effects

	Sphericity correction	Sum of Squares	df	Mean Square	F	p	η^2	η^2_p
N	None	0.789 [a]	2.000 [a]	0.395 [a]	37.592 [a]	< .001 [a]	0.330	0.676
	Greenhouse-Geisser	0.789	1.173	0.672	37.592	< .001	0.330	0.676
Residuals	None	0.378	36.000	0.010				
	Greenhouse-Geisser	0.378	21.121	0.018				
Device	None	0.013	1.000	0.013	2.050	0.169	0.005	0.102
Residuals	None	0.114	18.000	0.006				
TrialType	None	0.249	1.000	0.249	22.169	< .001	0.104	0.552
Residuals	None	0.202	18.000	0.011				
N ✻ Device	None	0.003 [a]	2.000 [a]	0.002 [a]	0.401 [a]	0.673 [a]	0.001	0.022
	Greenhouse-Geisser	0.003	1.393	0.002	0.401	0.600	0.001	0.022
Residuals	None	0.135	36.000	0.004				
	Greenhouse-Geisser	0.135	25.079	0.005				
N ✻ TrialType	None	0.141 [a]	2.000 [a]	0.071 [a]	12.199 [a]	< .001 [a]	0.059	0.404
	Greenhouse-Geisser	0.141	1.121	0.126	12.199	0.002	0.059	0.404
Residuals	None	0.209	36.000	0.006				
	Greenhouse-Geisser	0.209	20.181	0.010				
Device ✻ TrialType	None	0.008	1.000	0.008	2.495	0.132	0.003	0.122
Residuals	None	0.059	18.000	0.003				
N ✻ Device ✻ TrialType	None	0.003	2.000	0.001	0.509	0.605	0.001	0.028
	Greenhouse-Geisser	0.003	1.619	0.002	0.509	0.568	0.001	0.028
Residuals	None	0.090	36.000	0.003				
	Greenhouse-Geisser	0.090	29.149	0.003				

Note. Sphericity corrections not available for factors with 2 levels.

Note. Type III Sum of Squares

[a] Mauchly's test of sphericity indicates that the assumption of sphericity is violated ($p < .05$).

Between Subjects Effects

	Sum of Squares	df	Mean Square	F	p
Residuals	0.444	18	0.025		

Note. Type III Sum of Squares

Descriptives

Descriptives

N	Device	TrialType	N	Mean	SD	SE	Coefficient of variation
1	Classical	non-Match	19	0.991	0.013	0.003	0.013
		Match	19	0.951	0.045	0.010	0.047
	VR	non-Match	19	0.984	0.023	0.005	0.023
		Match	19	0.986	0.026	0.006	0.026
2	Classical	non-Match	19	0.969	0.029	0.007	0.029
		Match	19	0.915	0.077	0.018	0.084
	VR	non-Match	19	0.967	0.045	0.010	0.047
		Match	19	0.931	0.072	0.017	0.078
3	Classical	non-Match	19	0.898	0.067	0.015	0.075
		Match	19	0.758	0.162	0.037	0.214
	VR	non-Match	19	0.917	0.083	0.019	0.090
		Match	19	0.788	0.188	0.043	0.239

Descriptives plots

Device: Classical

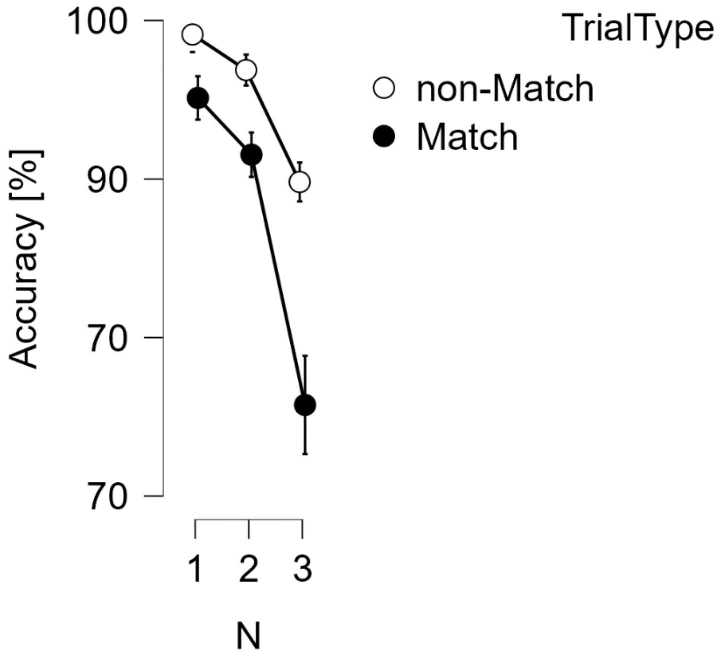

Device: VR

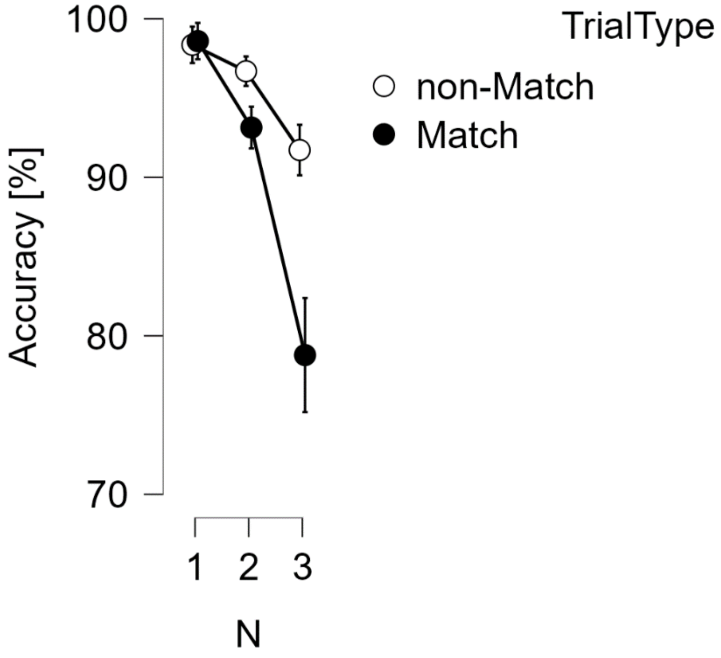

Assumption Checks

Test of Sphericity

	Mauchly's W	Approx. X^2	df	p-value	Greenhouse-Geisser ε	Huynh-Feldt ε	Lower Bound ε
N	0.296	20.720	2	< .001	0.587	0.603	0.500
N ✻ Device	0.565	9.719	2	0.008	0.697	0.737	0.500
N ✻ TrialType	0.216	26.038	2	< .001	0.561	0.572	0.500
N ✻ Device ✻ TrialType	0.765	4.555	2	0.103	0.810	0.878	0.500

Classical Accuracy vs VR Halfway Accuracy

Within Subjects Effects

	Sphericity correction	Sum of Squares	df	Mean Square	F	p	η^2	η^2_p
N	None	0.867 [a]	2.000 [a]	0.434 [a]	43.628 [a]	< .001 [a]	0.347	0.708
	Greenhouse-Geisser	0.867	1.202	0.721	43.628	< .001	0.347	0.708
Residuals	None	0.358	36.000	0.010				
	Greenhouse-Geisser	0.358	21.641	0.017				
Device	None	5.014×10^{-4}	1.000	5.014×10^{-4}	0.079	0.781	2.006×10^{-4}	0.004
Residuals	None	0.114	18.000	0.006				
TrialType	None	0.192	1.000	0.192	15.473	< .001	0.077	0.462
Residuals	None	0.223	18.000	0.012				
N ✻ Device	None	0.002 [a]	2.000 [a]	7.996×10^{-4} [a]	0.185 [a]	0.832 [a]	6.398×10^{-4}	0.010
	Greenhouse-Geisser	0.002	1.340	0.001	0.185	0.743	6.398×10^{-4}	0.010
Residuals	None	0.156	36.000	0.004				
	Greenhouse-Geisser	0.156	24.121	0.006				
N ✻ TrialType	None	0.092 [a]	2.000 [a]	0.046 [a]	6.585 [a]	0.004 [a]	0.037	0.268
	Greenhouse-Geisser	0.092	1.125	0.082	6.585	0.016	0.037	0.268
Residuals	None	0.252	36.000	0.007				
	Greenhouse-Geisser	0.252	20.241	0.012				
Device ✻ TrialType	None	0.023	1.000	0.023	4.380	0.051	0.009	0.196
Residuals	None	0.094	18.000	0.005				
N ✻ Device ✻ TrialType	None	0.003 [a]	2.000 [a]	0.002 [a]	0.485 [a]	0.620 [a]	0.001	0.026
	Greenhouse-Geisser	0.003	1.442	0.002	0.485	0.560	0.001	0.026
Residuals	None	0.123	36.000	0.003				
	Greenhouse-Geisser	0.123	25.954	0.005				

Note. Sphericity corrections not available for factors with 2 levels.

Within Subjects Effects

	Sphericity correction	Sum of Squares	df	Mean Square	F	p	η²	η²ₚ

Note. Type III Sum of Squares

[a] Mauchly's test of sphericity indicates that the assumption of sphericity is violated (p < .05).

Between Subjects Effects

	Sum of Squares	df	Mean Square	F	p
Residuals	0.414	18	0.023		

Note. Type III Sum of Squares

Descriptives

Descriptives

N	Device	TrialType	N	Mean	SD	SE	Coefficient of variation
1	Classical	non-Match	19	0.991	0.013	0.003	0.013
		Match	19	0.951	0.045	0.010	0.047
	VR	non-Match	19	0.977	0.025	0.006	0.026
		Match	19	0.982	0.026	0.006	0.026
2	Classical	non-Match	19	0.969	0.029	0.007	0.029
		Match	19	0.915	0.077	0.018	0.084
	VR	non-Match	19	0.955	0.050	0.012	0.053
		Match	19	0.921	0.072	0.016	0.078
3	Classical	non-Match	19	0.898	0.067	0.015	0.075
		Match	19	0.758	0.162	0.037	0.214
	VR	non-Match	19	0.875	0.102	0.023	0.116
		Match	19	0.790	0.193	0.044	0.244

Descriptives plots

Device: Classical

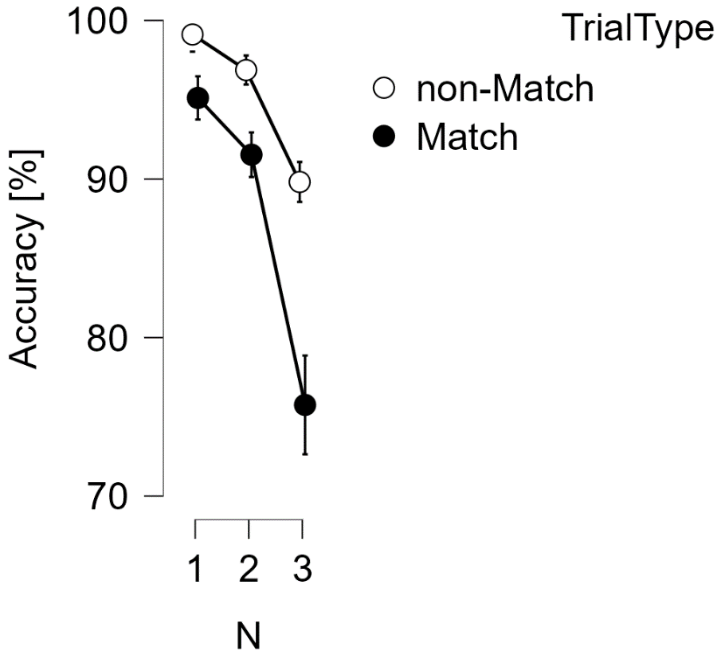

Device: VR

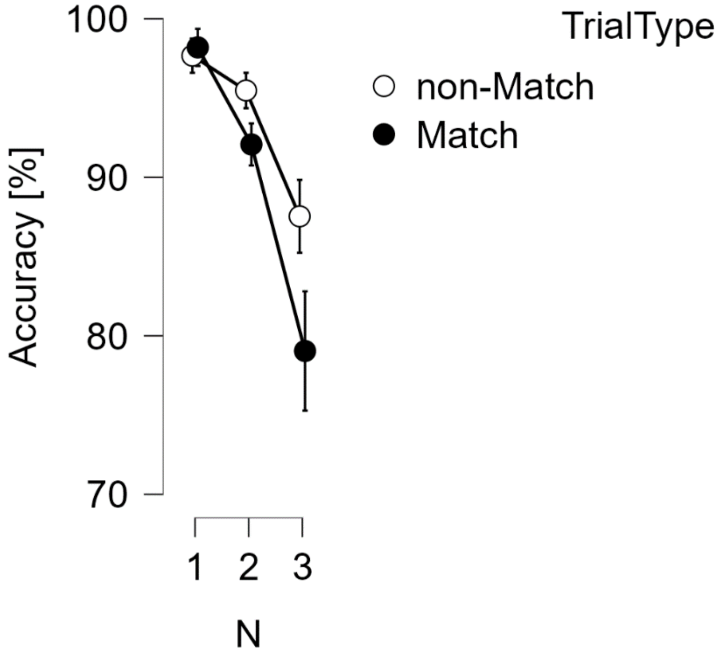

Assumption Checks

Test of Sphericity

	Mauchly's W	Approx. X^2	df	p-value	Greenhouse-Geisser ε	Huynh-Feldt ε	Lower Bound ε
N	0.337	18.515	2	< .001	0.601	0.620	0.500
N ✻ Device	0.508	11.529	2	0.003	0.670	0.704	0.500
N ✻ TrialType	0.221	25.627	2	< .001	0.562	0.574	0.500
N ✻ Device ✻ TrialType	0.613	8.321	2	0.016	0.721	0.767	0.500

Correct Classical RT

Within Subjects Effects

	Sphericity correction	Sum of Squares	df	Mean Square	F	p	η²	η²ₚ
N	None	66.977 [a]	2.000 [a]	33.488 [a]	26.346 [a]	< .001 [a]	0.514	0.594
	Greenhouse-Geisser	66.977	1.188	56.383	26.346	< .001	0.514	0.594
Residuals	None	45.759	36.000	1.271				
	Greenhouse-Geisser	45.759	21.382	2.140				
TrialType	None	5.290	1.000	5.290	16.618	< .001	0.041	0.480
Residuals	None	5.730	18.000	0.318				
N ✻ TrialType	None	0.963	2.000	0.481	3.156	0.055	0.007	0.149
	Greenhouse-Geisser	0.963	1.817	0.530	3.156	0.060	0.007	0.149
Residuals	None	5.491	36.000	0.153				
	Greenhouse-Geisser	5.491	32.702	0.168				

Note. Sphericity corrections not available for factors with 2 levels.

Note. Type III Sum of Squares

[a] Mauchly's test of sphericity indicates that the assumption of sphericity is violated (p < .05).

Between Subjects Effects

	Sum of Squares	df	Mean Square	F	p
Residuals	72.173	18	4.010		

Note. Type III Sum of Squares

Descriptives

Descriptives

N	TrialType	N	Mean	SD	SE	Coefficient of variation
1	non-Match	19	0.903	0.203	0.047	0.225
	Match	19	0.716	0.086	0.020	0.121
2	non-Match	19	2.089	1.124	0.258	0.538
	Match	19	1.614	0.684	0.157	0.424
3	non-Match	19	2.998	1.742	0.400	0.581

Descriptives

N	TrialType	N	Mean	SD	SE	Coefficient of variation
	Match	19	2.368	1.536	0.352	0.649

Descriptives plots

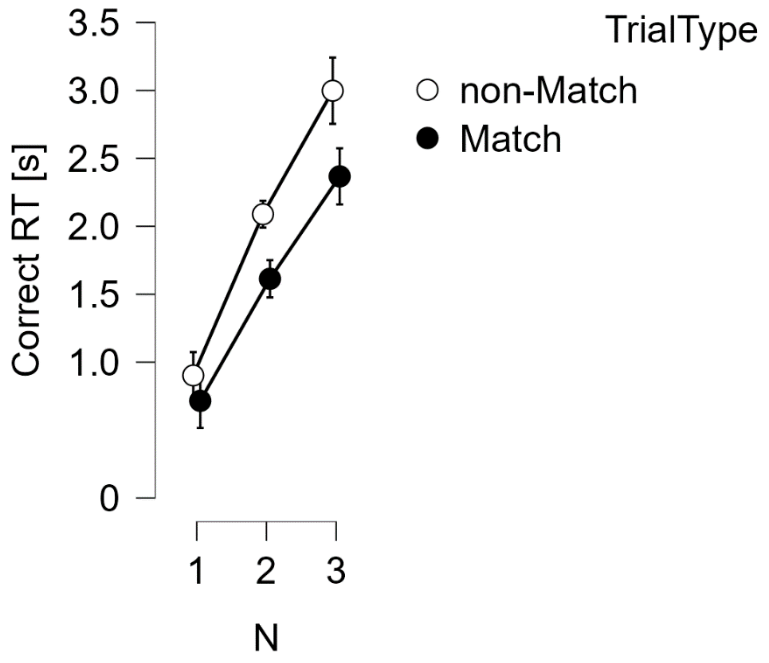

Assumption Checks

Test of Sphericity

	Mauchly's W	Approx. X²	df	p-value	Greenhouse-Geisser ε	Huynh-Feldt ε	Lower Bound ε
N	0.316	19.565	2	< .001	0.594	0.612	0.500
N ✻ TrialType	0.899	1.807	2	0.405	0.908	1.000	0.500

Correct VR Arrival times

Within Subjects Effects

	Sphericity correction	Sum of Squares	df	Mean Square	F	p	η²	η²ₚ
N	None	28.838 [a]	2.000 [a]	14.419 [a]	24.380 [a]	< .001 [a]	0.463	0.575
	Greenhouse-Geisser	28.838	1.212	23.802	24.380	< .001	0.463	0.575
Residuals	None	21.292	36.000	0.591				
	Greenhouse-Geisser	21.292	21.809	0.976				
TrialType	None	1.823	1.000	1.823	7.977	0.011	0.029	0.307
Residuals	None	4.113	18.000	0.229				
N ✻ TrialType	None	0.970 [a]	2.000 [a]	0.485 [a]	3.352 [a]	0.046 [a]	0.016	0.157
	Greenhouse-Geisser	0.970	1.137	0.854	3.352	0.077	0.016	0.157
Residuals	None	5.210	36.000	0.145				
	Greenhouse-Geisser	5.210	20.458	0.255				

Note. Sphericity corrections not available for factors with 2 levels.

Note. Type III Sum of Squares

[a] Mauchly's test of sphericity indicates that the assumption of sphericity is violated (p < .05).

Between Subjects Effects

	Sum of Squares	df	Mean Square	F	p
Residuals	41.626	18	2.313		

Note. Type III Sum of Squares

Descriptives

Descriptives

N	TrialType	N	Mean	SD	SE	Coefficient of variation
1	non-Match	19	1.283	0.262	0.060	0.204
	Match	19	1.245	0.274	0.063	0.220
2	non-Match	19	1.930	0.715	0.164	0.370
	Match	19	1.698	0.561	0.129	0.331
3	non-Match	19	2.738	1.484	0.340	0.542

Descriptives

N	TrialType	N	Mean	SD	SE	Coefficient of variation
	Match	19	2.250	0.918	0.211	0.408

Descriptives plots

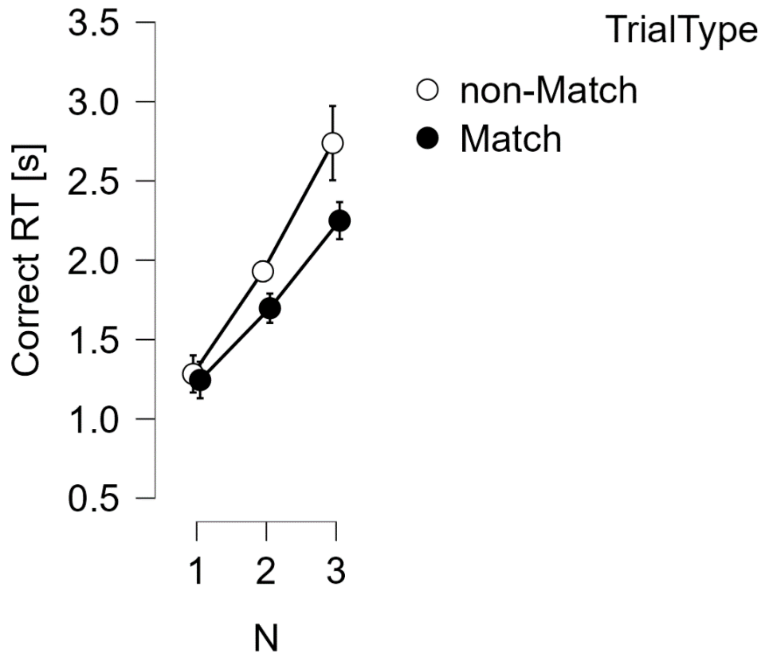

Assumption Checks

Test of Sphericity

	Mauchly's W	Approx. X²	df	p-value	Greenhouse-Geisser ε	Huynh-Feldt ε	Lower Bound ε
N	0.349	17.881	2	< .001	0.606	0.626	0.500
N ✻ TrialType	0.240	24.239	2	< .001	0.568	0.581	0.500

Correct VR Halfway times

Within Subjects Effects

	Sphericity correction	Sum of Squares	df	Mean Square	F	p	η²	η²ₚ
N	None	19.970 [a]	2.000 [a]	9.985 [a]	26.107 [a]	< .001 [a]	0.470	0.592
	Greenhouse-Geisser	19.970	1.223	16.334	26.107	< .001	0.470	0.592
Residuals	None	13.769	36.000	0.382				
	Greenhouse-Geisser	13.769	22.006	0.626				
TrialType	None	1.018	1.000	1.018	6.168	0.023	0.024	0.255
Residuals	None	2.971	18.000	0.165				
N ✻ TrialType	None	0.580 [a]	2.000 [a]	0.290 [a]	2.516 [a]	0.095 [a]	0.014	0.123
	Greenhouse-Geisser	0.580	1.155	0.502	2.516	0.124	0.014	0.123
Residuals	None	4.149	36.000	0.115				
	Greenhouse-Geisser	4.149	20.792	0.200				

Note. Sphericity corrections not available for factors with 2 levels.

Note. Type III Sum of Squares

[a] Mauchly's test of sphericity indicates that the assumption of sphericity is violated (p < .05).

Between Subjects Effects

	Sum of Squares	df	Mean Square	F	p
Residuals	27.178	18	1.510		

Note. Type III Sum of Squares

Descriptives

Descriptives

N	TrialType	N	Mean	SD	SE	Coefficient of variation
1	non-Match	19	1.015	0.176	0.040	0.173
	Match	19	0.987	0.244	0.056	0.248
2	non-Match	19	1.574	0.609	0.140	0.387
	Match	19	1.409	0.457	0.105	0.324
3	non-Match	19	2.213	1.140	0.261	0.515

Descriptives

N	TrialType	N	Mean	SD	SE	Coefficient of variation
	Match	19	1.838	0.837	0.192	0.455

Descriptives plots

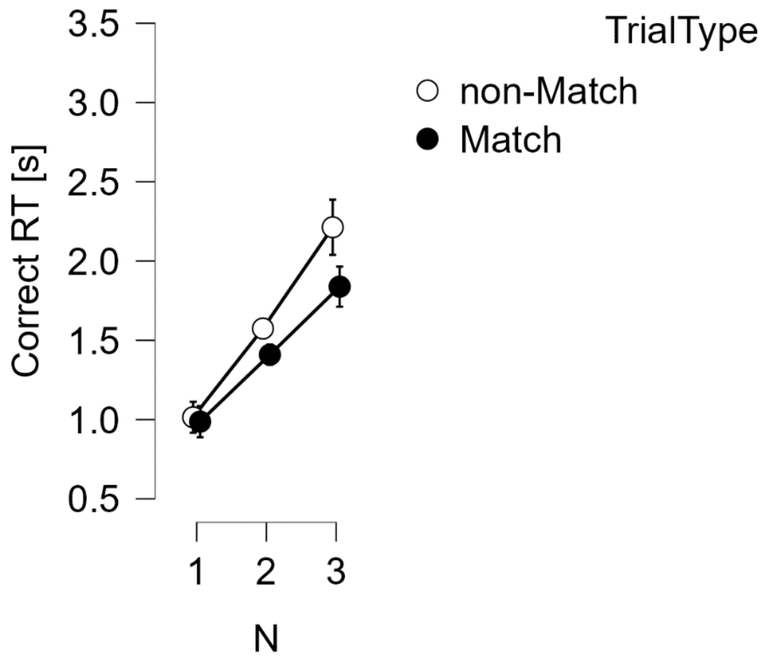

Assumption Checks

Test of Sphericity

	Mauchly's W	Approx. X²	df	p-value	Greenhouse-Geisser ε	Huynh-Feldt ε	Lower Bound ε
N	0.364	17.175	2	< .001	0.611	0.633	0.500
N ✻ TrialType	0.269	22.349	2	< .001	0.578	0.592	0.500

Accuracy Classical

Within Subjects Effects

	Sphericity correction	Sum of Squares	df	Mean Square	F	p	η²	η²ₚ
N	None	0.436 [a]	2.000 [a]	0.218 [a]	35.211 [a]	< .001 [a]	0.380	0.662
	Greenhouse-Geisser	0.436	1.348	0.324	35.211	< .001	0.380	0.662
Residuals	None	0.223	36.000	0.006				
	Greenhouse-Geisser	0.223	24.259	0.009				
TrialType	None	0.174	1.000	0.174	20.578	< .001	0.151	0.533
Residuals	None	0.152	18.000	0.008				
N ∗ TrialType	None	0.057 [a]	2.000 [a]	0.028 [a]	9.677 [a]	< .001 [a]	0.049	0.350
	Greenhouse-Geisser	0.057	1.408	0.040	9.677	0.002	0.049	0.350
Residuals	None	0.106	36.000	0.003				
	Greenhouse-Geisser	0.106	25.339	0.004				

Note. Sphericity corrections not available for factors with 2 levels.

Note. Type III Sum of Squares

[a] Mauchly's test of sphericity indicates that the assumption of sphericity is violated (p < .05).

Between Subjects Effects

	Sum of Squares	df	Mean Square	F	p
Residuals	0.235	18	0.013		

Note. Type III Sum of Squares

Descriptives

Descriptives

N	TrialType	N	Mean	SD	SE	Coefficient of variation
1	non-Match	19	0.991	0.013	0.003	0.013
	Match	19	0.951	0.045	0.010	0.047
2	non-Match	19	0.969	0.029	0.007	0.029
	Match	19	0.915	0.077	0.018	0.084
3	non-Match	19	0.898	0.067	0.015	0.075

Descriptives

N	TrialType	N	Mean	SD	SE	Coefficient of variation
	Match	19	0.758	0.162	0.037	0.214

Descriptives plots

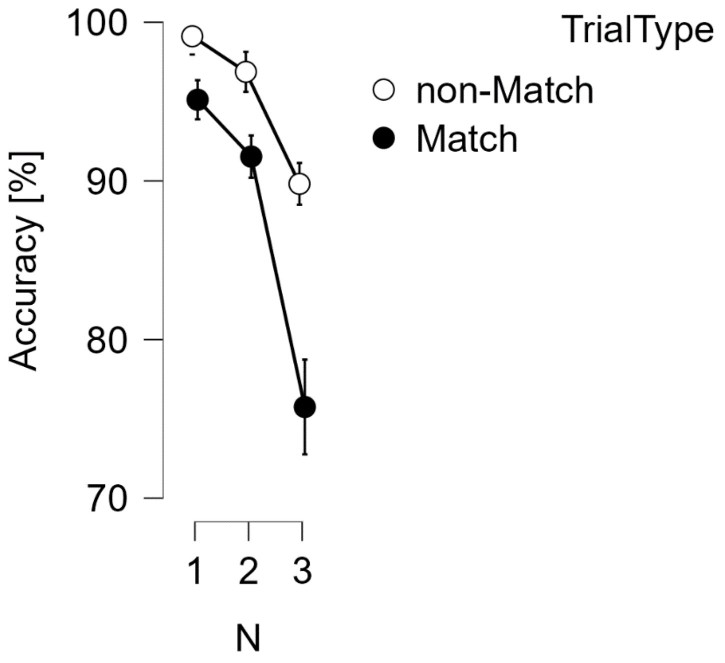

Assumption Checks

Test of Sphericity

	Mauchly's W	Approx. X²	df	p-value	Greenhouse-Geisser ε	Huynh-Feldt ε	Lower Bound ε
N	0.516	11.248	2	0.004	0.674	0.709	0.500
N ✻ TrialType	0.579	9.281	2	0.010	0.704	0.746	0.500

Accuracy VR Arrival

Within Subjects Effects

	Sphericity correction	Sum of Squares	df	Mean Square	F	p	η²	η²ₚ
N	None	0.356 [a]	2.000 [a]	0.178 [a]	22.088 [a]	< .001 [a]	0.318	0.551
	Greenhouse-Geisser	0.356	1.275	0.279	22.088	< .001	0.318	0.551
Residuals	None	0.290	36.000	0.008				
	Greenhouse-Geisser	0.290	22.943	0.013				
TrialType	None	0.084	1.000	0.084	13.815	0.002	0.075	0.434
Residuals	None	0.109	18.000	0.006				
N ✻ TrialType	None	0.087 [a]	2.000 [a]	0.044 [a]	8.131 [a]	0.001 [a]	0.078	0.311
	Greenhouse-Geisser	0.087	1.188	0.074	8.131	0.007	0.078	0.311
Residuals	None	0.193	36.000	0.005				
	Greenhouse-Geisser	0.193	21.381	0.009				

Note. Sphericity corrections not available for factors with 2 levels.

Note. Type III Sum of Squares

[a] Mauchly's test of sphericity indicates that the assumption of sphericity is violated (p < .05).

Between Subjects Effects

	Sum of Squares	df	Mean Square	F	p
Residuals	0.323	18	0.018		

Note. Type III Sum of Squares

Descriptives

Descriptives

N	TrialType	N	Mean	SD	SE	Coefficient of variation
1	non-Match	19	0.984	0.023	0.005	0.023
	Match	19	0.986	0.026	0.006	0.026
2	non-Match	19	0.967	0.045	0.010	0.047
	Match	19	0.931	0.072	0.017	0.078
3	non-Match	19	0.917	0.083	0.019	0.090

Descriptives

N	TrialType	N	Mean	SD	SE	Coefficient of variation
	Match	19	0.788	0.188	0.043	0.239

Descriptives plots

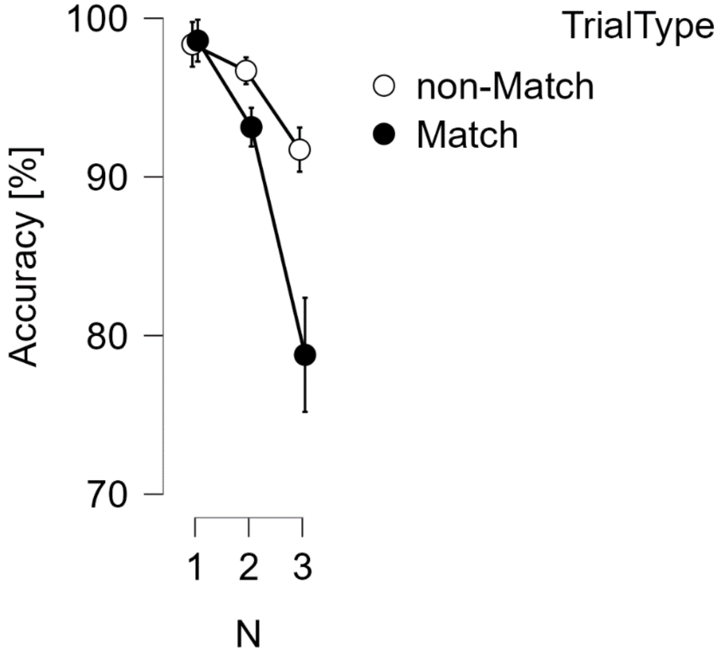

Assumption Checks

Test of Sphericity

	Mauchly's W	Approx. X²	df	p-value	Greenhouse-Geisser ε	Huynh-Feldt ε	Lower Bound ε
N	0.431	14.313	2	< .001	0.637	0.664	0.500
N ✻ TrialType	0.316	19.571	2	< .001	0.594	0.612	0.500

Accuracy VR Halfway

Within Subjects Effects

	Sphericity correction	Sum of Squares	df	Mean Square	F	p	η^2	η^2_p
N	None	0.433 [a]	2.000 [a]	0.216 [a]	26.786 [a]	< .001 [a]	0.349	0.598
	Greenhouse-Geisser	0.433	1.341	0.323	26.786	< .001	0.349	0.598
Residuals	None	0.291	36.000	0.008				
	Greenhouse-Geisser	0.291	24.132	0.012				
TrialType	None	0.041	1.000	0.041	4.462	0.049	0.033	0.199
Residuals	None	0.165	18.000	0.009				
N ✻ TrialType	None	0.039 [a]	2.000 [a]	0.019 [a]	2.590 [a]	0.089 [a]	0.031	0.126
	Greenhouse-Geisser	0.039	1.168	0.033	2.590	0.119	0.031	0.126
Residuals	None	0.270	36.000	0.007				
	Greenhouse-Geisser	0.270	21.018	0.013				

Note. Sphericity corrections not available for factors with 2 levels.

Note. Type III Sum of Squares

[a] Mauchly's test of sphericity indicates that the assumption of sphericity is violated (p < .05).

Between Subjects Effects

	Sum of Squares	df	Mean Square	F	p
Residuals	0.293	18	0.016		

Note. Type III Sum of Squares

Descriptives

Descriptives

N	TrialType	N	Mean	SD	SE	Coefficient of variation
1	non-Match	19	0.977	0.025	0.006	0.026
	Match	19	0.982	0.026	0.006	0.026
2	non-Match	19	0.955	0.050	0.012	0.053
	Match	19	0.921	0.072	0.016	0.078
3	non-Match	19	0.875	0.102	0.023	0.116

Descriptives

N	TrialType	N	Mean	SD	SE	Coefficient of variation
	Match	19	0.790	0.193	0.044	0.244

Descriptives plots

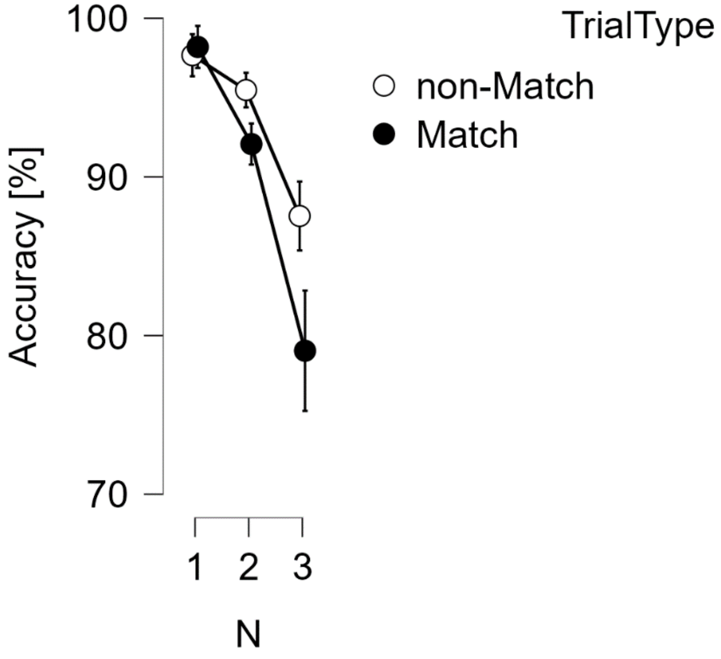

Assumption Checks

Test of Sphericity

	Mauchly's W	Approx. Χ²	df	p-value	Greenhouse-Geisser ε	Huynh-Feldt ε	Lower Bound ε
N	0.508	11.508	2	0.003	0.670	0.704	0.500
N ✻ TrialType	0.287	21.209	2	< .001	0.584	0.600	0.500

Part 2: ANOVA results with trimming

Trimming Procedure

The data was trimmed as follows:

We started with 8664 total number of trials before any trimming, excluding task irrelevant trials (Task irrelevant trails refers to trials with no correct response, which are the first trial in N1, the first two trials in N2, and the first 3 trials for N3).

The following process was done for each condition (Device/Trial Type/ N) separately.

1) Outlier detection and trimming using interquartile ranges (IQR), outliers were defined as trials that are outside the range of (Q1 -1.5*IQR - Q3+1.5*IQR)
2) Standard deviation (SD) trimming, outliers were defined as trials outside the range of mean + 2* SD.

Table 1. Trimming procedure

Trimming Type	Condition	Total # of Trials	# of Task relevant Trials*	# Remaining	# Removed	% Remaining	% Removed
3-way-ANOVAs							
Outlier Rejection	Classical vs Arrival	9120	8664	8125	539	93.77	6.22
Outlier Rejection	Classical vs Halfway	9120	8664	8119	545	93.7	6.29
Standard Deviation Trimming	Classical vs Arrival	9120	8125	7725	400	95.07	4.92
Standard Deviation Trimming	Classical vs Halfway	9120	8119	7721	398	95.09	4.9
2-way-ANOVAs							
Outlier Rejection	Classical	4560	4332	4091	241	94.43	5.56
Outlier Rejection	VR Arrival	4560	4332	4034	298	93.12	6.87
Outlier Rejection	VR Halfway	4560	4332	4028	304	92.98	7.01
Standard Deviation Trimming	Classical	4560	4091	3884	207	94.94	5.05
Standard Deviation Trimming	VR Arrival	4560	4034	3841	193	95.21	4.78
Standard Deviation Trimming	VR Halfway	4560	4028	3837	191	95.25	4.74

Error trimming	Classical	4560	3884	3643	241	93.79	6.2
Error trimming	VR Arrival	4560	3841	3543	298	92.24	7.75
Error trimming	VR Halfway	4560	3837	3533	304	92.07	7.92
*Task relevant means: excluding first trial in N1, First 2 trials in N2 first 3 trials in N3, as well excluding data trimmed from outlier rejection in the Standard Deviation Trimming							

Classical correct RT vs VR Arrival correct times

Within Subjects Effects

	Sphericity correction	Sum of Squares	df	Mean Square	F	p	η²	η²p
N	None	64.754 [a]	2.000 [a]	32.377 [a]	26.446 [a]	< .001 [a]	0.434	0.595
	Greenhouse-Geisser	64.754	1.135	57.039	26.446	< .001	0.434	0.595
Residuals	None	44.073	36.000	1.224				
	Greenhouse-Geisser	44.073	20.435	2.157				
Device	None	0.351	1.000	0.351	0.996	0.332	0.002	0.052
Residuals	None	6.342	18.000	0.352				
Trial Type	None	4.443	1.000	4.443	12.692	0.002	0.030	0.414
Residuals	None	6.301	18.000	0.350				
N ✻ Device	None	4.087 [a]	2.000 [a]	2.044 [a]	8.267 [a]	0.001 [a]	0.027	0.315
	Greenhouse-Geisser	4.087	1.323	3.090	8.267	0.005	0.027	0.315
Residuals	None	8.900	36.000	0.247				
	Greenhouse-Geisser	8.900	23.809	0.374				
N ✻ Trial Type	None	1.286 [a]	2.000 [a]	0.643 [a]	6.066 [a]	0.005 [a]	0.009	0.252
	Greenhouse-Geisser	1.286	1.432	0.898	6.066	0.013	0.009	0.252
Residuals	None	3.815	36.000	0.106				
	Greenhouse-Geisser	3.815	25.776	0.148				
Device ✻ Trial Type	None	0.193	1.000	0.193	2.478	0.133	0.001	0.121
Residuals	None	1.404	18.000	0.078				
N ✻ Device ✻ Trial Type	None	0.207	2.000	0.103	1.196	0.314	0.001	0.062
	Greenhouse-Geisser	0.207	1.543	0.134	1.196	0.307	0.001	0.062
Residuals	None	3.113	36.000	0.086				
	Greenhouse-Geisser	3.113	27.766	0.112				

Note. Sphericity corrections not available for factors with 2 levels.

Note. Type III Sum of Squares

[a] Mauchly's test of sphericity indicates that the assumption of sphericity is violated ($p < .05$).

Between Subjects Effects

	Sum of Squares	df	Mean Square	F	p
Residuals	78.166	18	4.343		

Note. Type III Sum of Squares

Descriptives

Descriptives

N	Device	Trial Type	N	Mean	SD	SE	Coefficient of variation
1	Classical	non-Match	19	0.779	0.139	0.032	0.179
		Match	19	0.682	0.075	0.017	0.110
	VR	non-Match	19	1.191	0.242	0.056	0.204
		Match	19	1.123	0.201	0.046	0.179
2	Classical	non-Match	19	1.811	0.951	0.218	0.525
		Match	19	1.358	0.586	0.134	0.432
	VR	non-Match	19	1.702	0.644	0.148	0.379
		Match	19	1.536	0.466	0.107	0.303
3	Classical	non-Match	19	2.593	1.518	0.348	0.585
		Match	19	2.130	1.389	0.319	0.652
	VR	non-Match	19	2.351	1.229	0.282	0.523
		Match	19	1.922	0.837	0.192	0.436

Descriptives plots

Device: Classical

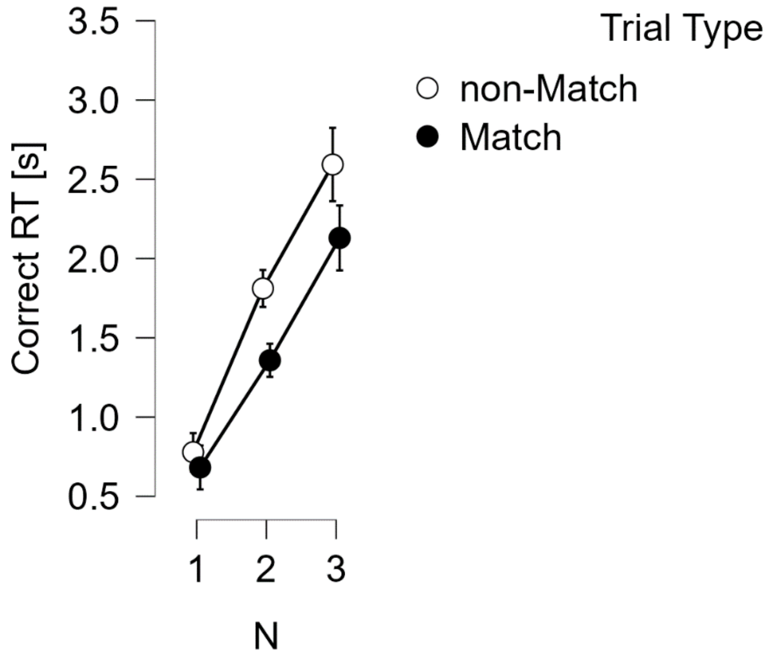

Device: VR

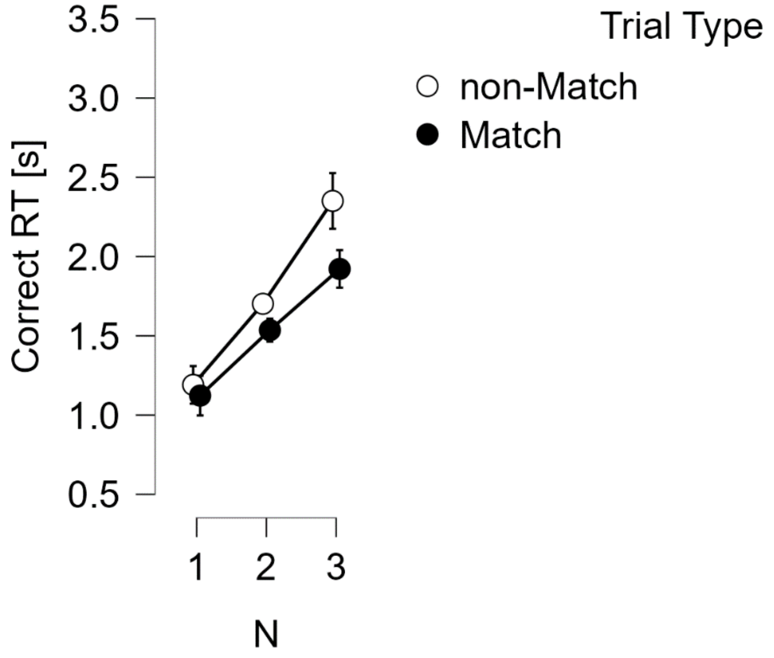

Assumption Checks

Test of Sphericity

	Mauchly's W	Approx. X^2	df	p-value	Greenhouse-Geisser ε	Huynh-Feldt ε	Lower Bound ε
N	0.238	24.382	2	< .001	0.568	0.580	0.500
N ✻ Device	0.488	12.197	2	0.002	0.661	0.694	0.500
N ✻ Trial Type	0.603	8.590	2	0.014	0.716	0.761	0.500
N ✻ Device ✻ Trial Type	0.703	5.980	2	0.050	0.771	0.830	0.500

Classical correct RT vs VR Halfway correct times

Within Subjects Effects

	Sphericity correction	Sum of Squares	df	Mean Square	F	p	η^2	η^2_p
N	None	55.426 [a]	2.000 [a]	27.713 [a]	28.524 [a]	< .001 [a]	0.386	0.613
	Greenhouse-Geisser	55.426	1.146	48.357	28.524	< .001	0.386	0.613
Residuals	None	34.976	36.000	0.972				
	Greenhouse-Geisser	34.976	20.631	1.695				
Device	None	3.776	1.000	3.776	5.505	0.031	0.026	0.234
Residuals	None	12.346	18.000	0.686				
Trial Type	None	3.613	1.000	3.613	11.163	0.004	0.025	0.383
Residuals	None	5.825	18.000	0.324				
N ✻ Device	None	6.842 [a]	2.000 [a]	3.421 [a]	11.009 [a]	< .001 [a]	0.048	0.380
	Greenhouse-Geisser	6.842	1.215	5.631	11.009	0.002	0.048	0.380
Residuals	None	11.187	36.000	0.311				
	Greenhouse-Geisser	11.187	21.871	0.512				
N ✻ Trial Type	None	0.880	2.000	0.440	4.402	0.019	0.006	0.197
	Greenhouse-Geisser	0.880	1.701	0.517	4.402	0.026	0.006	0.197
Residuals	None	3.597	36.000	0.100				
	Greenhouse-Geisser	3.597	30.614	0.118				
Device ✻ Trial Type	None	0.419	1.000	0.419	4.451	0.049	0.003	0.198
Residuals	None	1.692	18.000	0.094				
N ✻ Device ✻ Trial Type	None	0.151	2.000	0.076	0.950	0.396	0.001	0.050
	Greenhouse-Geisser	0.151	1.551	0.098	0.950	0.378	0.001	0.050
Residuals	None	2.867	36.000	0.080				
	Greenhouse-Geisser	2.867	27.918	0.103				

Note. Sphericity corrections not available for factors with 2 levels.

Note. Type III Sum of Squares

[a] Mauchly's test of sphericity indicates that the assumption of sphericity is violated ($p < .05$).

Between Subjects Effects

	Sum of Squares	df	Mean Square	F	p
Residuals	60.936	18	3.385		

Note. Type III Sum of Squares

Descriptives

Descriptives

N	Device	Trial Type	N	Mean	SD	SE	Coefficient of variation
1	Classical	non-Match	19	0.779	0.139	0.032	0.179
		Match	19	0.682	0.075	0.017	0.110
	VR	non-Match	19	0.943	0.131	0.030	0.139
		Match	19	0.881	0.130	0.030	0.147
2	Classical	non-Match	19	1.811	0.951	0.218	0.525
		Match	19	1.358	0.586	0.134	0.432
	VR	non-Match	19	1.382	0.554	0.127	0.401
		Match	19	1.212	0.362	0.083	0.298
3	Classical	non-Match	19	2.593	1.518	0.348	0.585
		Match	19	2.130	1.389	0.319	0.652
	VR	non-Match	19	1.829	0.971	0.223	0.531
		Match	19	1.562	0.701	0.161	0.448

Descriptives plots

Device: Classical

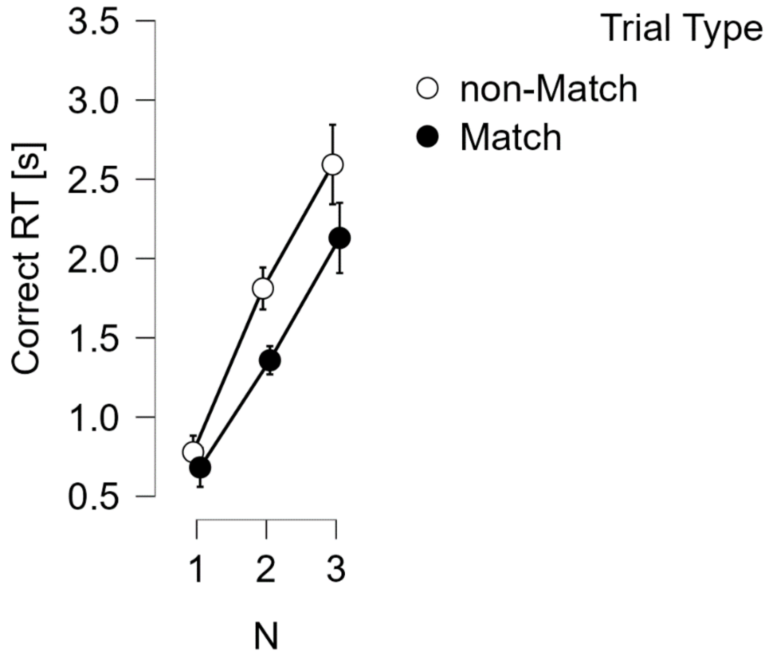

Device: VR

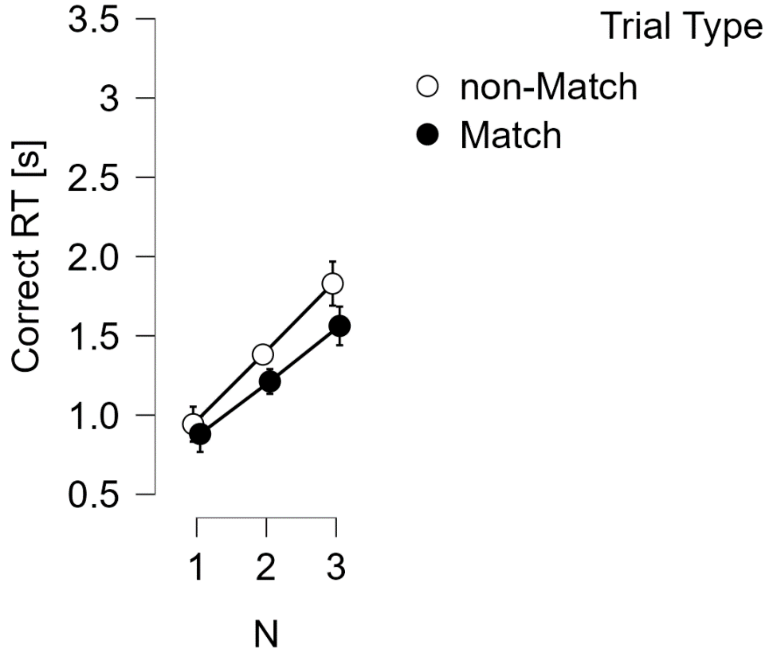

Assumption Checks

Test of Sphericity

	Mauchly's W	Approx. X^2	df	p-value	Greenhouse-Geisser ε	Huynh-Feldt ε	Lower Bound ε
N	0.255	23.227	2	< .001	0.573	0.587	0.500
N ✻ Device	0.354	17.655	2	< .001	0.608	0.628	0.500
N ✻ Trial Type	0.824	3.290	2	0.193	0.850	0.930	0.500
N ✻ Device ✻ Trial Type	0.711	5.810	2	0.055	0.776	0.835	0.500

Classical Accuracy vs VR Arrival Accuracy

Within Subjects Effects

	Sphericity correction	Sum of Squares	df	Mean Square	F	p	η²	η²p
N	None	0.748 [a]	2.000 [a]	0.374 [a]	35.973 [a]	< .001 [a]	0.328	0.666
	Greenhouse-Geisser	0.748	1.189	0.629	35.973	< .001	0.328	0.666
Residuals	None	0.374	36.000	0.010				
	Greenhouse-Geisser	0.374	21.395	0.017				
Device	None	0.008	1.000	0.008	1.471	0.241	0.004	0.076
Residuals	None	0.102	18.000	0.006				
Trial Type	None	0.180	1.000	0.180	17.096	< .001	0.079	0.487
Residuals	None	0.190	18.000	0.011				
N ✻ Device	None	2.412×10^{-4} [a]	2.000 [a]	1.206×10^{-4} [a]	0.035 [a]	0.965 [a]	1.060×10^{-4}	0.002
	Greenhouse-Geisser	2.412×10^{-4}	1.323	1.823×10^{-4}	0.035	0.909	1.060×10^{-4}	0.002
Residuals	None	0.123	36.000	0.003				
	Greenhouse-Geisser	0.123	23.820	0.005				
N ✻ Trial Type	None	0.139 [a]	2.000 [a]	0.070 [a]	10.574 [a]	< .001 [a]	0.061	0.370
	Greenhouse-Geisser	0.139	1.127	0.124	10.574	0.003	0.061	0.370
Residuals	None	0.237	36.000	0.007				
	Greenhouse-Geisser	0.237	20.290	0.012				
Device ✻ Trial Type	None	8.966×10^{-4}	1.000	8.966×10^{-4}	0.410	0.530	3.939×10^{-4}	0.022
Residuals	None	0.039	18.000	0.002				
N ✻ Device ✻ Trial Type	None	0.005 [a]	2.000 [a]	0.003 [a]	0.751 [a]	0.479 [a]	0.002	0.040
	Greenhouse-Geisser	0.005	1.253	0.004	0.751	0.424	0.002	0.040
Residuals	None	0.128	36.000	0.004				
	Greenhouse-Geisser	0.128	22.554	0.006				

Note. Sphericity corrections not available for factors with 2 levels.

Note. Type III Sum of Squares

Within Subjects Effects

	Sphericity correction	Sum of Squares	df	Mean Square	F	p	η^2	η^2_p

[a] Mauchly's test of sphericity indicates that the assumption of sphericity is violated (p < .05).

Between Subjects Effects

	Sum of Squares	df	Mean Square	F	p
Residuals	0.403	18	0.022		

Note. Type III Sum of Squares

Descriptives

Descriptives

N	Device	Trial Type	N	Mean	SD	SE	Coefficient of variation
1	Classical	non-Match	19	0.991	0.013	0.003	0.013
		Match	19	0.966	0.041	0.009	0.043
	VR	non-Match	19	0.991	0.018	0.004	0.018
		Match	19	0.989	0.026	0.006	0.026
2	Classical	non-Match	19	0.970	0.029	0.007	0.030
		Match	19	0.931	0.077	0.018	0.083
	VR	non-Match	19	0.970	0.044	0.010	0.045
		Match	19	0.950	0.059	0.014	0.062
3	Classical	non-Match	19	0.901	0.063	0.014	0.069
		Match	19	0.786	0.148	0.034	0.188
	VR	non-Match	19	0.926	0.076	0.017	0.082
		Match	19	0.791	0.205	0.047	0.260

Descriptives plots

Device: Classical

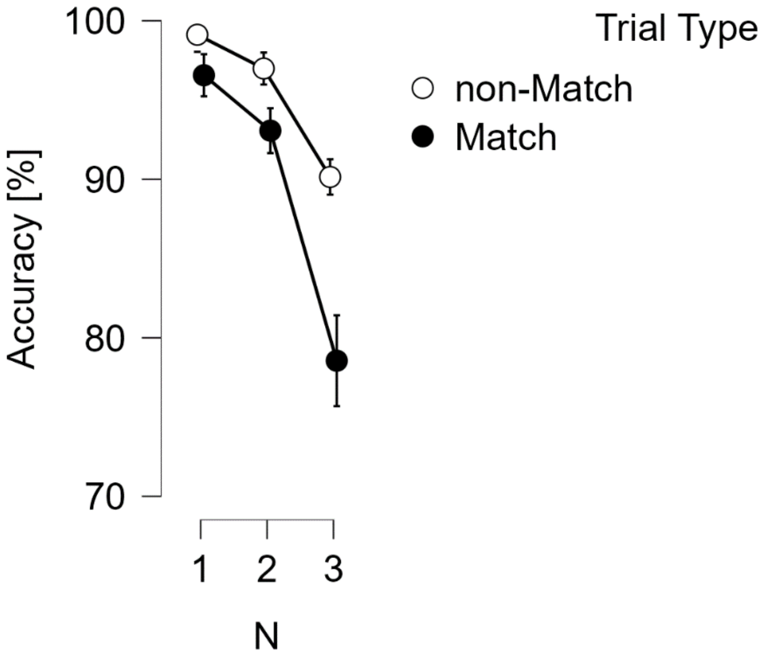

Device: VR

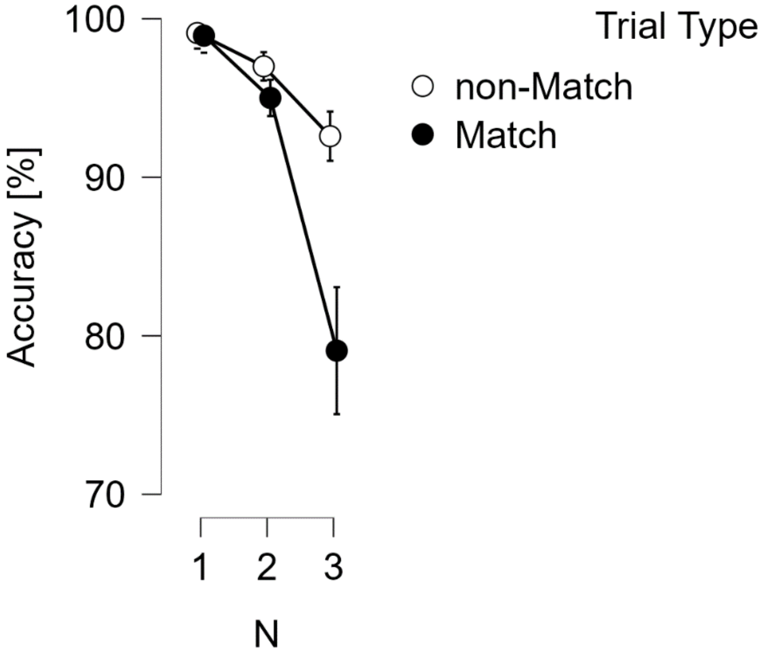

Assumption Checks

Test of Sphericity

	Mauchly's W	Approx. X^2	df	p-value	Greenhouse-Geisser ε	Huynh-Feldt ε	Lower Bound ε
N	0.317	19.510	2	< .001	0.594	0.612	0.500
N ✻ Device	0.489	12.173	2	0.002	0.662	0.694	0.500
N ✻ Trial Type	0.226	25.304	2	< .001	0.564	0.575	0.500
N ✻ Device ✻ Trial Type	0.404	15.414	2	< .001	0.627	0.651	0.500

Classical Accuracy vs VR Halfway Accuracy

Within Subjects Effects

	Sphericity correction	Sum of Squares	df	Mean Square	F	p	η^2	η^2_p
N	None	0.802 [a]	2.000 [a]	0.401 [a]	39.945 [a]	< .001 [a]	0.341	0.689
	Greenhouse-Geisser	0.802	1.246	0.644	39.945	< .001	0.341	0.689
Residuals	None	0.361	36.000	0.010				
	Greenhouse-Geisser	0.361	22.421	0.016				
Device	None	1.022×10^{-4}	1.000	1.022×10^{-4}	0.018	0.896	4.340×10^{-5}	9.816×10^{-4}
Residuals	None	0.104	18.000	0.006				
Trial Type	None	0.127	1.000	0.127	10.527	0.004	0.054	0.369
Residuals	None	0.217	18.000	0.012				
N ✻ Device	None	7.197×10^{-4} [a]	2.000 [a]	3.599×10^{-4} [a]	0.089 [a]	0.915 [a]	3.058×10^{-4}	0.005
	Greenhouse-Geisser	7.197×10^{-4}	1.355	5.312×10^{-4}	0.089	0.841	3.058×10^{-4}	0.005
Residuals	None	0.146	36.000	0.004				
	Greenhouse-Geisser	0.146	24.388	0.006				
N ✻ Trial Type	None	0.077 [a]	2.000 [a]	0.039 [a]	5.098 [a]	0.011 [a]	0.033	0.221
	Greenhouse-Geisser	0.077	1.183	0.065	5.098	0.029	0.033	0.221
Residuals	None	0.272	36.000	0.008				
	Greenhouse-Geisser	0.272	21.291	0.013				
Device ✻ Trial Type	None	0.010	1.000	0.010	2.727	0.116	0.004	0.132
Residuals	None	0.064	18.000	0.004				
N ✻ Device ✻ Trial Type	None	7.510×10^{-4} [a]	2.000 [a]	3.755×10^{-4} [a]	0.079 [a]	0.924 [a]	3.191×10^{-4}	0.004
	Greenhouse-Geisser	7.510×10^{-4}	1.197	6.272×10^{-4}	0.079	0.826	3.191×10^{-4}	0.004
Residuals	None	0.172	36.000	0.005				
	Greenhouse-Geisser	0.172	21.554	0.008				

Within Subjects Effects

	Sphericity correction	Sum of Squares	df	Mean Square	F	p	η^2	η^2_p

Note. Sphericity corrections not available for factors with 2 levels.

Note. Type III Sum of Squares

[a] Mauchly's test of sphericity indicates that the assumption of sphericity is violated (p < .05).

Between Subjects Effects

	Sum of Squares	df	Mean Square	F	p
Residuals	0.360	18	0.020		

Note. Type III Sum of Squares

Descriptives

Descriptives

N	Device	Trial Type	N	Mean	SD	SE	Coefficient of variation
1	Classical	non-Match	19	0.991	0.013	0.003	0.013
		Match	19	0.966	0.041	0.009	0.043
	VR	non-Match	19	0.982	0.024	0.006	0.024
		Match	19	0.982	0.026	0.006	0.026
2	Classical	non-Match	19	0.970	0.029	0.007	0.030
		Match	19	0.931	0.077	0.018	0.083
	VR	non-Match	19	0.957	0.052	0.012	0.054
		Match	19	0.936	0.057	0.013	0.061
3	Classical	non-Match	19	0.901	0.063	0.014	0.069
		Match	19	0.786	0.148	0.034	0.188
	VR	non-Match	19	0.880	0.107	0.025	0.122
		Match	19	0.799	0.203	0.047	0.254

Descriptives plots

Device: Classical

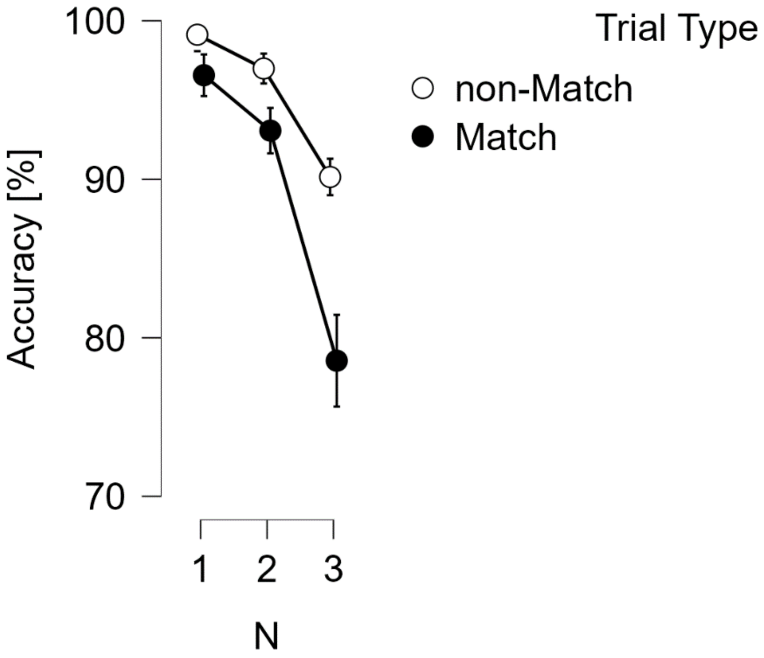

Device: VR

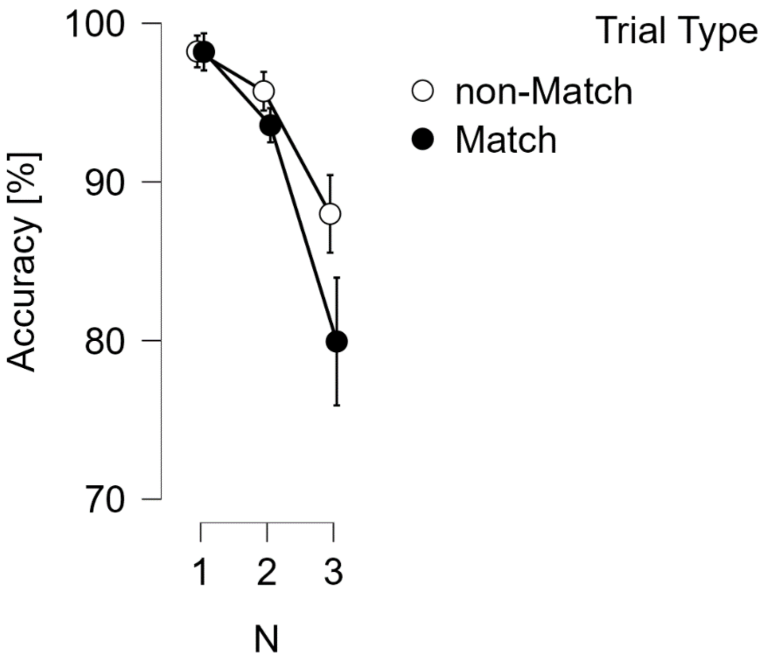

Assumption Checks

Test of Sphericity

	Mauchly's W	Approx. X^2	df	p-value	Greenhouse-Geisser ε	Huynh-Feldt ε	Lower Bound ε
N	0.394	15.817	2	< .001	0.623	0.647	0.500
N ✻ Device	0.524	10.991	2	0.004	0.677	0.713	0.500
N ✻ Trial Type	0.309	19.955	2	< .001	0.591	0.609	0.500
N ✻ Device ✻ Trial Type	0.330	18.858	2	< .001	0.599	0.618	0.500

Correct Classical RT

Within Subjects Effects

	Sphericity correction	Sum of Squares	df	Mean Square	F	p	η²	η²p
N	None	50.587 [a]	2.000 [a]	25.294 [a]	24.989 [a]	< .001 [a]	0.508	0.581
	Greenhouse-Geisser	50.587	1.178	42.939	24.989	< .001	0.508	0.581
Residuals	None	36.439	36.000	1.012				
	Greenhouse-Geisser	36.439	21.206	1.718				
Trial Type	None	3.245	1.000	3.245	13.134	0.002	0.033	0.422
Residuals	None	4.447	18.000	0.247				
N ✻ Trial Type	None	0.830	2.000	0.415	3.680	0.035	0.008	0.170
	Greenhouse-Geisser	0.830	1.956	0.424	3.680	0.036	0.008	0.170
Residuals	None	4.060	36.000	0.113				
	Greenhouse-Geisser	4.060	35.215	0.115				

Note. Sphericity corrections not available for factors with 2 levels.

Note. Type III Sum of Squares

[a] Mauchly's test of sphericity indicates that the assumption of sphericity is violated ($p < .05$).

Between Subjects Effects

	Sum of Squares	df	Mean Square	F	p
Residuals	54.180	18	3.010		

Note. Type III Sum of Squares

Descriptives

Descriptives

N	Trial Type	N	Mean	SD	SE	Coefficient of variation
1	non-Match	19	0.779	0.139	0.032	0.179
	Match	19	0.682	0.075	0.017	0.110
2	non-Match	19	1.811	0.951	0.218	0.525
	Match	19	1.358	0.586	0.134	0.432
3	non-Match	19	2.593	1.518	0.348	0.585

Descriptives

N	Trial Type	N	Mean	SD	SE	Coefficient of variation
	Match	19	2.130	1.389	0.319	0.652

Descriptives plots

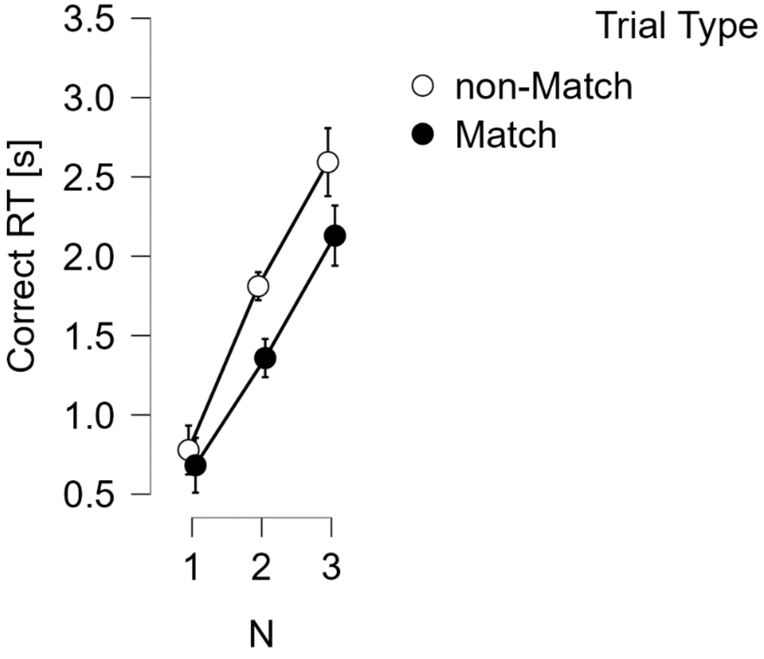

Assumption Checks

Test of Sphericity

	Mauchly's W	Approx. Χ²	df	p-value	Greenhouse-Geisser ε	Huynh-Feldt ε	Lower Bound ε
N	0.302	20.334	2	< .001	0.589	0.606	0.500
N ✻ Trial Type	0.978	0.383	2	0.826	0.978	1.000	0.500

Correct VR Arrival times

Within Subjects Effects

	Sphericity correction	Sum of Squares	df	Mean Square	F	p	η²	η²ₚ
N	None	18.254 [a]	2.000 [a]	9.127 [a]	19.873 [a]	< .001 [a]	0.425	0.525
	Greenhouse-Geisser	18.254	1.170	15.596	19.873	< .001	0.425	0.525
Residuals	None	16.534	36.000	0.459				
	Greenhouse-Geisser	16.534	21.068	0.785				
Trial Type	None	1.391	1.000	1.391	7.686	0.013	0.032	0.299
Residuals	None	3.259	18.000	0.181				
N ✻ Trial Type	None	0.662 [a]	2.000 [a]	0.331 [a]	4.157 [a]	0.024 [a]	0.015	0.188
	Greenhouse-Geisser	0.662	1.233	0.537	4.157	0.046	0.015	0.188
Residuals	None	2.868	36.000	0.080				
	Greenhouse-Geisser	2.868	22.201	0.129				

Note. Sphericity corrections not available for factors with 2 levels.

Note. Type III Sum of Squares

[a] Mauchly's test of sphericity indicates that the assumption of sphericity is violated ($p < .05$).

Between Subjects Effects

	Sum of Squares	df	Mean Square	F	p
Residuals	30.328	18	1.685		

Note. Type III Sum of Squares

Descriptives

Descriptives

N	Trial Type	N	Mean	SD	SE	Coefficient of variation
1	non-Match	19	1.191	0.242	0.056	0.204
	Match	19	1.123	0.201	0.046	0.179
2	non-Match	19	1.702	0.644	0.148	0.379
	Match	19	1.536	0.466	0.107	0.303
3	non-Match	19	2.351	1.229	0.282	0.523

Descriptives

N	Trial Type	N	Mean	SD	SE	Coefficient of variation
	Match	19	1.922	0.837	0.192	0.436

Descriptives plots

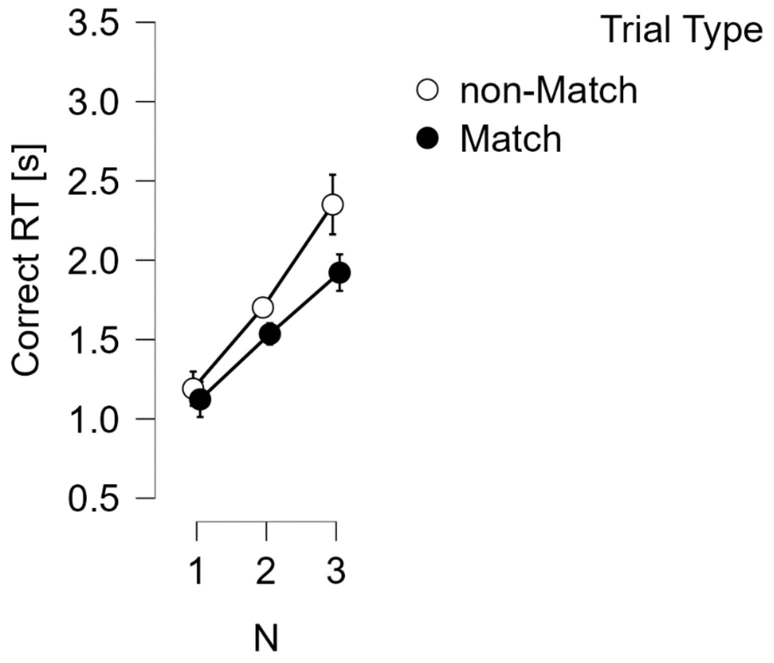

Assumption Checks

Test of Sphericity

	Mauchly's W	Approx. X²	df	p-value	Greenhouse-Geisser ε	Huynh-Feldt ε	Lower Bound ε
N	0.291	20.972	2	< .001	0.585	0.601	0.500
N ✻ Trial Type	0.378	16.518	2	< .001	0.617	0.639	0.500

Correct VR Halfway times

Within Subjects Effects

	Sphericity correction	Sum of Squares	df	Mean Square	F	p	η²	η²ₚ
N	None	11.681 [a]	2.000 [a]	5.841 [a]	21.623 [a]	< .001 [a]	0.419	0.546
	Greenhouse-Geisser	11.681	1.140	10.243	21.623	< .001	0.419	0.546
Residuals	None	9.724	36.000	0.270				
	Greenhouse-Geisser	9.724	20.527	0.474				
Trial Type	None	0.786	1.000	0.786	4.608	0.046	0.028	0.204
Residuals	None	3.070	18.000	0.171				
N ✻ Trial Type	None	0.201 [a]	2.000 [a]	0.100 [a]	1.504 [a]	0.236 [a]	0.007	0.077
	Greenhouse-Geisser	0.201	1.216	0.165	1.504	0.238	0.007	0.077
Residuals	None	2.404	36.000	0.067				
	Greenhouse-Geisser	2.404	21.896	0.110				

Note. Sphericity corrections not available for factors with 2 levels.
Note. Type III Sum of Squares
[a] Mauchly's test of sphericity indicates that the assumption of sphericity is violated (p < .05).

Between Subjects Effects

	Sum of Squares	df	Mean Square	F	p
Residuals	19.102	18	1.061		

Note. Type III Sum of Squares

Descriptives

Descriptives

N	Trial Type	N	Mean	SD	SE	Coefficient of variation
1	non-Match	19	0.943	0.131	0.030	0.139
	Match	19	0.881	0.130	0.030	0.147
2	non-Match	19	1.382	0.554	0.127	0.401
	Match	19	1.212	0.362	0.083	0.298
3	non-Match	19	1.829	0.971	0.223	0.531

Descriptives

N	Trial Type	N	Mean	SD	SE	Coefficient of variation
	Match	19	1.562	0.701	0.161	0.448

Descriptives plots

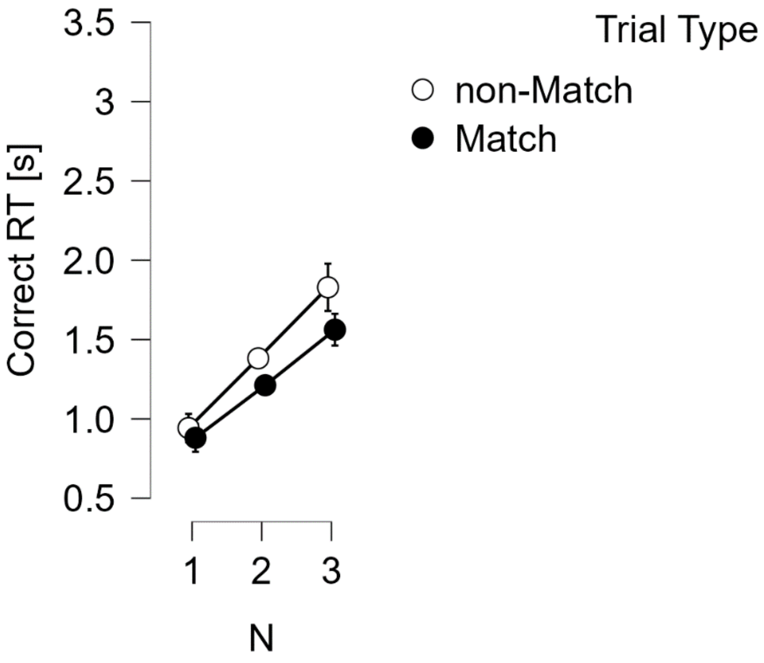

Assumption Checks

Test of Sphericity

	Mauchly's W	Approx. Χ²	df	p-value	Greenhouse-Geisser ε	Huynh-Feldt ε	Lower Bound ε
N	0.246	23.827	2	< .001	0.570	0.583	0.500
N ✻ Trial Type	0.356	17.564	2	< .001	0.608	0.629	0.500

Accuracy Classical

Within Subjects Effects

	Sphericity correction	Sum of Squares	df	Mean Square	F	p	η²	η²ₚ
N	None	0.385 [a]	2.000 [a]	0.192 [a]	35.240 [a]	< .001 [a]	0.399	0.662
	Greenhouse-Geisser	0.385	1.382	0.279	35.240	< .001	0.399	0.662
Residuals	None	0.197	36.000	0.005				
	Greenhouse-Geisser	0.197	24.877	0.008				
Trial Type	None	0.103	1.000	0.103	15.494	< .001	0.107	0.463
Residuals	None	0.120	18.000	0.007				
N ✻ Trial Type	None	0.045	2.000	0.023	7.046	0.003	0.047	0.281
	Greenhouse-Geisser	0.045	1.578	0.029	7.046	0.006	0.047	0.281
Residuals	None	0.115	36.000	0.003				
	Greenhouse-Geisser	0.115	28.408	0.004				

Note. Sphericity corrections not available for factors with 2 levels.

Note. Type III Sum of Squares

[a] Mauchly's test of sphericity indicates that the assumption of sphericity is violated (p < .05).

Between Subjects Effects

	Sum of Squares	df	Mean Square	F	p
Residuals	0.187	18	0.010		

Note. Type III Sum of Squares

Descriptives

Descriptives

N	Trial Type	N	Mean	SD	SE	Coefficient of variation
1	non-Match	19	0.991	0.013	0.003	0.013
	Match	19	0.966	0.041	0.009	0.043
2	non-Match	19	0.970	0.029	0.007	0.030
	Match	19	0.931	0.077	0.018	0.083
3	non-Match	19	0.901	0.063	0.014	0.069

Descriptives

N	Trial Type	N	Mean	SD	SE	Coefficient of variation
	Match	19	0.786	0.148	0.034	0.188

Descriptives plots

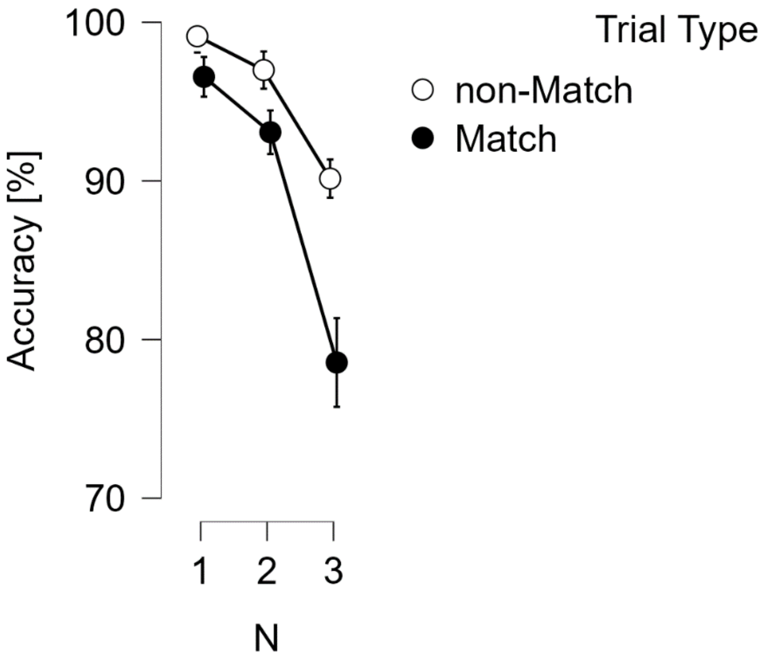

Assumption Checks

Test of Sphericity

	Mauchly's W	Approx. X²	df	p-value	Greenhouse-Geisser ε	Huynh-Feldt ε	Lower Bound ε
N	0.553	10.074	2	0.006	0.691	0.730	0.500
N ✻ Trial Type	0.733	5.286	2	0.071	0.789	0.852	0.500

Accuracy VR Arrival

Within Subjects Effects

	Sphericity correction	Sum of Squares	df	Mean Square	F	p	η²	η²ₚ
N	None	0.363 [a]	2.000 [a]	0.182 [a]	21.757 [a]	< .001 [a]	0.302	0.547
	Greenhouse-Geisser	0.363	1.227	0.296	21.757	< .001	0.302	0.547
Residuals	None	0.300	36.000	0.008				
	Greenhouse-Geisser	0.300	22.088	0.014				
Trial Type	None	0.078	1.000	0.078	12.844	0.002	0.065	0.416
Residuals	None	0.109	18.000	0.006				
N ✻ Trial Type	None	0.100 [a]	2.000 [a]	0.050 [a]	7.164 [a]	0.002 [a]	0.083	0.285
	Greenhouse-Geisser	0.100	1.071	0.093	7.164	0.013	0.083	0.285
Residuals	None	0.251	36.000	0.007				
	Greenhouse-Geisser	0.251	19.278	0.013				

Note. Sphericity corrections not available for factors with 2 levels.

Note. Type III Sum of Squares

[a] Mauchly's test of sphericity indicates that the assumption of sphericity is violated ($p < .05$).

Between Subjects Effects

	Sum of Squares	df	Mean Square	F	p
Residuals	0.318	18	0.018		

Note. Type III Sum of Squares

Descriptives

Descriptives

N	Trial Type	N	Mean	SD	SE	Coefficient of variation
1	non-Match	19	0.991	0.018	0.004	0.018
	Match	19	0.989	0.026	0.006	0.026
2	non-Match	19	0.970	0.044	0.010	0.045
	Match	19	0.950	0.059	0.014	0.062
3	non-Match	19	0.926	0.076	0.017	0.082

Descriptives

N	Trial Type	N	Mean	SD	SE	Coefficient of variation
	Match	19	0.791	0.205	0.047	0.260

Descriptives plots

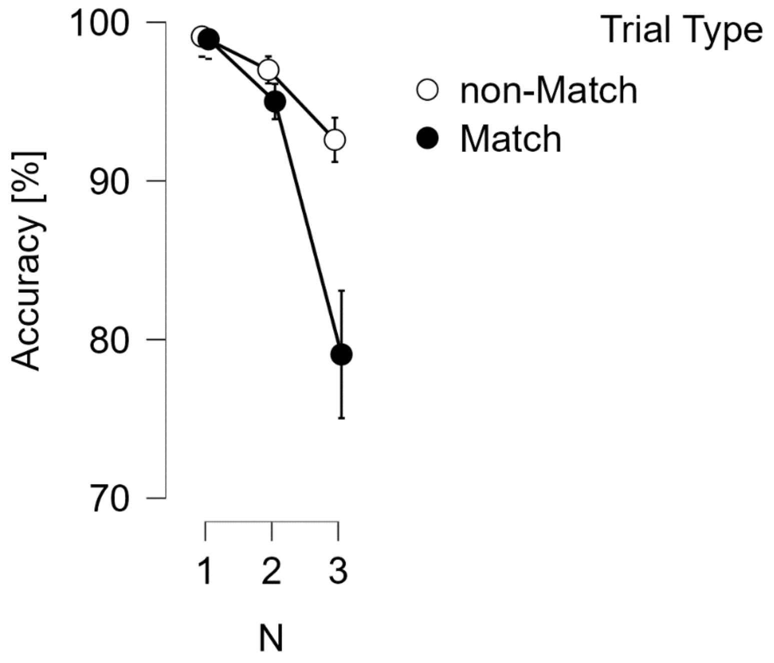

Assumption Checks

Test of Sphericity

	Mauchly's W	Approx. X^2	df	p-value	Greenhouse-Geisser ε	Huynh-Feldt ε	Lower Bound ε
N	0.370	16.896	2	< .001	0.614	0.635	0.500
N ✻ Trial Type	0.133	34.344	2	< .001	0.536	0.542	0.500

Accuracy VR Halfway

Within Subjects Effects

	Sphericity correction	Sum of Squares	df	Mean Square	F	p	η²	η²p
N	None	0.418 [a]	2.000 [a]	0.209 [a]	24.212 [a]	< .001 [a]	0.325	0.574
	Greenhouse-Geisser	0.418	1.308	0.319	24.212	< .001	0.325	0.574
Residuals	None	0.311	36.000	0.009				
	Greenhouse-Geisser	0.311	23.544	0.013				
Trial Type	None	0.033	1.000	0.033	3.697	0.070	0.026	0.170
Residuals	None	0.161	18.000	0.009				
N ✻ Trial Type	None	0.033 [a]	2.000 [a]	0.016 [a]	1.796 [a]	0.181 [a]	0.026	0.091
	Greenhouse-Geisser	0.033	1.117	0.029	1.796	0.196	0.026	0.091
Residuals	None	0.329	36.000	0.009				
	Greenhouse-Geisser	0.329	20.106	0.016				

Note. Sphericity corrections not available for factors with 2 levels.

Note. Type III Sum of Squares

[a] Mauchly's test of sphericity indicates that the assumption of sphericity is violated (p < .05).

Between Subjects Effects

	Sum of Squares	df	Mean Square	F	p
Residuals	0.277	18	0.015		

Note. Type III Sum of Squares

Descriptives

Descriptives

N	Trial Type	N	Mean	SD	SE	Coefficient of variation
1	non-Match	19	0.982	0.024	0.006	0.024
	Match	19	0.982	0.026	0.006	0.026
2	non-Match	19	0.957	0.052	0.012	0.054
	Match	19	0.936	0.057	0.013	0.061
3	non-Match	19	0.880	0.107	0.025	0.122
	Match	19	0.799	0.203	0.047	0.254

Descriptives plots

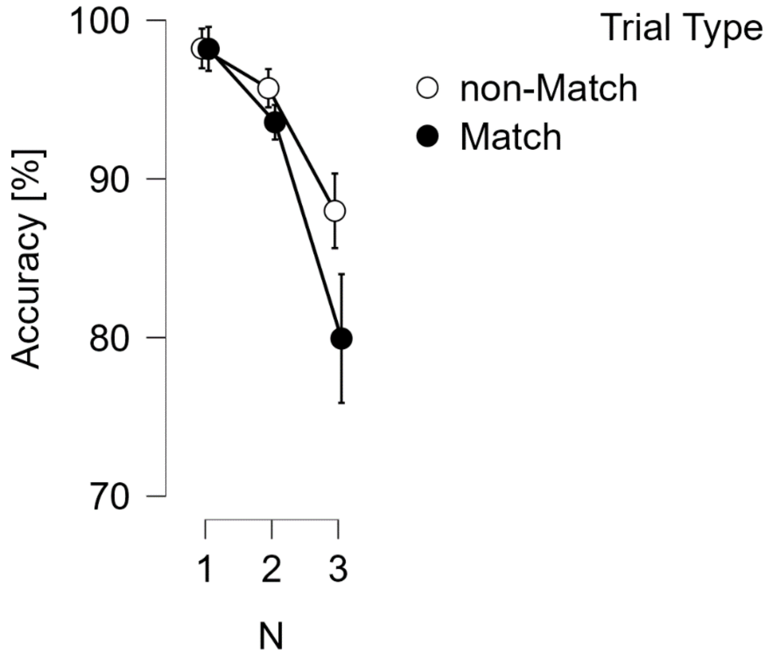

Assumption Checks

Test of Sphericity

	Mauchly's W	Approx. Χ²	df	p-value	Greenhouse-Geisser ε	Huynh-Feldt ε	Lower Bound ε
N	0.471	12.801	2	0.002	0.654	0.685	0.500
N ✻ Trial Type	0.209	26.575	2	< .001	0.558	0.569	0.500
